# Whole genome and transcriptome maps of the entirely black native Korean chicken breed *Yeonsan Ogye*

**DOI:** 10.1093/gigascience/giy086

**Published:** 2018-07-11

**Authors:** Jang-il Sohn, Kyoungwoo Nam, Hyosun Hong, Jun-Mo Kim, Dajeong Lim, Kyung-Tai Lee, Yoon Jung Do, Chang Yeon Cho, Namshin Kim, Han-Ha Chai, Jin-Wu Nam

**Affiliations:** 1 Department of Life Science, Hanyang University, Seoul, 133-791, Republic of Korea; 2 Research Institute for Convergence of Basic Sciences, Hanyang University, Seoul, 133-791, Republic of Korea; 3 Department of Animal Science and Technology, Chung-Ang University, Anseong, Gyeonggi-do, 17546, Republic of Korea; 4 Department of Animal Biotechnology & Environment, National Institute of Animal Science, RDA, Wanju, 55365, Republic of Korea; 5 Animal Genetic Resource Research Center, National Institute of Animal Science, RDA, Namwon, 55717, Republic of Korea; 6 Personalized Genomic Medicine Research Center, KRIBB, Daejeon, 34141, Republic of Korea; 7 College of Pharmacy, Chonnam National University, Kwangju, 61186, Republic of Korea

**Keywords:** *Gallus gallus domesticus*, *Yeonsan Ogye*, whole genome *de novo* assembly, transcriptome maps, hyperpigmentation

## Abstract

**Background:**

*Yeonsan Ogye* (*YO*), an indigenous Korean chicken breed (*Gallus gallus domesticus*), has entirely black external features and internal organs. In this study, the draft genome of *YO* was assembled using a hybrid *de novo* assembly method that takes advantage of high-depth Illumina short reads (376.6X) and low-depth Pacific Biosciences (PacBio) long reads (9.7X).

**Findings:**

The contig and scaffold NG50s of the hybrid *de novo* assembly were 362.3 Kbp and 16.8 Mbp, respectively. The completeness (97.6%) of the draft genome (Ogye_1.1) was evaluated with single-copy orthologous genes using Benchmarking Universal Single-Copy Orthologs and found to be comparable to the current chicken reference genome (galGal5; 97.4%; contigs were assembled with high-depth PacBio long reads (50X) and scaffolded with short reads) and superior to other avian genomes (92%–93%; assembled with short read-only or hybrid methods). Compared to galGal4 and galGal5, the draft genome included 551 structural variations including the fibromelanosis (FM) locus duplication, related to hyperpigmentation. To comprehensively reconstruct transcriptome maps, RNA sequencing and reduced representation bisulfite sequencing data were analyzed from 20 tissues, including 4 black tissues (skin, shank, comb, and fascia). The maps included 15,766 protein-coding and 6,900 long noncoding RNA genes, many of which were tissue-specifically expressed and displayed tissue-specific DNA methylation patterns in the promoter regions.

**Conclusions:**

We expect that the resulting genome sequence and transcriptome maps will be valuable resources for studying domestic chicken breeds, including black-skinned chickens, as well as for understanding genomic differences between breeds and the evolution of hyperpigmented chickens and functional elements related to hyperpigmentation.

## Background

The *Yeonsan Ogye* (*YO*), a designated natural monument of Korea (no. 265), is an indigenous Korean chicken breed that is notable for its entirely black plumage, skin, beak, comb, eyes, shank, claws, and internal organs [1]. In terms of its plumage and body color, as well as its number of toes, this unique chicken breed resembles the indigenous Indonesian chicken breed *Ayam cemani* [2–4]. *YO* also has some morphological features that are similar to those of the *Silkie* fowl, with the exception of the *Silkie*’s veiled black walnut comb and hair-like, fluffy plumage that is white or variably colored [5, 6]. Although the exact origin of the *YO* breed has not yet been clearly defined, its features and medicinal usages were recorded in *Dongui Bogam* [7], a traditional Korean medical encyclopedia compiled and edited by Heo Jun in 1613.

To date, a number of avian genomes from both domestic and wild species have been assembled and compared, revealing genomic signatures associated with the domestication process and genomic differences that provide an evolutionary perspective [8]. The chicken reference genome was first assembled using the red junglefowl [9], first domesticated at least 5,000 years ago in Asia; the latest version of the reference genome was released in 2015 (galGal5, GenBank Assembly ID GCA_000002315.3) [10]. However, because domesticated chickens exhibit diverse morphological features, including skin and plumage colors, the genome sequences of unique breeds are necessary for understanding their characteristic phenotypes through analyses of single nucleotide polymorphisms (SNPs), insertions and deletions (INDELs), structural variations (SVs), and coding and noncoding transcriptomes. Here, we provide the first version of the *YO* genome (Ogye_1.1), which includes annotations of large SVs, SNPs, INDELs, and repeats, as well as coding and noncoding transcriptome maps along with DNA methylation landscapes across 20 *YO* tissues.

## Data Description

### Sample collection

**Figure 1:**
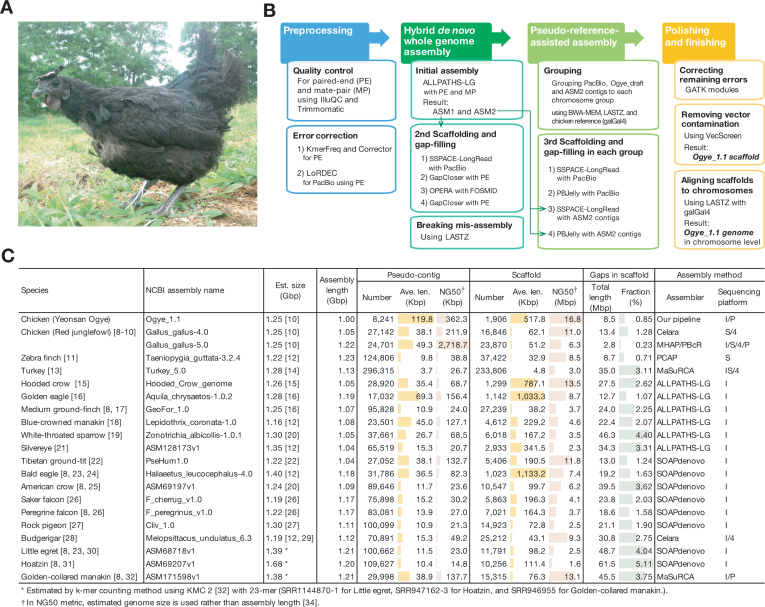
**(A)** A photograph of *Yeonsan Ogye* (*YO*) taken before sampling. **(B)** Hybrid genome assembly pipeline comprising four steps, each of which utilizes a different set of sequencing reads (see Table 1). Detailed methods for breaking misassembly and pseudo-reference-assisted assembly are depicted in Supplementary Figs. S2 and S3. (**C)** The NG50 and average length of pseudo contigs and scaffolds for the Ogye_1.1 and other avian genomes, generated using the indicated assembly methods (in the last column, sequencing platforms are designated as follows: I: Illumina, P: Pacific Biosciences, S: Sanger, 4: Roche454).

An 8-month-old *YO* chicken (object no. 02127), obtained from the Animal Genetic Resource Research Center of the National Institute of Animal Science (Namwon, Korea), was used in the study (Fig. 1A; [8–34]). All sequencing data in this study (including data from whole genome sequencing, RNA sequencing [RNA-seq], and reduced representation bisulfite sequencing [RRBS]) were obtained from this sample bird. The protocols for the care and experimental use of *YO* were reviewed and approved by the Institutional Animal Care and Use Committee of the National Institute of Animal Science (no. 2014-080). *YO* management, treatment, and sample collection took place at the National Institute of Animal Science.

### Whole-genome sequencing

Genomic DNA was extracted from blood using the Wizard DNA extraction kit [35] and prepared for DNA sequencing library construction. According to the DNA fragment (insert) size, three different library types were constructed: paired-end libraries for small inserts (280 and 500 bp), mate-pair libraries for large inserts (3, 5, 8, and 10 Kbp), and FSMID libraries for very large inserts (40 Kbp) using Illumina's protocols (Illumina, San Diego, CA, USA) (Table 1). The constructed libraries were sequenced using Illumina's Hiseq2000 platform. In total, 376.6X raw Illumina short reads (100.2X from the small insert libraries and 276.4X from the large insert libraries) were generated (Table 1 and Supplementary Table S1). To fill gaps and improve the scaffold N50, 9.7X Pacific Biosciences (PacBio) long reads were additionally sequenced using the PacBio RS II platform with P6C4 chemistry; the average length of the long reads was 6 Kbp (Table 1).

**Table 1:** Summary of whole-genome sequencing data (estimated genome size 1.25 Gbp)

			Raw data				Preprocessed data							
								Usage of data (coverage, X)						
Platform	Library type	Insert-size	Number of read (10^6^)	Total base (Gbp)	Coverage (X)	SRA accession	Coverage (X)	SEC	ASM1	ASM2	SCF	GF	SV	SIC
Illumina	Paired-end	280 bp	259.2	39.0	31.2	SRR6189087	21.4	O	O				O	O
HiSeq 2000			248.9	37.4	29.9	SRR6189084	20.5	O		O		O	O	O
		500 bp	87.1	13.1	10.5	SRR6189095	4.8	O	O				O	O
			94.4	14.2	11.4	SRR6189097	5.2	O	O			O	O	O
			28.1	4.2	3.4	SRR6189096	1.3	O	O				O	O
			28.3	4.3	3.4	SRR6189098	1.2	O		O		O	O	O
			29.2	4.4	3.5	SRR6189082	1.8	O		O			O	O
			57.4	8.6	6.9	SRR6189094	4.5	O	O			O	O	O
	Paired-end total		832.5	125.2	100.2		60.7	60.7	37.2	23.5	–	31.4	60.7	60.7
	Mate-pair	3 Kbp	293.1	43.6	34.9	SRR6189093	23.6			O			O	
			270.0	40.2	32.1	SRR6189083	21.6		O					
		5 Kbp	229.6	34.2	27.4	SRR6189081	16.9		O				O	
			212.8	31.7	25.4	SRR6189088	15.7			O				
		8 Kbp	273.1	40.7	32.6	SRR6189085	20.2		O				O	
			270.5	40.4	32.3	SRR6189086	19.7			O				
		10 Kbp	338.2	50.4	40.3	SRR6189091	26.7			O			O	
			315.9	47.1	37.7	SRR6189092	25.3		O					
		40 Kbp^a^	169.9	17.2	13.7	SRR6189089	10.7				O			
	Mate-pair total		2,373.2	345.5	276.4		180.4	–	84.0	85.7	10.7	–	87.4	–
PacBio RS II	Long-read	6 Kbp^b^	1.7	12.1	9.7	SRR6189090	9.3				O	O		
Illumina total			3,205.7	470.7	376.6		241.1	60.7	121.2	109.2	20.0	40.7	148.1	
Illumina + PacBio total			3,207.4	482.8	386.3		250.4	60.7	121.2	109.2	29.3	50.0	148.1	

a Fosmid

b Average read length.

Abbreviations: ASM1: initial ALLPATHS-LG assembly; ASM2: additional ALLPATHS-LG assembly; GF: gap-filling; SCF: scaffolding; SEC: sequencing error correction; SIC: SNP/INDEL calling; SV: structural variation detection;.

### Whole transcriptome sequencing

Total RNAs were extracted from 20 tissues using 80% EtOH and TRIzol (Sigma-Aldrich, St. Louis, MO, USA). The RNA concentration was checked using Quant-IT RiboGreen (Invitrogen, Carlsbad, CA, USA). To assess the integrity of the total RNA, samples were run on the Agilent 2200 TapeStation system (Agilent Technologies, Waldbronn, Germany). Only high-quality RNA samples (RNA integrity number ≥7.0) were used for RNA-seq library construction. Each library was independently prepared with 300 ng of total RNA using an Illumina TruSeq Stranded Total RNA Sample Prep Kit (Illumina, San Diego, CA, USA). The rRNA in the total RNA was depleted using a Ribo-Zero kit. After rRNA depletion, the remaining RNA was purified, fragmented, and primed for cDNA synthesis. The cleaved RNA fragments were copied into the first cDNA strand using reverse transcriptase and random hexamers. This step was followed by second strand cDNA synthesis using DNA polymerase I, RNase H, and dUTP. The resulting cDNA fragments then underwent an end-repair process, the addition of a single “A” base, after which adapters were ligated. The products were purified and enriched with polymerage chein reaction (PCR) to create the final cDNA library. The libraries were quantified using qPCR according to the qPCR Quantification Protocol Guide (KAPA Library Quantification kits for Illumina sequencing platforms) and the integrity of the cDNA libraries was examined using the Agilent 2200 TapeStation system. In sum, about 1.5 billion RNA-seq reads were sequenced from the following 20 tissues from the same bird: breast, liver, bone marrow, fascia, cerebrum, gizzard, mature and immature eggs, comb, spleen, cerebellum, gallbladder, kidney, heart, uterus, pancreas, lung, skin, eye, and shank (Table 2).

**Table 2:** Sequencing and mapping summary of RNA-seq data

	Paired end			Single end		
Samples	No. of reads	Mapping rate, %	SRA accession	No. of reads	Mapping rate, %	SRA accession
Breast	34,893,064	92.05	SRX3223583	43,294,022	90.70	SRX3223603
Liver	33,476,266	85.75	SRX3223584	48,032,813	85.81	SRX3223604
Bone marrow	30,975,506	85.00	SRX3223585	40,286,974	87.99	SRX3223605
Fascia	33,316,764	84.61	SRX3223586	42,425,452	87.93	SRX3223606
Cerebrum	30,887,821	89.95	SRX3223587	46,455,658	92.32	SRX3223607
Gizzard	31,537,118	84.00	SRX3223588	38,689,871	85.82	SRX3223608
Immature egg	32,009,437	87.73	SRX3223589	32,048,703	87.80	SRX3223609
Comb	31,936,332	85.34	SRX3223590	37,985,049	87.76	SRX3223610
Spleen	28,946,777	89.70	SRX3223591	38,704,448	89.33	SRX3223611
Mature egg	30,873,699	91.98	SRX3223592	40,650,664	92.17	SRX3223612
Cerebellum	30,798,145	93.53	SRX3223593	39,940,946	93.34	SRX3223613
Gallbladder	35,862,229	84.83	SRX3223594	35,423,339	87.06	SRX3223614
Kidney	29,953,007	87.25	SRX3223595	39,894,009	89.99	SRX3223615
Heart	30,986,431	94.14	SRX3223596	45,951,338	91.49	SRX3223616
Uterus	33,444,002	91.89	SRX3223597	46,650,355	90.63	SRX3223617
Pancreas	30,595,568	82.52	SRX3223598	47,361,192	84.35	SRX3223618
Lung	31,533,498	87.63	SRX3223599	45,552,982	92.34	SRX3223619
Skin	34,442,464	82.36	SRX3223600	41,934,970	84.00	SRX3223620
Eye	33,006,509	89.21	SRX3223601	44,044,630	91.82	SRX3223621
Shank	28,643,334	94.07	SRX3223602	47,716,995	79.86	SRX3223622

### Reduced representation bisulfite sequencing

RRBS libraries were prepared following Illumina's RRBS protocol. To prepare the libraries, 5 µg of genomic DNA that had been digested with the restriction enzyme MspI and purified with a QIAquick PCR purification kit (QIAGEN, Hilden, Germany); a TruSeq Nano DNA Library Prep Kit (Illumina, San Diego, CA, USA) was used. Eluted DNA fragments were end-repaired, extended on the 3' end with an “A,” and ligated with Truseq adapters. The products, which ranged from 175 to 225 bp in length (insert DNA of 55–105 bp plus adaptors of 120 bp), were excised from 2% (w/v) Low Range Ultra Agarose gel (Biorad, Hercules, CA, USA) and purified using the QIAquick gel extraction protocol. The purified DNA underwent bisulfite conversion using the EpiTect Bisulfite Kit (Qiagen, 59 104). The bisulfite-converted DNA libraries were amplified by PCR (four cycles) using PfuTurbo Cx DNA polymerase (Agilent, 600 410). The quantity of the DNA libraries was then examined using qPCR, and the integrity was examined using the Agilent 2200 TapeStation system. The final product was sequenced using the HiSeq 2500 platform (Illumina, San Diego, CA, USA). Ultimately, 123 million RRBS reads were produced from 20 tissues from the same bird (see Table 3).

**Table 3:** Sequencing and mapping summary of RRBS data

Samples	No. of reads	Mapping rate, %	SRA accession
Breast	6,042,106	68.90	SRX3223667
Liver	6,744,208	74.20	SRX3223668
Bone marrow	5,736,011	72.00	SRX3223669
Fascia	5,720,194	68.90	SRX3223670
Cerebrum	6,078,989	70.00	SRX3223671
Gizzard	5,731,878	69.40	SRX3223672
Immature egg	6,741,258	67.70	SRX3223673
Comb	5,948,687	72.90	SRX3223674
Spleen	6,307,517	77.60	SRX3223675
Mature egg	6,246,607	69.20	SRX3223676
Cerebellum	6,291,610	68.20	SRX3223677
Gallbladder	5,738,180	70.10	SRX3223678
Kidney	5,470,502	68.60	SRX3223679
Heart	5,462,739	69.40	SRX3223680
Uterus	6,046,764	67.90	SRX3223681
Pancreas	7,100,215	70.30	SRX3223682
Lung	5,640,120	67.60	SRX3223683
Skin	7,226,309	72.40	SRX3223684
Eye	6,956,141	71.90	SRX3223685
Shank	5,924,463	74.20	SRX3223686

## Hybrid Whole-Genome Assembly

The Ogye_1.1 genome was assembled using our hybrid genome assembly pipeline, employing the following four steps: 1) preprocessing, 2) hybrid *de novo* assembly, 3) pseudo-reference-assisted assembly, and 4) polishing and finishing (Fig. 1B and Supplementary Fig. S1). In the preprocessing step, reads in which ≥30% of the nucleotides had a Phred score <20 were excluded using the NGS QC Toolkit (IlluQC_PRLL.pl) [36]; the adaptor sequences of the remaining reads were removed using Trimmomatic (Trimmomatic, RRID:SCR_011848) [37]; and three nucleotides at the 5’ end and five nucleotides at the 3’ end of the reads were trimmed using the NGS QC Toolkit (TrimmingReads.pl). After quality control, the sequencing errors in the Illumina paired-end short reads were corrected using KmerFreq and Corrector [38]. After these steps, 241.1X preprocessed reads were obtained for whole-genome assembly. In turn, using the corrected short reads, the sequencing errors in the PacBio long reads were corrected using LoRDEC [39].

In the hybrid *de novo* genome assembly, the initial assembly (ASM1) was done with 121.2X error-corrected short reads from the paired-end and mate-pair libraries (see Table 1) using ALLPATHS-LG (ALLPATHS-LG, RRID:SCR_010742) [40] with the default option, producing contigs and scaffolds with N50 lengths of 53.6 Kbp and 10.7 Mbp, respectively (Fig. 1B; Supplementary Fig. S1). Additionally, another assembly (ASM2) was built with 109.2X paired-end and mate-pair reads that were unused in the initial assembly (see Table 1) using ALLPATHS-LG, resulting in 34,539 contigs with an N50 length of 59.2 Kbp. The resulting ASM2 contigs were then subjected to the pseudo-reference-assisted assembly step. In the second round of scaffolding and gap-filling (after the first scaffolding and gap-filling done during ASM1), the ASM1 scaffolds were connected with corrected PacBio long reads using SSPACE-LongRead [41], and gaps within and between scaffolds were examined with error-corrected short reads using GapCloser (GapCloser, RRID:SCR_015026) [38]. Then, the gap-filled scaffolds were connected again with FOSMID reads using OPERA [42], and the remaining gaps were re-examined with error-corrected short reads using GapCloser, resulting in scaffolds with an N50 length of 27.8 Mbp. However, some misassemblies (as illustrated in Supplementary Fig. S2A) were found by alignment of the resulting scaffolds with the galGal4 genome (GenBank assembly accession GCA_000002315.2) using LASTZ [43]. During an analysis of the resulting alignments, 30 misassemblies were detected and broken at each break point, as described in Supplementary Fig. S2. Breaking scaffolds at the break points resulted in a scaffold N50 length of 18.7 Mbp (Supplementary Fig. S1). For contigs, we considered a pseudo contig, broken at positions where two or more contiguous Ns appeared in scaffolds, resulting in a pseudo contig N50 of 108.6 Kbp.

In the pseudo-reference-assisted assembly step, error-corrected PacBio long reads and ASM2 contigs were utilized to reduce the topological complexity of the assembly graphs [44] (Fig. 1B). Because even scaffolding with long reads can be affected by repetitive sequences, the scaffolds mapped to each chromosome were transformed into a hierarchical bipartite graph to minimize the influence of repetitive sequences using TSRATOR [45] (Supplementary Fig. S3). In detail, error-corrected PacBio reads and ASM2 contigs were mapped to the scaffolds using BWA-MEM and, in turn, the scaffolds were mapped to the galGal4 genome using LASTZ to build the hierarchical bipartite graph. Using the hierarchical bipartite graphs, all scaffolds, PacBio reads, and ASM2 contigs were finally grouped to each chromosome. Based on these results, a third round of scaffolding and gap-filling was performed with the long reads and the ASM2 contigs in each chromosome group using SSPACE-LongRead and PBJelly (PBJelly, RRID:SCR_012091) [46], respectively, resulting in a scaffold N50 of 21.2 Mbp with 0.85% gaps (Supplementary Fig. S1).

In the last step, nucleotide errors or ambiguities were corrected using the GATK (GATK, RRID:SCR_001876) pipeline [47] with paired-end reads. In turn, any vector contamination was removed using VecScreen with the UniVec database [48] (Fig. 1B), resulting in 506.3 Kbp and 21.2 Mbp contig and scaffold N50 lengths, respectively. The final assembly results (Ogye_1.1 scaffold) showed that the gap percentage and (pseudo-)contig N50 were significantly improved, from 1.87% and 53.6 Kbp in the initial assembly to 0.85% and 506.3 Kbp in the final assembly, respectively (Supplementary Fig. S1). Using the estimated chicken genome size (1.25 Gbp [10]), Ogye_1.1 scaffold's contig and scaffold NG50 lengths were estimated at 362.3 Kbp and 16.8 Mbp, respectively (Fig. 1C). The complete genome sequence at the chromosome level was built by connecting the final scaffolds in their order of appearance in each chromosome with the introduction of 100 Kbp “N” gaps between them (Supplementary Fig. S4) (see [79]). To evaluate its completeness, the Ogye_1.1 genome was compared to the galGal4 (short-read-based assembly) and galGal5 (long-read-based assembly) genomes, with respect to 2,586 conserved vertebrate genes, using Benchmarking Universal Single-Copy Ortholog (BUSCO) (BUSCO, RRID:SCR_015008) [49] with OrthoDB v9 (OrthoDB, RRID:SCR_011980) [50]. The Ogye_1.1 genome contained more complete single-copy BUSCO genes (Table 4).

**Table 4:** Comparison of genome completeness using BUSCO

		Complete			
Species	Assembly name	Single-copy, %	Duplication, %	Fragment, %	Missing, %
Chicken	Ogye_1.1	97.60	0.50	0.90	1.00
	Gallus_gallus-4.0	96.90	0.90	1.10	1.10
	Gallus_gallus-5.0	97.40	0.90	0.70	1.00
Turkey	Turkey_5.0	93.70	0.50	4.10	1.70
Duck	BGI_duck_1.0	92.60	0.40	4.80	2.20
Zebra finch	Taeniopygia_guttata-3.2.4	93.60	2.20	2.70	1.50

## Large Structural Variations

When the Ogye_1.1 genome was compared to galGal4 and galGal5 using LASTZ [43], putative large SVs (>1 Kbp) were detected for each reference genome, and they were validated by four different SV prediction programs (Delly, Lumpy, FermiKit, and novoBreak) [51–54] (Supplementary Fig. S5 and Table S2). SVs, validated by at least one program, included 185 deletions, 180 insertions, 158 duplications, 23 inversions, and 5 intra- or inter-chromosomal translocations. A total of 290 and 447 distinct SVs were detected relative to galGal4 and galGal5, respectively, suggesting that either reference assembly could include misassemblies.

**Figure 2:**
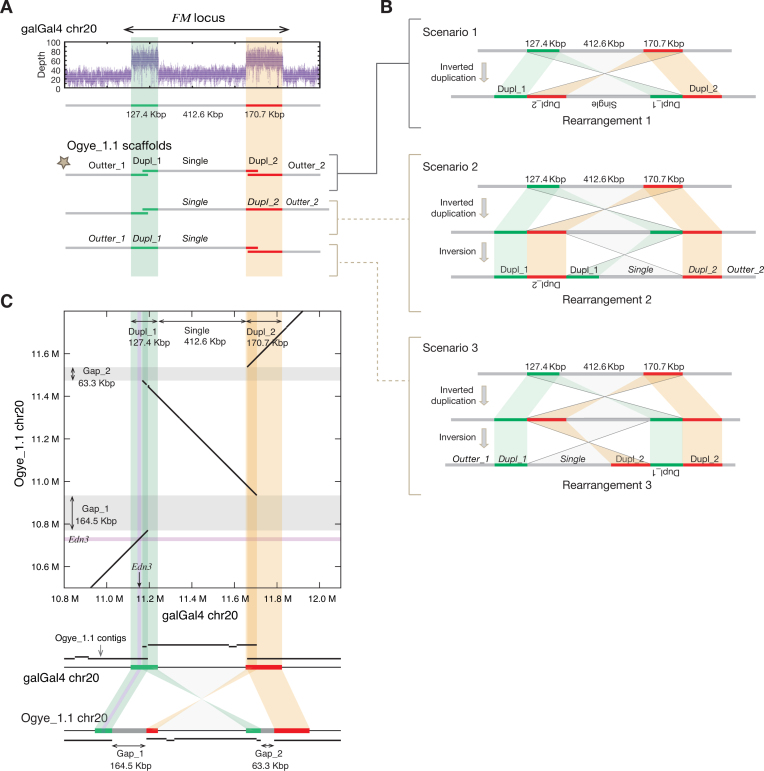
**(A)** The read depth of a locus on chromosome 20 is shown in the top panel, and the continuous/discontinuous patterns between mapped scaffolds are shown in the bottom panel. The star indicates the pattern that is discontinuous on both sides, validated in **(C)**. (**B)** Three possible scenarios were developed based on the overlap patterns. The green and red lines indicated two duplicated genomic loci (Dupl_1 and Dupl_2, respectively) including the FM locus. Scenario 1 consists of a one-step rearrangement—an inverted duplication—whereas scenarios 2 and 3 consist of a simultaneous rearrangement of an inverted duplication and an inversion. The three rearrangements were suggested in a previous study [3]. (**C)** A comparison of the FM locus in galGal4 and the Ogye draft genome with aligned contigs (black lines) in each scaffold. The gray bands indicate the estimated gaps between contigs. The estimated sizes of Gap_1 and Gap_2 are 164.5 Kbp and 63.3 Kbp, respectively. The purple lines in the box indicate the *edn3* gene locus and the green and yellow shades indicate the duplicated regions (Dupl_1 and Dupl_2, respectively). The dark green and yellow shades indicate the discontinuous regions between scaffolds.

Although the fibromelanosis (FM) locus, which contains the hyperpigmentation-related *edn3* gene, is known to be duplicated in the genomes of certain hyperpigmented chicken breeds, such as *Silkie* and *Ayam cemani* [3, 6], the exact structure of the duplicated FM locus in such breeds has not been completely resolved due to its large size (∼1 Mbp). A previous study, using conventional PCR assays, suggested three possible rearrangements at the FM locus [3]. To understand more about the mechanism of FM locus rearrangement in the Ogye_1.1 genome, the FM loci from *YO* and galGal4 were compared with mapped paired-end and mate-pair reads. A doubled read depth at two loci including the FM locus was detected in *YO*, indicating that the loci had been duplicated (Fig. 2A top). As previously reported [3, 6], our paired-end and mate-pair reads of *YO*’s FM locus were discordantly mapped to the galGal4 FM locus (Supplementary Fig. S6). The intervening region between the two duplicated regions was estimated to be 412.6 Kbp in length in the Ogye_1.1 genome. Based on these results, we propose three possible scenarios that might have produced the FM locus rearrangement (Fig. 2B). To discern which rearrangement best fits our results, the FM loci from galGal4 and the Ogye draft were compared with the resulting scaffolds, showing an inverted duplication with discontinued scaffolds at both duplicated regions (Fig. 2A, 2C). The results, with a discontinued scaffold on both sides, support rearrangement 1 rather than rearrangement 2 or 3, which have a discontinued scaffold on only one side. Although rearrangement 1 needs to be further validated, the FM locus in the Ogye_1.1 genome was updated according to the first rearrangement (Fig. 2C). Given the resulting alignment, the sizes of Gap_1 and Gap_2 were estimated to be 164.5 Kbp and 63.3 Kbp, respectively.

## Annotations

### Repeats

**Figure 3:**
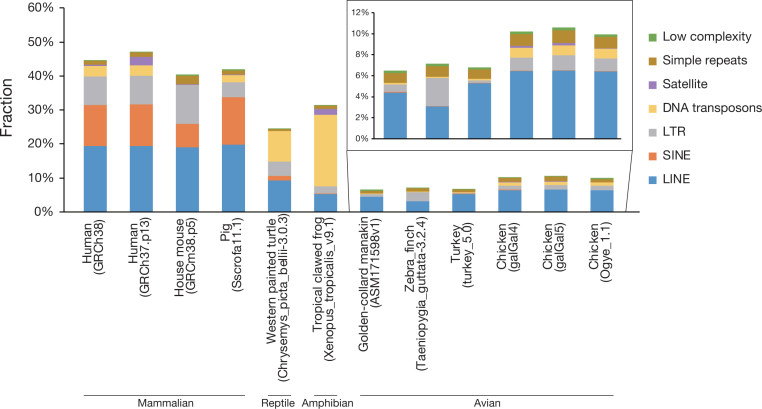
Composition of repeat elements in different assemblies of avian, amphibian, reptile, and mammalian genomes. The repeats in unplaced scaffolds were not considered.

Repeat elements in the Ogye_1.1 and other genomes (human, mouse, pig, western painted turtle, tropical clawed frog, zebra finch, turkey, and chicken) were predicted by a reference-guided approach using RepeatMasker (RepeatMasker, RRID:SCR_012954) [55] with Repbase libraries [56]. In the Ogye_1.1 genome, 205,684 retro-transposable elements (7.65%), including long interspersed nuclear elements (LINEs) (6.41%), short interspersed nuclear elements (SINEs) (0.04%), and long terminal repeat (LTR) elements (1.20%), 27,348 DNA transposons (0.94%), 7,721 simple repeats (0.12%), and 298 low-complexity repeats (0.01%) were annotated (Fig. 3 and Supplementary Table S3). Repeats are similarly distributed in the Ogye_1.1 and other avian genomes (Fig. 3 and Supplementary Table S4). Compared with other avian genomes, the Ogye_1.1 genome resembles galGal4 and galGal5 the most in terms of repeat composition except for that of simple repeats (0.12% for Ogye_1.1, 1.12% for galGal4, and 1.24% for galGal5), low-complexity (0.01% for Ogye_1.1, 0.24% for galGal4, and 0.25% for galGal5), and satellite DNA repeats (0.01% for Ogye_1.1, 0.20% for galGal4, and 0.22% for galGal5). The distribution of transposable elements across all chromosomes is depicted in Supplementary Fig. S7.

### SNPs and INDELs

To annotate SNPs and INDELs in the Ogye_1.1 genome, all paired-end libraries were mapped to the Ogye_1.1 genome using BWA-MEM and deduplicated using Picard modules [57]. We identified 3,206,794 SNPs and 302,463 INDELs across the genome using VarScan 2 with options –min-coverage 8 –min-reads2 2 –min-avg-qual 15 –min-var-freq 0.2 –p-value 1e-2 [58]. The densities of SNPs and INDELs across all chromosomes are depicted in Supplementary Fig. S7.

### Protein-coding genes

**Figure 4:**
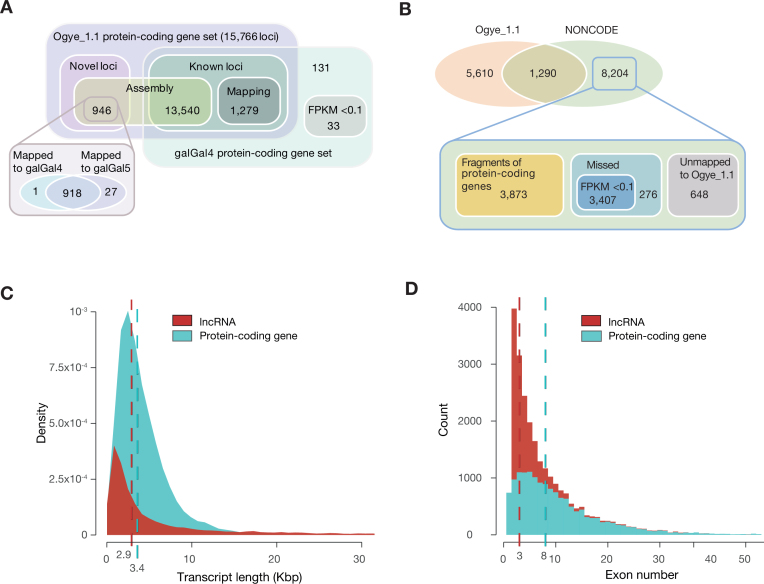
**(A)** A Venn diagram showing the number of protein-coding genes in the Ogye_1.1 genome. (**B)** A Venn diagram showing the number of Ogye_1.1 and galGal4 lncRNAs. (**C)** Distribution of transcript length (red for lncRNAs and cyan for protein-coding genes). The vertical dotted lines indicate the median length. (**D)** Distribution of the number of exons per transcript. Otherwise, as in **(C)**.

To sensitively annotate protein-coding genes, all paired-end RNA-seq data were mapped on the Ogye_1.1 genome using STAR [59] for each tissue, and the mapping results were then assembled into potential transcripts using StringTie [60]. Assembled transcripts from each sample were merged using StringTie, and the resulting transcriptome was subjected to the prediction of coding DNA sequences (CDSs) using TransDecoder [61]. For high-confidence prediction, transcripts with intact gene structures (5’UTR, CDS, and 3’UTR) were selected. To verify their coding potential, the candidate sequences were examined using CPAT [62] and CPC [63]. Candidates with a high CPAT score (>0.99) were directly assigned to be protein-coding genes, and those with an intermediate score (0.8–0.99) were re-examined to determine whether the CPC score was >0. Candidates with low coding potential or that were partially annotated were examined to determine if their loci overlapped with annotated protein-coding genes from galGal4 (ENSEMBL cDNA release 85). Overlapping genes were added to the set of Ogye_1.1 protein-coding genes. Using this protein-coding gene annotation pipeline (Supplementary Fig. S8), 15,766 protein-coding genes were finally annotated in the Ogye_1.1 genome, including 946 novel genes and 14,819 known genes (Fig. 4A). However, 164 galGal4 protein-coding genes were not mapped to the Ogye_1.1 genome by GMAP (Supplementary Table S5), 131 of which were confirmed to be expressed in *YO* (≥0.1 FPKM) using all paired-end *YO* RNA-seq data. In contrast, the remaining 33 genes were not expressed in *YO* (<0.1 FPKM) or were lost from the Ogye_1.1 genome. Of the 33 missing genes, 26 appeared to be located on unknown chromosomes and the remainder are on autosomes (six genes) or the W sex chromosome (one gene) in galGal4. The density of protein-coding genes across all chromosomes is depicted in Supplementary Fig. S7.

### lncRNAs

To annotate and profile lncRNA genes, we used our lncRNA annotation pipeline (Supplementary Fig. S9), adopted from our previous study [64]. Pooled single- and paired-end RNA-seq reads from each tissue were mapped to the Ogye_1.1 genome (PRJNA412424) using STAR [59] and subjected to transcriptome assembly using Cufflinks (Cufflinks, RRID:SCR_014597) [65], leading to the construction of transcriptome maps for 20 tissues. The resulting maps were combined by Cuffmerge and, in total, 206,084 transcripts from 103,405 loci were reconstructed in the Ogye genome. We removed other RNA biotypes (the sequences of mRNAs, tRNAs, rRNAs, snoRNAs, miRNAs, and other small noncoding RNAs downloaded from ENSEMBL biomart) and short transcripts (less than 200 nt in length). A total of 54,760 lncRNA candidate loci (60,257 transcripts) were retained and compared with a chicken lncRNA annotation from NONCODE (v2016) [66]. Of the candidates, 2,094 loci (5,215 transcripts) overlapped with previously annotated chicken lncRNAs. Then, 52,666 nonoverlapping loci (55,042 transcripts) were further examined to determine whether they had coding potential using CPC score [63]. Those with a score greater than –1 were filtered out, and the remainder (14,108 novel lncRNA candidate loci without coding potential) were subjected to the next step. Because many candidates still appeared to be fragmented, those with a single exon but with neighboring candidates within 36,873 bp, which is the length of introns in the 99th percentile, were re-examined using both exon-junction reads consistently presented over 20 tissues and the maximum entropy score [67], as done in our previous study [64]. If there were at least two junction reads spanning two neighboring transcripts or if the entropy score was greater than 4.66 in the interspace, the two candidates were reconnected, and those with a single exon were discarded. In the final version, 6,900 loci (5,610 novel and 1,290 known) were annotated as lncRNAs (see Fig. 4B), which included 6,170 (89.40%) intergenic lncRNAs and 730 (10.57%) anti-sense ncRNAs. Consistent with previous results [68–71], the median Ogye lncRNA transcript length and exon number were less than those of protein-coding genes (Fig. 4C and 4D).

Whereas 13,540 of 14,983 protein-coding genes (90.4%) were redetected in our protein-coding gene annotations (see Fig. 4A), only 1,290 (13.6%) of NONCODE lncRNAs were redetected in our Ogye_1.1 lncRNA annotations (Fig. 4B). The majority of the missing NONCODE lncRNAs were either fragments of protein-coding genes or not expressed in all 20 Ogye tissues (Fig. 4B). Only 276 were actually missing in the transcriptome assembly, and 648 were not mapped to the Ogye_1.1 genome.

## Coding and noncoding transcriptome maps

**Figure 5:**
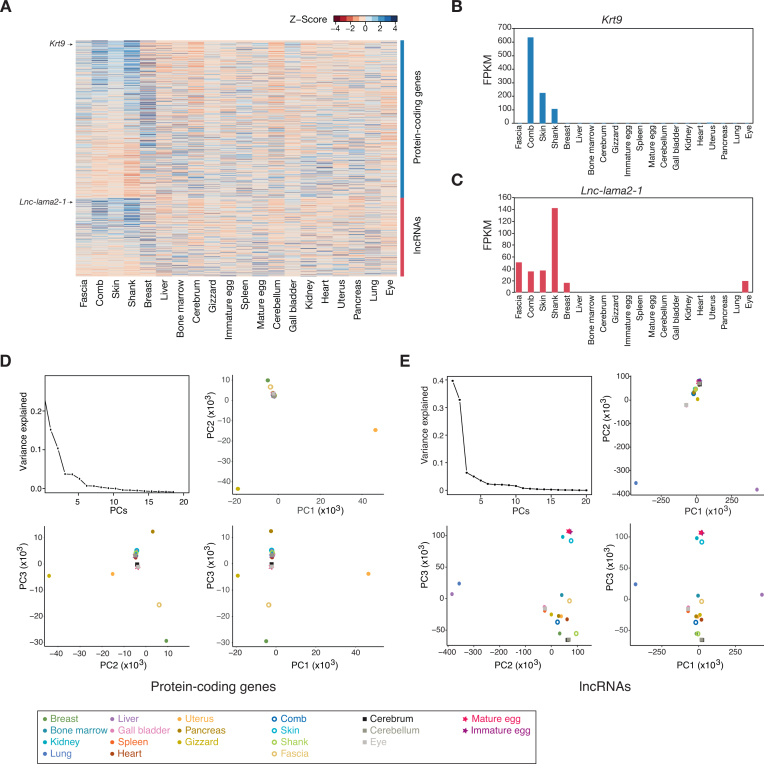
**(A)** The expression patterns of the genes expressed with $ \ge 10\;$ FPKM in black tissues. Expression levels are indicated with a color-coded Z-score (red for low and blue for high expression) as shown in the key. (**B)** Expression levels of *krt9* across 20 tissues. **(C)** Expression levels of *lnc-lama2–1* across 20 tissues. **(D)** Principal component analysis (PCA) using tissue-specific protein-coding genes. PCs explaining the variances are indicated with the amount of the contribution in the top-left plot. PCA plots are shown with PC1, PC2, and PC3 plotted in a pairwise manner. Each tissue is indicated on the PCA plot with a specific color. **(E)** PCA using tissue-specific lncRNAs. Otherwise, as in **(A)**.

Using paired-end *YO* RNA-seq data, the expression levels of protein-coding and lncRNA genes were calculated across 20 tissues (Supplementary Fig. S10). In the profiled transcriptomes, 1,814 protein-coding and 1,226 lncRNA genes were expressed with ≥10 FPKM in only one tissue, whereas 1,559 protein-coding and 351 lncRNA genes were expressed with ≥10 FPKM in all tissues. In black tissues (fascia, comb, skin, and shank), we have found that 6,702 protein-coding and 3,291 lncRNA genes were expressed with ≥10 FPKM, the majority of which appeared to be expressed in a tissue-specific manner (Fig. 5A). For instance, the protein-coding gene *krt9* and the lncRNA *lnc-lama2-1* are highly expressed in black tissues, particularly in comb and shank, respectively (Fig. 5B and 5C).

Because lncRNAs tend to be specifically expressed in a tissue or in related tissues, they could be more useful than protein-coding genes for defining genomic characteristics of tissues. To prove this idea, principle component analyses were performed with 9,153 tissue-specific protein-coding and 5,191 tissue-specific lncRNA genes using the reshape2 R package (Fig. 5D and 5E) [72]. Here, we classified a gene as tissue-specific if the maximum expression value was at least four-fold higher than the mean value over 20 tissues. As expected, the first, second, and third PCs of lncRNAs enabled us to predict the majority of variances and to better discern distantly related tissues and functionally and histologically related tissues (i.e., black tissues and brain tissues) (Fig. 5E) than those of protein-coding genes (Fig. 5C).

## DNA Methylation Maps

**Figure 6:**
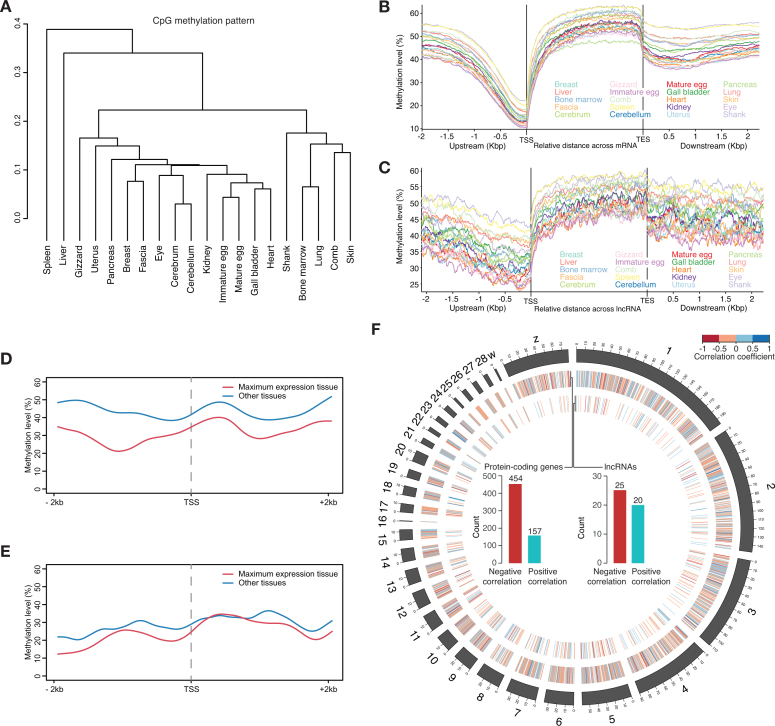
**(A)** Hierarchical clustering using Pearson correlation of DNA methylation patterns between tissues. **(B and C)** Average DNA methylation landscapes along protein-coding **(B)** and lncRNA **(C)** gene bodies and their flanking regions across 20 tissues. **(D and E)** Average DNA methylation levels of protein-coding **(D)** and lncRNA **(E)** genes in the tissue of maximum expression (red) and the other tissues (blue). **(F)** Spearman correlation coefficients between gene expression and promoter methylation levels are shown across chromosomes (heat maps) in a Circos plot. The bar charts indicate the number of genes (left for protein-coding genes and right for lncRNAs) with significant negative (red) and positive (cyan) correlations (*P* < 0.05) between their promoter methylation levels and their expression values.

After mapping RRBS reads to the Ogye_1.1 genome (Table 3), DNA methylation signals (C to T changes in CpGs) were calculated across chromosomes using Bismark [73]. Of all CpG sites in the genome, 31%–65% were methylated across tissues, whereas only 19%–43% were methylated in gene promoters (the region 2 Kbp upstream of the transcription start site [TSS]) (Table 5), indicating that the promoters of expressed genes tended to be hypomethylated. The DNA methylation landscapes in the regions 2 Kbp upstream of the protein-coding and lncRNA gene TSSs are shown in Supplementary Fig. S11. Based on the CpG methylation pattern, hierarchical clustering was performed using the rsgcc R package, and clusters including adjacent or functionally related tissues, such as cerebrum and cerebellum, immature and mature eggs, and comb and skin, were identified (Fig. 6A).

**Table 5:** Summary of methylated CpG sites across 20 tissues

	All genomic region			Promoter region		
		Methylated CpG sites			Methylated CpG sites	
	Total no. of sites	No. of sites	Fraction, %	Total no. of sites	No. of sites	Fraction, %
Breast	994,326	621,751	62.53	228,673	91,704	40.10
Liver	1,641,060	505,775	30.82	522,590	97,597	18.68
Bone marrow	1,096,466	671,781	61.27	254,978	100,385	39.37
Fascia	1,146,350	670,181	58.46	278,618	99,802	35.82
Cerebrum	1,246,514	748,323	60.03	298,677	112,689	37.73
Gizzard	1,024,125	609,010	59.47	234,379	85,273	36.38
Immature egg	1,416,686	809,214	57.12	334,813	115,195	34.41
Comb	1,035,966	642,138	61.98	239,319	92,436	38.62
Spleen	995,639	401,080	40.28	298,833	74,473	24.92
Mature egg	1,144,589	695,258	60.74	269,124	102,282	38.01
Cerebellum	1,279,666	775,513	60.60	305,489	117,950	38.61
Gallbladder	953,630	595,681	62.46	225,122	89,174	39.61
Kidney	1,016,035	610,941	60.13	238,066	89,255	37.49
Heart	1,000,957	611,343	61.08	235,853	90,434	38.34
Uterus	893,101	543,931	60.90	203,102	77,365	38.09
Pancreas	1,119,795	647,577	57.83	267,036	94,371	35.34
Lung	985,824	594,046	60.26	229,316	87,140	38.00
Skin	868,368	565,815	65.16	198,275	85,094	42.92
Eye	1,051,332	663,413	63.10	252,991	105,539	41.72
Shank	862,931	512,853	59.43	210,905	76,512	36.28

We then examined the average methylation landscapes over protein-coding and lncRNA loci to check whether the CpG methylation profiles were properly processed. As previously shown [74–77], the average methylation levels in gene body regions were much higher than those in promoters across tissues (Fig. 6B and 6C). To investigate the association between CpG methylation in the promoter and target gene expression, the average methylation levels of tissue-specific genes (280 protein-coding and 392 lncRNA genes with expression ≥10 FPKM in at least one tissue and with a maximum expression value four-fold higher than the mean expression level in 20 tissues) were compared to those of others expressed in their specific tissues. The methylation levels of highly expressed genes appeared to be lower than those of others (Fig. 6D and 6E). We then searched for genes with tissue-specific expression that was significantly correlated to the promoter methylation level using the Spearman correlation method (Fig. 6F). To exclude stochastic noise, only tissues in which a certain position had a sufficient number of reads (at least five) were taken into account for measuring the correlation. We found that the expression levels of 454 protein-coding and 25 lncRNA genes displayed a negative correlation to promoter methylation levels, whereas 157 protein-coding and 20 lncRNA genes had a positive correlation (box plots in Fig. 6F).

## Discussion

In this work, the first draft genome of *YO*, Ogye_1.1, was constructed with genomic variation, repeat, and protein-coding and noncoding gene maps. Compared with the chicken reference genome maps, many more novel coding and noncoding elements were identified from large-scale RNA-seq datasets across 20 tissues. Although the Ogye_1.1 genome is comparable with galGal5 with respect to genome completeness evaluated using BUSCO, Ogye_1.1 seems to lack simple and long repeats compared with galGal5, which was assembled from high-depth PacBio long reads (50X) that can capture simple and long repeats. Although PacBio long reads were also produced in our study, they were only used for scaffolding and gap-filling because of their shallow depth (9.7X), probably resulting in some simple and satellite repeats being missed in Ogye_1.1. A similar tendency can be seen in the golden-collared manakin genome (ASM171598v1) [32] (Fig. 3) and the gray mouse lemur genome (Mmur3.0) [78], which were also assembled in a hybrid manner with high-depth Illumina short reads and low-depth PacBio long reads.

A total of 15,766 protein-coding and 6,900 lncRNA genes were annotated from 20 *YO* tissues. Also, 946 novel protein-coding genes were identified, while 164 *Galllus gallus red junglefowl* genes were missed in our annotations. In the case of lncRNAs, only about 13.6% of previously annotated chicken lncRNAs were redetected, and the remainder were mostly not expressed in *YO* or were false annotations, suggesting that the current chicken lncRNA annotations should be carefully examined. Our Ogye lncRNAs resembled previously annotated mammalian lncRNAs in their genomic characteristics, including transcript length, exon number, and tissue-specific expression patterns, providing evidence for the accuracy of the new annotations. Hence, our lncRNA catalogue may help us improve lncRNA annotations in the chicken reference genome.

## Availability of supporting data

All of our sequence data and the genome sequence have been deposited in National Center for Biotechnology Information's Gene Expression Omnibus superseries GSE 104 358 and BioProject PRJNA412408. All supporting data (genome and gene sequence files, the expression tables for protein-coding and lncRNA genes, and the RRBS, protein-coding, lncRNA, SNP, and INDEL annotation files) are available in the *GigaScience* repository *Giga*DB [79].

## Additional files

**Additional file 1:** Supplementary Figures and Tables.

**Additional file 2:** Description (README) of available data in *Giga*DB.

**Additional file 3:** Command lines of programs and pipelines with run-time options used in this study.

**Figure S1:** Ogye_1.1 genome assembly statistics at each step.

**Figure S2: A**. An example of mis-assemblies in a scaffold. The x-axis represents the positions on chr1 or chr2 in galGal4 and the y-axis represents the position in scaffold_22 of the scaffold at the second step of the second stage (i.e., Opera scaffolder's result); **B**. In this example, there are two translocations: at P1 between L1 and L_2 and at P2 between L2_and L3. Since L_1, L_2 and L_3 are all >1Mbp, we broke the scaffold at P1 and P2. In this manner, we found 30 break points over all scaffolds in the breaking step of the second stage in Fig. 1B and Fig. S1.

**Figure S3:** Pseudo-reference-assisted assembly pipeline utilizing a hierarchical bipartite graph of PacBio long reads, scaffolds, and galGal4 chromosomes. The tools, used in grouping PacBio reads and scaffolds, are available in https://github.com/sohnjangil/tsrator.git.

**Figure S4:** Alignment of the Ogye_1.1 genome to galGal4/5 drawn by MUMmer.

**Figure S5**: Structural variation (SV) map of the Ogye_1.1 genome compared with galGal4 and galGal5. Insertions (red), deletions (blue), duplications (yellow), inversions (green), inter-chromosomal translocations (gray; Inter-translocation), and intra-chromosomal translocations (orange; Intra-translocation) are shown. SVs between the Ogye_1.1 genome and galGal4 or 5 are shown with Venn diagrams.

**Figure S6:** Mapping positions of mate-pair reads in the *FM* locus. The x- and y-axes indicate the positions of the first- and second-fragments, respectively, of a mate-pair read (insert size 3–10Kbp). The distance between the positions is the insert size of a mate-pair read.

**Figure S7:** Gene (protein-coding and lncRNA) annotation maps of the Ogye_1.1 genome with TE, SNV/INDEL, and GC ratio landscapes shown in a Circos plot. Color codes indicate coverage (%) of TE in a Mbp window, the number of protein-coding genes in a Mbp window, the number of lncRNAs in a Mbp window, SNP and INDEL frequencies in a 100Kbp window, and the GC ratio in a 100Kbp window.

**Figure S8:** A schematic flow of our protein-coding gene annotation pipeline.

**Figure S9:** A computational pipeline for lncRNA annotations.

**Figure S10:** Circos plots illustrating the expression levels of protein-coding genes (bottom) and lncRNAs (top) across twenty tissues. The expression levels are indicated with a color-coded Z-score, described in the key.

**Figure S11:** Circos plots illustrating the CpG methylation levels in the promoters of protein-coding genes (bottom) and lncRNAs (top) across twenty tissues. The methylation levels are indicated with a color-coded Z-score, described in the key.

**Table S1:** Statistics of whole genome sequencing data (Illumina) after quality control.

**Table S2:** Structural variations in the Ogye_1.1 genome.

**Table S3:** Repeats in the Ogye_1.1 genome.

**Table S4:** Repeat composition in different assemblies.

**Table S5:** 164 galGal4 protein-coding genes missed in the Ogye_1.1 protein-coding gene annotations.

## Abbreviations

BUSCO: Benchmarking Universal Single-Copy Orthologs; FM: fibromelanosis; CDS: coding DNA sequence; INDEL: insertions and deletions; PacBio: Pacific Biosciences; PC: principle component; PCA: principle component analyses; PCR: polymerase chain reaction; RNA-seq: RNA sequencing; RRBS: reduced representation bisulfite sequencing; SNP: single nucleotide polymorphism; SV: structural variation; TSS: transcription start site.

## Competing interests

The authors declare that they have no competing interests.

## Funding

This work was supported by the Cooperative Research Program for Agriculture Science and Technology Development (project title: National Agricultural Genome Program, Project No. PJ01045301 and PJ01045303).

## Author contributions

K.T.L., N.S.K., H.H.C., and J.W.N. designed the study. K.T.L., Y.J.D., and C.Y.C. collected samples. D.J.L., H.H.C., and K.T.L. collected sequencing data. J.I.S., K.W.N., N.S.K., J.M.K., H.H.C., and J.M.N. performed the analysis and developed the methodology. J.I.S., K.W.N., J.M.K., H.S.H., and J.W.N. wrote the manuscript.

## Supplementary Material

GIGA-D-17-00321_Original_Submission.pdf

GIGA-D-17-00321_Revision_1.pdf

Response_to_Reviewer_Comments_Original_Submission.pdf

Reviewer_1_Report_(Original_Submission) -- Robert Kraus 02-14-2018 Reviewed

Reviewer_2_Report_(Original_Submission) -- William Chow 2/20/2018 Reviewed

Supplemental Files
