# Supplementary material for: Whole genome and transcriptome maps of the entirely black native Korean chicken breed Yeonsan Ogye
Source: Gigascience. 2018 Jul 11;7(7):giy086. doi: 10.1093/gigascience/giy086 (PMC6065499; doi:10.1093/gigascience/giy086)
Supplement: GIGA-D-17-00321_Original_Submission.pdf [file giy086_giga-d-17-00321_original_submission.pdf]

GigaScience

Whole genome and transcriptome maps of the entirely black native Korean chicken breed Yeonsan Ogye

--Manuscript Draft--

Manuscript Number:	GIGA-D-17-00321
Full Title:	Whole genome and transcriptome maps of the entirely black native Korean chicken breed Yeonsan Ogye
Article Type:	Data Note
Funding Information:	Rural Development Administration (PJ01045303) — Prof Jin-Wu Nam<br>Rural Development Administration (PJ01045301) — Ms. Han-Ha Chai
Abstract:	Yeonsan Ogye (YO), an indigenous Korean chicken breed (gallus gallus domesticus), has entirely black external features and internal organs. In this study, the draft genome of YO was assembled using a hybrid de novo assembly method that takes advantage of high-depth Illumina short reads (232.2X) and low-depth PacBio long reads (11.5X). The contig and scaffold N50s (defined as the shortest contig or scaffold length at 50% of the entire assembly) of the hybrid de novo assembly were 504.8Kbp and 21.2Mbp, respectively. The completeness (97.6%) of the draft genome (Ogye_1.1) was evaluated with single copy orthologous genes using BUSCO, and found to be comparable to the current chicken reference genome (galGal5; 97.4%; assembled with a long read-only method), and superior to other avian genomes (92~93%; assembled with short read-only or hybrid methods). Compared to galGal4 and 5, the draft genome included 551 structural variations including the Fibromelanosis (FM) locus duplication, related to hyperpigmentation. To comprehensively reconstruct transcriptome maps, RNA sequencing (RNA-seq) and reduced representation bisulfite sequencing (RRBS) data were analyzed from twenty different tissues, including four black tissues (skin, shank, comb, and fascia). The maps included 15,766 protein-coding and 6,900 long non-coding RNA genes, many of which were tissue-specifically expressed and displayed tissue-specific DNA methylation pattern in the promoter regions. We expect that the resulting genome sequence and transcriptome maps will be valuable resources for studying domestic breeds of chickens, including black-skinned chickens, as well as for understanding genomic differences and evolution of hyperpigmented chickens and functional elements related to hyperpigmentation.
Corresponding Author:	Jin-Wu Nam<br>Hanyang University<br>Seoul, KOREA, REPUBLIC OF
Corresponding Author Secondary Information:	
Corresponding Author's Institution:	Hanyang University
Corresponding Author's Secondary Institution:	
First Author:	Jang-il Sohn, Ph.D.
First Author Secondary Information:	
Order of Authors:	Jang-il Sohn, Ph.D.
	Kyoungwoo Nam
	Hyosun Hong
	Jun-Mo Kim, Ph.D.
	Dajeong Lim, Ph.D.
	Kyung-Tai Lee, Ph.D.
	Yoon Jung Do, Ph.D.

Powered by Editorial Manager® and ProduXion Manager® from Aries Systems Corporation

	Chang Yeon Cho, Ph.D.
	NamShin Kim, Ph.D.
	Jin-Wu Nam, Ph.D.
	Han-Ha Chai
**Order of Authors Secondary Information:**	
**Opposed Reviewers:**	
**Additional Information:**	
**Question**	**Response**
Are you submitting this manuscript to a special series or article collection?	No
**Experimental design and statistics**<br><br>Full details of the experimental design and statistical methods used should be given in the Methods section, as detailed in our Minimum Standards Reporting Checklist. Information essential to interpreting the data presented should be made available in the figure legends.<br><br>Have you included all the information requested in your manuscript?	Yes
**Resources**<br><br>A description of all resources used, including antibodies, cell lines, animals and software tools, with enough information to allow them to be uniquely identified, should be included in the Methods section. Authors are strongly encouraged to cite Research Resource Identifiers (RRIDs) for antibodies, model organisms and tools, where possible.<br><br>Have you included the information requested as detailed in our Minimum Standards Reporting Checklist?	Yes
**Availability of data and materials**<br><br>All datasets and code on which the conclusions of the paper rely must be either included in your submission or deposited in publicly available repositories (where available and ethically appropriate), referencing such data using a unique identifier in the references and in the "Availability of Data and Materials" section of your manuscript.	Yes

Have you have met the above requirement as detailed in our Minimum Standards Reporting Checklist?	

Powered by Editorial Manager® and ProduXion Manager® from Aries Systems Corporation

Manuscript
Click here to download Manuscript Ogye_GigaScience_20180110_Main.docx

DATA NOTE

Whole genome and transcriptome maps of the entirely black native Korean chicken breed *Yeonsan Ogye*

Jang-il Sohn[1,2*], Kyoungwoo Nam[1,*], Hyosun Hong[1,*], Jun-Mo Kim[3,*], Dajeong Lim[3], Kyung-Tai Lee[3], Yoon Jung Do[3], Chang Yeon Cho[4], Namshin Kim[5], Han-Ha Chai[3,§] and Jin-Wu Nam[1,2,‡]

[1]Department of Life Science, Hanyang University, Seoul 133-791,
[2]Research Institute for Convergence of Basic Sciences, Hanyang University, Seoul 133-791,
[3]Department of Animal Biotechnology & Environment, National Institute of Animal Science, RDA, Wanju 55365,
[4]Animal Genetic Resource Research Center, National Institute of Animal Science, RDA, Namwon 55717,
[5]Personalized Genomic Medicine Research Center, KRIBB, Daejeon 34141, Republic of Korea

* These authors contributed equally to this paper
§ hanha@korea.kr
‡ jwnam@hanyang.ac.kr

Abstract

Yeonsan Ogye (*YO*), an indigenous Korean chicken breed (*gallus gallus domesticus*), has entirely black external features and internal organs. In this study, the draft genome of *YO* was assembled using a hybrid *de novo* assembly method that takes advantage of high-depth Illumina short reads (232.2X) and low-depth PacBio long reads (11.5X). The contig and scaffold N50s (defined as the shortest contig or scaffold length at 50% of the entire assembly) of the hybrid *de novo* assembly were 504.8Kbp and 21.2Mbp, respectively. The completeness (97.6%) of the draft genome (Ogye_1.1) was evaluated with single copy orthologous genes using BUSCO, and found to be comparable to the current chicken reference genome (galGal5; 97.4%; assembled with a long read-only method), and superior to other avian genomes (92~93%; assembled with short read-only or hybrid methods). Compared to galGal4 and 5, the draft genome included 551 structural variations including the Fibromelanosis (*FM*) locus duplication, related to hyperpigmentation. To comprehensively reconstruct transcriptome maps, RNA sequencing (RNA-seq) and reduced representation bisulfite sequencing (RRBS) data were analyzed from twenty different tissues, including four black tissues (skin, shank, comb, and fascia). The maps included 15,766 protein-coding and 6,900 long non-coding RNA genes, many of which were tissue-specifically expressed and displayed tissue-specific DNA methylation pattern in the promoter regions. We expect that the resulting genome sequence and transcriptome maps will be valuable resources for studying domestic breeds of chickens, including black-

skinned chickens, as well as for understanding genomic differences and evolution of hyperpigmented chickens and functional elements related to hyperpigmentation.

Keywords: *Gallus gallus domesticus*; *Yeonsan Ogye*; whole genome *de novo* assembly; Transcriptome maps; Hyperpigmentation

Background

The *Yeonsan Ogye* (*YO*), a designated natural monument of Korea (No. 265), is an indigenous Korean chicken breed that is notable for its entirely black plumage, skin, beak, comb, eyes, shank, claws, and internal organs [1]. In terms of its plumage and body color, as well as its number of toes, this unique chicken breed resembles the indigenous Indonesian chicken breed *Ayam cemani* [2-4]. *YO* also has some morphological features that are similar to those of the *Silkie* fowl, except for a veiled black walnut comb and hair-like, fluffy plumage that is white or variably colored [5, 6]. Although the exact origin of the *YO* breed has not yet been clearly defined, its features and medicinal usages were recorded in *Dongui Bogam* [7], a traditional Korean medical encyclopedia compiled and edited by Heo Jun in 1613.

To date, a number of avian genomes from both domestic and wild species have been constructed and compared, revealing genomic signatures associated with the domestication process and genomic differences that provide an evolutionary perspective [8]. The chicken reference genome was first assembled using the *Red junglefowl* [9], first domesticated at least five thousand years ago in Asia; the latest version of the reference genome was released in 2015 (galGal5, GenBank Assembly ID GCA_000002315.3) [10]. However, because domesticated chickens exhibit diverse morphological features, including skin and plumage colors, the genome sequences of unique breeds are necessary for understanding their characteristic phenotypes through analyses of single nucleotide polymorphisms (SNPs), insertions and deletions (INDELs), structural variations (SVs), and coding and non-coding transcriptomes. Here, we provide the first version of *YO* genome (Ogye_1.1), which include annotations of large SVs, SNPs, INDELs, and repeats, as well as coding and non-coding transcriptome maps along with DNA methylation landscapes across twenty different tissues of *YO*.

Results

Sample collection and data description

8-month-old *YO* chickens (object number: 02127), obtained from the Animal Genetic Resource Research Center of the National Institute of Animal Science (Namwon, Korea), were used in the study (**Figure 1A**). The protocols for the care and experimental use of *YO* were reviewed and approved by the Institutional Animal Care and Use Committee of the National Institute of Animal Science (IACUC No.: 2014-080). *YO* management, treatment, and sample collection took place at the National Institute of Animal Science.

Whole genome sequencing

Genomic DNA was extracted from blood using Wizard DNA extraction kit [11] and prepared for DNA sequencing library construction. According to the DNA fragment (insert) size, three different library types were constructed: paired-end library for small inserts (280 and 500 bp) and mate-pair library for large inserts (3, 5, 8, and 10 Kbp), and fosmid libraries (40 Kbp) using Illumina's protocols (Illumina, San Diego, CA, USA) (**Table 1**). The constructed libraries were sequenced using Illumina's Hiseq2000 platform. In total, 232.2 X Illumina short reads were obtained (59.6 X from the small insert libraries and 172.6 X from the large insert libraries) and, after filtering raw data with low quality (> 30% of the base-pairs in a read have a Phred score <20), 163.5X were used for genome assembly. To fill gaps and improve the scaffold quality, 11.5X PacBio long reads were additionally sequenced; the average length of the long reads was 6Kbp (**Table 1**).

Whole transcriptome sequencing

Total RNAs were extracted from twenty different tissues using 80% EtOH and TRIzol. The RNA concentration was checked by Quant-IT RiboGreen (Invitrogen, Carlsbad, USA). To assess the integrity of the total RNA, samples were run on a TapeStation RNA screentape (Agilent, Waldbronn, Germany). Only high quality RNA samples (RIN c7.0) were used for RNA-seq library construction. Each library was independently prepared with 300ng of total RNA using an Illumina TruSeq Stranded Total RNA Sample

Prep Kit (Illumina, San Diego, CA, USA). The rRNA in the total RNA was depleted using a Ribo-Zero kit. After rRNA depletion, the remaining RNA was purified, fragmented and primed for cDNA synthesis. The cleaved RNA fragments were copied into the first cDNA strand using reverse transcriptase and random hexamers. This step was followed by second strand cDNA synthesis using DNA Polymerase I, RNase H and dUTP. The resulting cDNA fragments then underwent an end repair process, the addition of a single 'A' base, after which adapters were ligated. The products were purified and enriched with PCR to create the final cDNA library. The libraries were quantified using qPCR according to the qPCR Quantification Protocol Guide (KAPA Library Quantificatoin kits for Illumina Sequecing platforms) and qualified using the TapeStation D1000 ScreenTape assay (Agilent Technologies, Waldbronn, Germany). As a result, about 1.5 billion RNA-seq reads were sequenced from twenty different tissues, which are breast, liver, bone marrow, fascia, cerebrum, gizzard, mature and immature eggs, comb, spleen, cerebellum, gall bladder, kidney, heart, uterus, pancreas, lung, skin, eye, and shank (**Table 2**).

Reduced representation bisulfite sequencing

Preparation of reduced representation bisulfite sequencing (RRBS) libraries was done following Illumina's RRBS protocol. 5μg of genomic DNA that had been digested with the restriction enzyme MspI and purified with a QIAquick PCR purification kit (QIAGEN, Hilden, Germany) was used for library preparation, which was done using a TruSeq Nano DNA Library Prep Kit (Illumina, San Diego, USA). Eluted DNA fragments were end-repaired, extended on the 3' end with an 'A', and ligated with Truseq adapters. After ligation had been assessed, the products, which ranged from 175 to 225bp in length (insert DNA of 55–105 bp plus adaptors of 120 bp), were excised from a 2%(w/v) Low Range Ultra Agarose gel (Biorad, Hercules, USA) and purified using the QIAquick gel extraction protocol. The purified DNA underwent bisulfite conversion using an EpiTect Bisulfite Kit (Qiagen, 59104). The bisulfite-converted DNA libraries were amplified by PCR (four cycles) using PfuTurbo Cx DNA polymerase (Agilent, 600410). The final product was then quantified using qPCR and qualified using the Agilent Technologies 2200 TapeStation assay (Agilent, Waldbronn, Germany). The final product was sequenced using the HiSeq™

2500 platform (Illumina, San Diego, USA). As a result, 123 million RRBS reads were produced from twenty different tissues (see **Table 3**).

Hybrid whole genome assembly

The Ogye_1.1 genome was assembled using our hybrid genome assembly pipeline, employing the following four steps: preprocessing, hybrid *de novo* assembly, super-scaffolding, and polishing (**Figure 1B** and **Figure S1A).** During the preprocessing step, the errors in the Illumina short reads were corrected by KmerFreq and Corrector [12]. In turn, using the corrected short reads, the sequencing errors in the PacBio long reads were corrected by LoRDEC [13].

In hybrid *de novo* genome assembly, the initial assembly was done with the error-corrected short reads from the paired-end and mate-pair libraries using ALLPATHS-LG [14] with the default option, producing contigs and scaffolds of 53.6 Kbp and 10.7 Mbp N50 lengths, respectively (**Figure 1B; S1B**). Next, the scaffolds were additionally connected with corrected PacBio long reads and FOSMID reads using SSPACE-LongRead [15] and OPERA [16]. The gaps within and between scaffolds were re-examined with GapCloser [12] with error-corrected short reads. All resulting scaffolds were aligned to the galGal4 genome (GenBank assembly accession: GCA_000002315.2) by LASTZ [17]. Analyzing the resulting alignments, 30 mis-assemblies with large translocations or inversions of >1Mbp were detected and were broken at each break point, as described in **Figure S2**. Breaking scaffolds at the break points resulted in pseudo-contig N50 of 108.6 Kbp and scaffold N50 of 18.7 Mb (**Figure S1B**). A pseudo-contig is defined by a sequence broken by gaps of >1bp, which are assumed to be gaps or errors.

In the super-scaffolding stage, pseudo-reference-assisted assembly was done with error-corrected PacBio long reads using LASTZ, BWA-MEM, PBJelly [18], and SSPACE-LongRead, which could lead to reducing the topological complexity of the assembly graphs [19] (**Figure 1B**). Because even scaffolding with long reads can be affected by repetitive sequences, the scaffolds mapped to each chromosome were transformed into a hierarchical bipartite graph to minimize the influence of repetitive sequences (**Figure**

S3). To build the hierarchical bipartite graph, PacBio (error-corrected) reads were mapped to the scaffolds using BWA-MEM again and, in turn, the scaffolds were mapped to the galGal4 genome using LASTZ. Using the hierarchical bipartite graphs, all scaffolds and PacBio reads were finally assigned to each chromosome. Based on the results, super-scaffolding and additional gap-filling was performed by SSPACE-LongRead and PBJelly, respectively, resulting in scaffold N50 of 21.2Mbp (**Figure 1C**).

In the last polishing stage, nucleotide errors or ambiguities were corrected by the GATK pipeline [20] with paired-end reads, and in turn, any vector contamination was removed using VecScreen with UniVec database [21] (**Figure 1B**). The final assembly results showed that the gap percentage and (pseudo-)contig N50 were significantly improved, from 1.87% and 53.6 Kbp in the initial assembly to 0.85% and 504.8 Kbp in the final assembly, respectively (**Figure S1B**). Among avian genome assemblies, these results are second best and the scaffold N50 is the best (**Figure 1C**). The complete genome sequence at the chromosome level was built by connecting final scaffolds in the order of appearance in each chromosome with the introduction of 100 Kbp 'N' gaps between them (**Figure S4**) (see *Giga*DB). To evaluate the completeness of the genome, the Ogye_1.1 genome was compared to the galGal4 (short read-based assembly) and galGal5 (long read-based assembly) genomes, with respect to 2,586 conserved vertebrate genes, using BUSCO [22]. The Ogye_1.1 genome contained more complete single-copy BUSCO genes (**Table 4**).

Large structural variations

When the Ogye_1.1 genome was compared to galGal4 and 5 using LASTZ [17], putative large structural variations (>1 Kbp) were detected for each reference genome, and they were validated by at least a different SV prediction programs (Delly, Lumpy, FermiKit, and novoBreak) [23-26] (**Figure 2A**; **Table S1**). SVs included 185 deletions (DELs), 180 insertions (INSs), 158 duplications (DUPs), 23 inversions (INVs), and 5 intra or inter-chromosomal translocations (TRAs). 290 and 447 distinct SVs were detected relative to galGal 4 and galGal5, respectively (**Figure 2A**), suggesting that two reference assemblies could include mis-assemblies.

Although the Fibromelanosis (*FM*) locus, which contains the hyperpigmentation-related *edn3* gene, is known to be duplicated in the genomes of certain hyperpigmented chicken breeds, such as *Silkie* and *Ayam cemani* [3, 6] , the exact structure of the duplicated *FM* locus in such breeds has not been completely resolved due to its large size (~1Mbp). A previous study suggested that the inverted duplication of the *FM* locus could be explained by three possible mechanistic scenarios (**Figure 2B**) [3]. To understand more about the mechanism of *FM* locus SV in the Ogye_1.1 genome, the *YO*'s *FM* locus was compared to that of galGal4 with paired-end reads aligned to the galGal4 genome. Higher read depth at the *FM* locus in *YO* was detected, indicating a copy gain of the locus (**Figure 2C** top). As previously reported [3, 6], our paired-end and mate-pair reads of *YO*'s *FM* locus were discordantly mapped to the galGal4 *FM* locus (**Figure S5**). The intervening region between the two duplicated regions was estimated at 412.6 Kbp in length in Ogye_1.1. Regarding possible mechanistic scenarios, mate-pair reads (3-10 Kbp and FOSMID) mapped to the locus supported all three mechanistic scenarios, but an alignment between Ogye_1.1 and galGal4 chromosome 20 showed that the intervening regions, including inner-partial regions in both duplicated regions, were inverted at the same time (**Figure 2C**), which could support the first mechanistic scenario in **Figure 2B**. Given the resulting alignments, the *FM* locus of the Ogye_1.1 genome was updated according to the first scenario. The size of Gap_1 and Gap_2 were estimated at 164.5 Kbp and 63.3 Kbp, respectively.

Annotations

Repeats

Repeat elements in the Ogye_1.1 and other genomes were predicted by a reference-guided approach using RepeatMasker [27] with Repbase libraries [28]. In the Ogye_1.1 genome, 205,684 retro-transposable elements (7.65%), including long interspersed nuclear elements (LINEs; 6.41%), short interspersed nuclear elements (SINEs; 0.04%) and long terminal repeat (LTR) elements (1.20%), 27,348 DNA transposons (0.94%), 7,721 simple repeats (0.12%), and 298 low-complexity repeats (0.01%) were annotated (**Figure 3** and **Table S2**). Repeats in the Ogye_1.1 genome are similarly distributed with those of other avian genomes (**Figure 3** and **Table S3**). Compared with other avian genomes, the Ogye_1.1 genome resembles

to galGal4 and 5 in terms of repeat composition except for simple repeats (0.12% for Ogye_1.1, 1.12% for galGal4 and 1.24% for galGal5), low-complexity (0.01% for Ogye_1.1, 0.24% for galGal4 and 0.25% for galGal5) and satellite DNA repeats (0.01% for Ogye_1.1, 0.20% for galGal4 and 0.22% for galGal5). The coverage of transposable elements (TEs) across all chromosomes was depicted in **Figure 4A**.

SNPs/INDELs

To annotate SNPs and INDELs in the Ogye_1.1 genome, all paired-end libraries were mapped to the Ogye_1.1 genome using BWA-MEM and deduplicated by Picard modules [29]. As a result, 599,326 SNPs and 23,274 insertions/deletions (INDELs) were identified across the genome using Genome Analysis Toolkit (GATK) modules: HaplotypeCaller, combineGVCF, GenotypeGVCFs and VariantFiltration (with options "`QD < 2.0 || FS > 200.0 || ReadPosRankSum < -20.0`") [20]. The densities of SNPs and INDELs across all chromosomes are depicted in **Figure 4A**.

Protein-coding genes

To sensitively annotate protein-coding genes, all paired-end RNA-seq data were mapped on the Ogye_1.1 genome by STAR [30] for each tissue and the mapping results were then assembled into potential transcripts using StringTie [31]. Assembled transcripts from each sample were merged using StringTie and the resulting transcriptome was subjected to the prediction of coding DNA sequences (CDSs) using TransDecoder [32]. For high-confidence prediction, transcripts with intact gene structures (5'UTR, CDS, and 3'UTR) were selected. To verify the coding potential, the candidate sequences were examined using CPAT [33] and CPC [34]. Candidates with a high CPAT score (>0.99) were directly assigned to be protein-coding genes, and those with an intermediate score (0.8-0.99) were re-examined to determine whether the CPC score is >0. Candidates with low coding potential or that were partially annotated were examined to determine if their loci overlapped with annotated protein-coding genes from galGal4 (ENSEMBL cDNA release 85). Overlapping genes were added to the set of Ogye_1.1 protein-coding genes. Using this protein-coding gene annotation pipeline (**Figure 4B**), 15,766 protein-coding genes were finally annotated in the Ogye_1.1 genome, including 946 novel genes and 14,819 known genes (**Figure 4C**). In addition, 164

galGal4 protein-coding genes were not mapped to the Ogye_1.1 genome by GMAP (**Table S4**), 131 of which were confirmed to be expressed in *YO* ($\geq$ 0.1 FPKM) using all paired-end *YO* RNA-seq data. However, expression of the remaining 33 genes was not confirmed, suggesting that they are not expressed in *YO* (< 0.1 FPKM) or have been lost from the Ogye_1.1 genome. The density of protein-coding genes across all chromosomes was depicted in **Figure 4A**.

lncRNAs

To annotated and profile lncRNA genes, we performed our lncRNA annotation pipeline (**Figure 5A**), adopted from our previous study [35]. Pooled single- and paired-end RNA-seq reads of each tissue were mapped to the Ogye_1.1 genome (PRJNA412424) using STAR [30], and subjected to transcriptome assembly using Cufflinks [36], leading to the construction of transcriptome maps for twenty tissues. The resulting maps were combined by Cuffmerge and, in total, 206,084 transcripts from 103,405 loci were reconstructed in the Ogye genome. We removed other biotypes of RNAs (the sequences of mRNAs, tRNAs, rRNAs, snoRNAs, miRNAs, and other small non-coding RNAs downloaded from ENSEMBL biomart) and short transcripts (less than 200nt in length). 54,760 lncRNA candidate loci (60,257 transcripts) were retained, and which were compared with a chicken lncRNA annotation of NONCODE (v2016) [37]. Of the candidates, 2,094 loci (5,215 transcripts) overlapped with previously annotated chicken lncRNAs. 52,666 non-overlapping loci (55,042 transcripts) were further examined to determine whether they had coding potential using CPC score [34]. Those with a score greater than -1 were filtered out, and the remainder (14,108 novel lncRNA candidate loci without coding potential) were subjected to the next step. Because many candidates still appeared to be fragmented, those with a single exon but with neighboring candidates within 36,873bp, which is the intron length of the 99th percentile, were re-examined using both exon-junction reads consistently presented over twenty tissues and the maximum entropy score [38], as done in our previous study [35]. If there were at least two junction reads spanning two neighboring transcripts or if the entropy score was greater than 4.66 in the interspace, two candidates were reconnected, and those with a single exon were discarded. In the final version, 6,900 lncRNA loci (5,610 novel and 1,290 known) were

annotated as lncRNAs (see **Figure 5B**), which included 6,170 (89.40 %) intergenic lncRNAs and 730 (10.57 %) anti-sense ncRNAs. Consistently with other species [39-42], the median transcript length and the median exon number of Ogye lncRNAs were less than those of protein-coding genes (**Figure 5C** and **D**).

Whereas 13,540 of 14,983 protein-coding genes (90.4%) were redetected in our protein-coding gene annotations (see **Figure 4C**), only 1,290 (13.6%) of NONCODE lncRNAs were redetected in our lncRNA annotations of Ogye_1.1 (**Figure 5B**). The majority of the missed NONCODE lncRNAs were either fragments of protein-coding genes or not expressed in all twenty Ogye tissues (**Figure 5B**). Only 276 were actually missed in the transcriptome assembly and 648 were not mapped to Ogye_1.1 genome.

Coding and non-coding transcriptome maps

Using paired-end *YO* RNA-seq data, the expression levels of protein-coding and lncRNA genes were calculated across twenty tissues (**Figure 6A**), which were dynamically changed. In the profiled transcriptomes, 1,814 protein-coding genes and 1,226 lncRNA genes were expressed with ≥ 10 FPKM in only one tissue, while 1,559 and 351 genes were expressed with ≥ 10 FPKM in all tissues, respectively. In black tissues (fascia, comb, skin, and shank), we have found that 6,702 protein-coding and 3,291 lncRNA genes were expressed with ≥ 10 FPKM, and the majority of which appeared to be expressed in tissue-specific manner (**Figure 6B**). For instance, a protein-coding gene, *krt9*, and, an lncRNA, *lnc-lama2-1* are highly expressed in black tissues including comb and shank, respectively (**Figure 6C** and **D**).

As lncRNAs tend to be specifically expressed in a tissue or in related tissues, they could be better factors for defining genomic characteristics of tissues than protein-coding genes. To prove this idea, principle component analyses (PCA) were performed with tissue-specific 9,153 protein-coding and 5,191 lncRNA genes using reshape2 R package (**Figure 7**) [43]. Here, we called a gene tissue-specific, if the maximum expression value is four-fold higher than mean value over twenty tissues. As expected, the 1st, 2nd, and 3rd PCs of lncRNAs enabled us to predict the majority of variances, and better discerned distantly-related tissues and functionally and histologically-related tissues (*i.e.*, black tissues and brain tissues)

(**Figure 7B**) than those of protein-coding genes (**Figure 7A**).

DNA methylation maps

After mapping RRBS reads to Ogye_1.1 genome (**Table 3**), DNA methylation signals (C to T changes in CpGs) were calculated across chromosomes using Bismark [44]. Of all CpG sites in genomes, 31~65% were methylated across tissues while only 19~43% were methylated in the promoter (2 Kbp upstream region from transcription start site (TSS)) of genes (**Table 5**), indicating hypomethylation status in the promoter of expressed genes. The DNA methylation landscape in the 2 Kbp upstream region from transcription start site (TSS) of protein-coding and lncRNA genes were shown in **Figure 8A**. Based on the CpG methylation pattern, hierarchical clustering was performed using rsgcc R package, and clusters including adjacent or functionally related tissues, such as cerebrum and cerebellum, immature and mature eggs, or comb and skin were identified (**Figure 8B**).

We, then, examined the average methylation landscapes over protein-coding and lncRNA loci to check whether the CpG methylation profiles were properly processed. As previously shown [45-48]; the average methylation levels in gene body regions were much higher than those in the promoters across tissues (**Figure 8C** and **D**). To investigate the association of the CpG methylation in the promoter and the target gene expression, the average methylation levels of tissue specifically expressed genes (280 for protein-coding and 392 for lncRNA genes with 10 FPKM in at least only tissue and with the four-fold higher maximum expression value than the mean value across 20 tissues) were compared to those of others in their specific tissues. The methylation levels of highly expressed genes appeared to be lower than those of others (**Figure 8E** and **F**). The tissue-specific genes with a significant correlation to the methylation levels in the promoters were, then, searched using the Spearman's correlation method (**Figure 9**). To exclude stochastic noises, only tissues in which a certain position had sufficient read coverage (at least five) were taken account for measuring the correlation. As a result, 454 protein-coding and 25 lncRNA genes displayed a negative correlation to methylation levels in promoter regions, while 157 protein-coding and 20 lncRNA genes have a positive correlation (box plots in **Figure 9**).

Discussions

In this work, the first draft genome of *YO*, Ogye_1.1, was constructed with genomic variation, repeat, and protein-coding and non-coding gene maps. Compared with the chicken reference genome maps, many more novel coding and non-coding elements were identified from large-scale RNA-seq datasets across twenty different tissues. Although the Ogye_1.1 genome is comparable with galGal5 with respect to genome completeness evaluated by BUSCO, the Ogye_1.1 seems to lack simple and long repeats compared with galGal5, assembled from high-depth PacBio long reads (50X) that can capture simple and long repeats. Although PacBio long reads were also produced in our study, they were only used for scaffolding and gap-filling because of its shallow depth (11.5X), probably resulting in missing some simple and satellite repeats in Ogye_1.1. A similar tendency can be seen in the Golden-collared manakin genome (ASM171598v1) (**Figure 3**), which was also assembled in a hybrid manner using MaSuRCA assembler with high-depth Illumina short reads and low-depth PacBio long reads.

15,766 protein-coding and 6,900 lncRNA genes were annotated from twenty tissues of *YO*. 946 novel protein-coding genes were identified while 164 genes of *Galllus gallus red junglefowl* were missed in our annotations. In the case of lncRNAs, only about 13.6% of previously annotated chicken lncRNAs were redetected, and the remainders were mostly not expressed in *YO* or were false annotations, suggesting that the current chicken lncRNA annotations should be carefully examined. Our Ogye lncRNAs resembled previously annotated lncRNAs in mammals in their genomic characteristics, including transcript length, exon number, and tissue-specific expression pattern, providing evidence for the accuracy of the new annotations. Hence, our lncRNA catalogue may help us to improve lncRNA annotations in the chicken reference genome.

Availability of data

All of our sequence data and the genome sequence have been deposited in NCBI's Gene Expression Omnibus (GEO) superseries GSE 104358 and BioProject PRJNA412408. The all supporting data (genome

and gene sequence files, the expression tables of protein-coding and lncRNA genes, and the annotation files of RRBS, protein-coding, lncRNAs, SNPs, and INDELs) are available in the GigaScience repository (GigaDB).

Additional files

The supplementary figures and tables have been included in a supplementary file:

Figure S1. Assembly statistics of Ogye_1.1 gnome assembly at each step.

Figure S2. An example of mis-assembly.

Figure S3. Hierarchical mapping information in the reference-assisted additional assembly pipeline.

Figure S4. Alignment of the Ogye_1.1 genome to galGal4/5 drawn by MUMmer.

Figure S5. Mate-pair information in *FM* locus.

Table S1. Structural variations in the Ogye_1.1 genome

Table S2. Repeats in the Ogye_1.1 genome

Table S3. Repeat composition in different assemblies.

Table S4. 164 missed galGal4 protein-coding genes.

Acknowledgements

We thank all members of the BIG lab for helpful comments and discussions. This work was supported by the Cooperative Research Program for Agriculture Science and Technology Development (Project title: National Agricultural Genome Program, Project No. PJ01045301 and PJ01045303).

Author's Contributions

KTL, NSK, HHC, and JWN designed the study, KTL, YJD and CYC collected samples, DJL, HHC and KTL collected sequencing data, and JIS, KWN, NSK, JMK, HHC and JMN performed the analysis and developed the methodology. JIS, KWN, JMK, and JWN wrote the manuscript.

Competing interests

The authors declare that they have no competing interests.

Tables

Table 1. Summary of whole genome sequencing data

Table 2. Sequencing and mapping summary of RNA-seq data

Table 3. Sequencing and mapping summary of RRBS data

Table 4. Comparison of genome completeness using BUSCO

Table 5. Summary of methylated CpG sites across twenty tissues

Figure legends

Figure 1. A. A photograph of *Yeonsan Ogye* (*YO*), taken before sampling; **B.** Hybrid genome assembly pipeline comprising four steps, each utilizes a different set of sequencing reads; **C.** The N50 and average length of pseudo-contigs and scaffolds of the Ogye_1.1 and other avian genomes created using the indicated assembly methods (in the last column, sequencing platforms are designated as follows: "I" indicates Illumina, "P" is PacBio, "S" is Sanger, and "4" is Roche454).

Figure 2. A. Structural variation (SV) map of the Ogye_1.1 genome compared with galGal4 and galGal5. Insertions (red), deletions (blue), duplications (yellow), inversions (green), inter-chromosomal translocations (gray; Inter-translocation), and intra-chromosomal translocations (orange; Intra-translocation) are shown. SVs between the Ogye_1.1 genome and the galGal4 or 5 are shown with Venn diagrams; **B.** Three possible scenarios that could lead to the inverted duplication of the Fibromelanosis (*FM*) locus in the genomes of hyperpigmented chicken breeds; **C.** Copy gain of the *FM* locus, which includes the *end3* gene (indicated by the thin purple-shaded boxes), was identified on chromosome 20. The green- and yellow-shaded boxes indicate duplicated regions (Dupl_1 and Dupl_2, respectively) and the gray-shaded boxes indicate gaps (Gap_1 and Gap_2). The sizes of Gap_1 and Gap_2 were estimated to be 164.5 Kbp and 63.3 Kbp, respectively.

Figure 3. Composition of repeat elements in different assemblies of avian, reptile, and mammalian genomes. The repeats in unplaced scaffolds were not considered.

Figure 4. A. Gene (protein-coding and lncRNA) annotation maps of the Ogye_1.1 genome with TE, SNV/INDEL, and GC ratio landscapes are shown in a Circos plot. Color-codes indicate coverage (%) of TE in Mbp window, the number of protein-coding genes in Mbp window, the number of lncRNAs in Mbp window, SNP and INDEL frequency in 100Kbp window, and GC ratio in 100Kbp; **B.** A schematic flow of our protein-coding gene annotation pipeline; **C.** A Venn diagram showing the number of protein-coding genes in the Ogye_1.1 genome.

Figure 5. A. A computational pipeline for lncRNA annotations; **B.** The number of Ogye_1.1 and galGal4 lncRNAs are shown in the Venn diagram; **C.** Distribution of transcript length (red for lncRNAs and cyan for protein-coding genes). The vertical dotted lines indicate the median length; **D.** Distribution of exon number per transcript. Otherwise, as in **C**.

Figure 6. A. The circus plots illustrate the expression levels of protein-coding genes (left) and lncRNAs (right) across twenty tissues. The expression levels are indicated with Z-score, color-coded in the key; **B.** The expression patterns of the genes expressed with ≥ 10 FPKM in black tissues. Expression levels are indicated with a color-coded Z-score (red for low and blue for high expression) as shown in the key; **C**. Expression levels of *krt9* across twenty tissues; **D.** Expression levels of *lnc-lama2-1* across twenty tissues.

Figure 7. A. Principal component analysis (PCA) using tissue-specific protein-coding genes. PCs explaining the variances are indicated with the amount of the contribution in the left-top plot. PCA plots with PC1, PC2, and PC3 were demonstrated in a pairwise manner. Each tissue is indicated on the PCA plot with a specific color; **B.** PCA using tissue-specific lncRNAs. Otherwise, as in **A**.

Figure 8. A. The circus plot illustrates the CpG methylation levels in the promoters of protein-coding genes (left) and lncRNA (right) across twenty tissues. The methylation levels are indicated with a color-coded Z-score, described in the key; **B.** Hierarchical clustering using Pearson's correlation of DNA methylation patterns between tissues; **C-D.** Average DNA methylation landscape along gene bodies of protein-coding (**C**) and lncRNA (**D**) genes and their flanking regions across twenty tissues; **E-F.** Average DNA methylation level of the protein-coding (**E**) and lncRNA (**F**) genes in a tissue of maximum expression (red) and the other tissues (blue).

Figure 9. Spearman's correlation coefficients between the expression levels of genes and the methylation levels in the promoters of the genes are shown across chromosomes (heatmaps) in a circus plot. The bar charts indicate the count of genes (left for protein-coding genes and right for lncRNAs) with significant negative (red) and positive (cyan) correlations ($P < 0.05$) between the methylation level in their promoters and their expression values.

References

1. Domestic Animal Diversity Information System. http://dad.fao.org/.
2. Dorshorst B, Okimoto R and Ashwell C. Genomic regions associated with dermal hyperpigmentation, polydactyly and other morphological traits in the Silkie chicken. J Hered. 2010;101 3:339-50.
3. Dorshorst B, Molin AM, Rubin CJ, Johansson AM, Stromstedt L, Pham MH, et al. A complex genomic rearrangement involving the endothelin 3 locus causes dermal hyperpigmentation in the chicken. PLoS Genet. 2011;7 12:e1002412.
4. Arora G, Mishra SK, Nautiyal B, Pratap SO, Gupta A, Beura CK, et al. Genetics of hyperpigmentation associated with the Fibromelanosis gene (Fm) and analysis of growth and meat quality traits in crosses of native Indian Kadaknath chickens and non-indigenous breeds. Br Poult Sci. 2011;52 6:675-85.
5. Łukasiewicz M, Niemiec J, Wnuk A and Mroczek-Sosnowska N. Meat quality and the histological structure of breast and leg muscles in Ayam Cemani chickens, Ayam Cemani× Sussex hybrids and slow-growing Hubbard JA 957 chickens. Journal of the Science of Food and Agriculture. 2015;95 8:1730-5.
6. Dharmayanthi AB, Terai Y, Sulandari S, Zein MS, Akiyama T and Satta Y. The origin and evolution of fibromelanosis in domesticated chickens: Genomic comparison of Indonesian Cemani and Chinese Silkie breeds. PLoS One. 2017;12 4:e0173147.
7. It has been registered on UNESCO's Memory of the World Programme in 2009. http://www.unesco.org/new/en/communication-and-information/memory-of-the-world/.
8. Zhang GJ, Li C, Li QY, Li B, Larkin DM, Lee C, et al. Comparative genomics reveals insights into avian genome evolution and adaptation. Science. 2014;346 6215:1311-20.
9. Hillier LW, Miller W, Birney E, Warren W, Hardison RC, Ponting CP, et al. Sequence and comparative analysis of the chicken genome provide unique perspectives on vertebrate evolution. Nature. 2004;432 7018:695-716.
10. Warren WC, Hillier LW, Tomlinson C, Minx P, Kremitzki M, Graves T, et al. A New Chicken Genome Assembly Provides Insight into Avian Genome Structure. G3-Genes Genomes Genetics. 2017;7 1:109-17.
11. Miller SA, Dykes DD and Polesky HF. A simple salting out procedure for extracting DNA from human nucleated cells. Nucleic Acids Res. 1988;16 3:1215.
12. Luo R, Liu B, Xie Y, Li Z, Huang W, Yuan J, et al. SOAPdenovo2: an empirically improved memory-efficient short-read de novo assembler. Gigascience. 2012;1 1:18.
13. Salmela L and Rivals E. LoRDEC: accurate and efficient long read error correction. Bioinformatics. 2014;30 24:3506-14.
14. Gnerre S, Maccallum I, Przybylski D, Ribeiro FJ, Burton JN, Walker BJ, et al. High-quality draft assemblies of mammalian genomes from massively parallel sequence data. Proc Natl Acad Sci U S A. 2011;108 4:1513-8.
15. Boetzer M and Pirovano W. SSPACE-LongRead: scaffolding bacterial draft genomes using long read sequence information. Bmc Bioinformatics. 2014;15 1:211.
16. Gao S, Sung WK and Nagarajan N. Opera: reconstructing optimal genomic scaffolds with high-throughput paired-end sequences. J Comput Biol. 2011;18 11:1681-91.
17. Harris R. *Improved pairwise alignment of genomic DNA.* PhD Thesis, 2007.

18. English AC, Richards S, Han Y, Wang M, Vee V, Qu J, et al. Mind the gap: upgrading genomes with Pacific Biosciences RS long-read sequencing technology. PLoS One. 2012;7 11:e47768.
19. Sohn JI and Nam JW. The present and future of de novo whole-genome assembly. Brief Bioinform. 2016:bbw096. doi:10.1093/bib/bbw096.
20. McKenna A, Hanna M, Banks E, Sivachenko A, Cibulskis K, Kernytsky A, et al. The Genome Analysis Toolkit: a MapReduce framework for analyzing next-generation DNA sequencing data. Genome Res. 2010;20 9:1297-303.
21. VecScreen https://anonsvn.ncbi.nlm.nih.gov/repos/v1/trunk/c++/ and UniVec database https://www.ncbi.nlm.nih.gov/tools/vecscreen/univec/.
22. Simao FA, Waterhouse RM, Ioannidis P, Kriventseva EV and Zdobnov EM. BUSCO: assessing genome assembly and annotation completeness with single-copy orthologs. Bioinformatics. 2015;31 19:3210-2.
23. Rausch T, Zichner T, Schlattl A, Stutz AM, Benes V and Korbel JO. DELLY: structural variant discovery by integrated paired-end and split-read analysis. Bioinformatics. 2012;28 18:i333-i9.
24. Layer RM, Chiang C, Quinlan AR and Hall IM. LUMPY: a probabilistic framework for structural variant discovery. Genome Biol. 2014;15 6:R84.
25. Li H. FermiKit: assembly-based variant calling for Illumina resequencing data. Bioinformatics. 2015;31 22:3694-6.
26. Chong Z, Ruan J, Gao M, Zhou W, Chen T, Fan X, et al. novoBreak: local assembly for breakpoint detection in cancer genomes. Nat Methods. 2017;14 1:65-7.
27. Tempel S. Using and understanding RepeatMasker. Mobile Genetic Elements: Protocols and Genomic Applications. 2012:29-51.
28. Bao W, Kojima KK and Kohany O. Repbase Update, a database of repetitive elements in eukaryotic genomes. Mob DNA. 2015;6 1:11.
29. Picard Tools. http://broadinstitute.github.io/picard/.
30. Dobin A, Davis CA, Schlesinger F, Drenkow J, Zaleski C, Jha S, et al. STAR: ultrafast universal RNA-seq aligner. Bioinformatics. 2013;29 1:15-21.
31. Pertea M, Pertea GM, Antonescu CM, Chang TC, Mendell JT and Salzberg SL. StringTie enables improved reconstruction of a transcriptome from RNA-seq reads. Nat Biotechnol. 2015;33 3:290-5.
32. TransDecoder. https://github.com/TransDecoder/TransDecoder/.
33. Wang L, Park HJ, Dasari S, Wang SQ, Kocher JP and Li W. CPAT: Coding-Potential Assessment Tool using an alignment-free logistic regression model. Nucleic Acids Research. 2013;41 6:e74-e.
34. Kong L, Zhang Y, Ye ZQ, Liu XQ, Zhao SQ, Wei L, et al. CPC: assess the protein-coding potential of transcripts using sequence features and support vector machine. Nucleic Acids Research. 2007;35 suppl_2:W345-W9.
35. You B-H, Yoon S-H and Nam J-W. High-confidence coding and noncoding transcriptome maps. Genome research. 2017;27 6:1050-62.
36. Trapnell C, Williams BA, Pertea G, Mortazavi A, Kwan G, Van Baren MJ, et al. Transcript assembly and quantification by RNA-Seq reveals unannotated transcripts and isoform switching during cell differentiation. Nature biotechnology. 2010;28 5:511-5.

37. Zhao Y, Li H, Fang S, Kang Y, Hao Y, Li Z, et al. NONCODE 2016: an informative and valuable data source of long non-coding RNAs. Nucleic acids research. 2016;44 D1:D203-D8.
38. Yeo G and Burge CB. Maximum entropy modeling of short sequence motifs with applications to RNA splicing signals. Journal of computational biology. 2004;11 2-3:377-94.
39. Pauli A, Valen E, Lin MF, Garber M, Vastenhouw NL, Levin JZ, et al. Systematic identification of long noncoding RNAs expressed during zebrafish embryogenesis. Genome research. 2012;22 3:577-91.
40. Weikard R, Hadlich F and Kuehn C. Identification of novel transcripts and noncoding RNAs in bovine skin by deep next generation sequencing. BMC genomics. 2013;14 1:789.
41. Billerey C, Boussaha M, Esquerré D, Rebours E, Djari A, Meersseman C, et al. Identification of large intergenic non-coding RNAs in bovine muscle using next-generation transcriptomic sequencing. BMC genomics. 2014;15 1:499.
42. Al-Tobasei R, Paneru B and Salem M. Genome-wide discovery of long non-coding RNAs in rainbow trout. PLoS One. 2016;11 2:e0148940.
43. reshape2. https://github.com/hadley/reshape.
44. Krueger F and Andrews SR. Bismark: a flexible aligner and methylation caller for Bisulfite-Seq applications. Bioinformatics. 2011;27 11:1571-2..
45. Laurent L, Wong E, Li G, Huynh T, Tsirigos A, Ong CT, et al. Dynamic changes in the human methylome during differentiation. Genome Res. 2010;20 3:320-31..
46. Huang YZ, Sun JJ, Zhang LZ, Li CJ, Womack JE, Li ZJ, et al. Genome-wide DNA methylation profiles and their relationships with mRNA and the microRNA transcriptome in bovine muscle tissue (Bos taurine). Sci Rep. 2014;4:6546.
47. Laine VN, Gossmann TI, Schachtschneider KM, Garroway CJ, Madsen O, Verhoeven KJ, et al. Evolutionary signals of selection on cognition from the great tit genome and methylome. Nat Commun. 2016;7:10474.
48. Li A, Zhou ZY, Hei X, Otecko NO, Zhang J, Liu Y, et al. Genome-wide discovery of long intergenic noncoding RNAs and their epigenetic signatures in the rat. Sci Rep. 2017;7 1:14817.

 Click here to download Table Table1_WGS.docx

Table 1. Summary of whole genome sequencing data

Platform	Library type	Insert-size	No. of read (10^6)	Total base (Gbp)	Coverage (X)	SRA accession
Illumina HiSeq 2000	Paired-end	280 bp	129.6	19.5	18.6	SRR6189087
			124.5	18.7	17.8	SRR6189084
		500 bp	43.6	6.6	6.2	SRR6189095
			47.3	7.1	6.8	SRR6189097
			14.0	2.1	2.0	SRR6189096
			14.1	2.1	2.0	SRR6189098
			14.6	2.2	2.1	SRR6189082
			28.7	4.3	4.1	SRR6189094
	Mate-pair	3Kbp	146.5	21.8	20.8	SRR6189093
			135.0	20.1	19.1	SRR6189083
		5Kbp	114.8	17.1	16.3	SRR6189081
			106.4	15.6	15.1	SRR6189088
		8Kbp	136.6	20.4	19.4	SRR6189085
			135.3	20.2	19.2	SRR6189086
		10Kbp	169.1	25.2	24.0	SRR6189091
			157.9	23.5	22.4	SRR6189092
	FOSMID	40Kbp	169.9	17.6	16.3	SRR6189089
PacBio RS II	Long read	6Kbp (ave. read length)	1.7	12.1	11.5	SRR6189090

Table 2. Sequencing and mapping summary of RNA-seq data

Samples	Paired-end			Single-end		
	No. of reads	Mapping rate	SRA accession	No. of reads	Mapping rate	SRA accession
Breast	34,893,064	92.05%	SRX3223583	43,294,022	90.70%	SRX3223603
Liver	33,476,266	85.75%	SRX3223584	48,032,813	85.81%	SRX3223604
Bone marrow	30,975,506	85.00%	SRX3223585	40,286,974	87.99%	SRX3223605
Fascia	33,316,764	84.61%	SRX3223586	42,425,452	87.93%	SRX3223606
Cerebrum	30,887,821	89.95%	SRX3223587	46,455,658	92.32%	SRX3223607
Gizzard	31,537,118	84.00%	SRX3223588	38,689,871	85.82%	SRX3223608
Immature egg	32,009,437	87.73%	SRX3223589	32,048,703	87.80%	SRX3223609
Comb	31,936,332	85.34%	SRX3223590	37,985,049	87.76%	SRX3223610
Spleen	28,946,777	89.70%	SRX3223591	38,704,448	89.33%	SRX3223611
Mature egg	30,873,699	91.98%	SRX3223592	40,650,664	92.17%	SRX3223612
Cerebellum	30,798,145	93.53%	SRX3223593	39,940,946	93.34%	SRX3223613
Gall bladder	35,862,229	84.83%	SRX3223594	35,423,339	87.06%	SRX3223614
Kidney	29,953,007	87.25%	SRX3223595	39,894,009	89.99%	SRX3223615
Heart	30,986,431	94.14%	SRX3223596	45,951,338	91.49%	SRX3223616
Uterus	33,444,002	91.89%	SRX3223597	46,650,355	90.63%	SRX3223617
Pancreas	30,595,568	82.52%	SRX3223598	47,361,192	84.35%	SRX3223618
Lung	31,533,498	87.63%	SRX3223599	45,552,982	92.34%	SRX3223619
Skin	34,442,464	82.36%	SRX3223600	41,934,970	84.00%	SRX3223620
Eye	33,006,509	89.21%	SRX3223601	44,044,630	91.82%	SRX3223621
Shank	28,643,334	94.07%	SRX3223602	47,716,995	79.86%	SRX3223622

Table 3. Sequencing and mapping summary of RRBS data

Samples	No. of reads	Mapping rate	SRA accession
Breast	6,042,106	68.90%	SRX3223667
Liver	6,744,208	74.20%	SRX3223668
Bone marrow	5,736,011	72.00%	SRX3223669
Fascia	5,720,194	68.90%	SRX3223670
Cerebrum	6,078,989	70.00%	SRX3223671
Gizzard	5,731,878	69.40%	SRX3223672
Immature egg	6,741,258	67.70%	SRX3223673
Comb	5,948,687	72.90%	SRX3223674
Spleen	6,307,517	77.60%	SRX3223675
Mature egg	6,246,607	69.20%	SRX3223676
Cerebellum	6,291,610	68.20%	SRX3223677
Gall bladder	5,738,180	70.10%	SRX3223678
Kidney	5,470,502	68.60%	SRX3223679
Heart	5,462,739	69.40%	SRX3223680
Uterus	6,046,764	67.90%	SRX3223681
Pancreas	7,100,215	70.30%	SRX3223682
Lung	5,640,120	67.60%	SRX3223683
Skin	7,226,309	72.40%	SRX3223684
Eye	6,956,141	71.90%	SRX3223685
Shank	5,924,463	74.20%	SRX3223686

Table 4. Comparison of genome completeness using BUSCO

Assembly	Complete		Fragment	Missing
	Single-copy	Duplication		
Ogye_1.1	97.60%	0.50%	0.90%	1.00%
galGal4	96.90%	0.90%	1.10%	1.10%
galGal5	97.40%	0.90%	0.70%	1.00%
Turkey_5.0	93.70%	0.50%	4.10%	1.70%
BGI_1.0	92.60%	0.40%	4.80%	2.20%
taeGut3.2.4	93.60%	2.20%	2.70%	1.50%

Table 5. Summary of methylated CpG sites across twenty tissues

	All genomic region			Promoter region		
	Total No. of site	Methylated CpG sites		Total No. of site	Methylated CpG sites	
		No. of site	Fraction (%)		No. of site	Fraction (%)
Breast	994,326	621,751	62.53	228,673	91,704	40.10
Liver	1,641,060	505,775	30.82	522,590	97,597	18.68
Bone marrow	1,096,466	671,781	61.27	254,978	100,385	39.37
Fascia	1,146,350	670,181	58.46	278,618	99,802	35.82
Cerebrum	1,246,514	748,323	60.03	298,677	112,689	37.73
Gizzard	1,024,125	609,010	59.47	234,379	85,273	36.38
Immature egg	1,416,686	809,214	57.12	334,813	115,195	34.41
Comb	1,035,966	642,138	61.98	239,319	92,436	38.62
Spleen	995,639	401,080	40.28	298,833	74,473	24.92
Mature egg	1,144,589	695,258	60.74	269,124	102,282	38.01
Cerebellum	1,279,666	775,513	60.60	305,489	117,950	38.61
Gall bladder	953,630	595,681	62.46	225,122	89,174	39.61
Kidney	1,016,035	610,941	60.13	238,066	89,255	37.49
Heart	1,000,957	611,343	61.08	235,853	90,434	38.34
Uterus	893,101	543,931	60.90	203,102	77,365	38.09
Pancreas	1,119,795	647,577	57.83	267,036	94,371	35.34
Lung	985,824	594,046	60.26	229,316	87,140	38.00
Skin	868,368	565,815	65.16	198,275	85,094	42.92
Eye	1,051,332	663,413	63.10	252,991	105,539	41.72
Shank	862,931	512,853	59.43	210,905	76,512	36.28

Figure 1

Click here to download Figure Figure1_Ogye_genome_assembly.pdf

A.

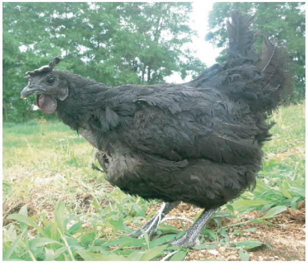

B.

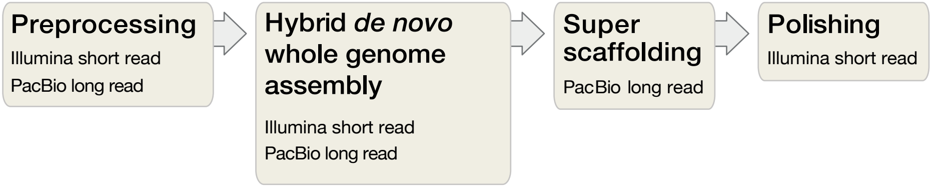

C.

Species	Assembly	Total length (Gbp)	Pseudo-contig			Scaffold			Gaps in scaffold		Assembly method	
			Number	Average length (Kbp)	N50 (Kbp)	Number	Average length (Kbp)	N50 (Mbp)	Total length (Mbp)	Fraction (%)	Assembler	Sequencing platform
Chicken (Yoensan Ogye)	Ogye_1.1	1.00	8,448	118.6	504.8	1,906	517.8	21.2	8.5	0.85	Our pipeline	I/P
Chichen	galGal4	1.05	27,143	38.1	279.0	915	1,128.8	12.9	14.1	1.34	Celara	S/4
	galGal5	1.23	24,698	49.3	2,894.8	23,870	51.0	6.4	11.8	0.96	MHAP/PBcR	I/S/4/P
Hoodedcrow	Hooded_Crow_genome	1.05	28,920	35.4	94.4	1,299	787.1	16.4	27.5	2.62	ALLPATHS-LG	I
Golden eagle	Aquila_chrysaetos-1.0.2	1.19	17,032	69.3	172.3	1,142	1,033.3	9.2	12.7	1.07	ALLPATHS-LG	I
Medium ground-finch	GeoFor_1.0	1.07	95,828	10.9	30.5	27,239	38.2	5.3	24.0	2.25	ALLPATHS-LG	I
Blue-crowned manakin	Lepidothrix_coronata-1.0	1.08	23,501	45.0	141.8	4,612	229.2	5.0	22.4	2.07	ALLPATHS-LG	I
White-throated sparrow	Zonotrichia_albicollis-1.0.1	1.05	37,661	26.7	112.7	6,018	167.2	4.9	46.3	4.40	ALLPATHS-LG	I
Silver-eye	ASM128173v1	1.04	65,519	15.3	32.2	2,933	341.5	3.6	34.3	3.31	ALLPATHS-LG	I
Tibetan ground-tit	PseHum1.0	1.04	27,052	38.1	165.3	5,406	190.5	16.3	13.0	1.24	SOAPdenovo	I
Bald eagle	Haliaeetus_leucocephalus-4.0	1.18	31,786	36.5	105.5	1,023	1,133.2	9.1	19.2	1.63	SOAPdenovo	I
American crow	ASM69197v1	1.09	89,646	11.7	29.1	10,547	99.7	7.0	39.5	3.62	SOAPdenovo	I
Saker falcon	F_cherrug_v1.0	1.17	75,898	15.2	31.3	5,863	196.3	4.2	23.8	2.03	SOAPdenovo	I
Peregrine falcon	F_peregrinus_v1.0	1.17	83,081	13.9	28.6	7,021	164.3	3.9	18.6	1.58	SOAPdenovo	I
Rock pigeon	Cliv_1.0	1.11	100,099	10.9	26.6	14,923	72.8	3.1	21.1	1.90	SOAPdenovo	I
Little egret	ASM68718v1	1.21	100,662	11.5	29.0	11,791	98.2	3.1	48.7	4.04	SOAPdenovo	I
Hoatzin	ASM69207v1	1.20	109,627	10.4	28.2	10,256	111.4	2.9	61.5	5.11	SOAPdenovo	I
Golden-collared manakin	ASM171598v1	1.21	29,998	38.9	185.6	15,315	76.3	16.6	45.5	3.75	MaSuRCA	I/P
Turkey	Turkey_5.0	1.13	296,315	3.7	26.7	233,806	4.7	3.8	35.3	3.13	MaSuRCA	IS/4
Parrot	Melopsittacus_undulatus_6.3	1.12	70,891	15.3	55.6	25,212	43.1	10.6	30.8	2.75	Celara	I/4
Zebra finch	Taeniopygia_guttata-3.2.4	1.23	124,806	9.8	38.6	37,422	32.7	8.2	9.3	0.75	PCAP	S

Figure 2

Click here to download Figure Figure2_variations.pdf

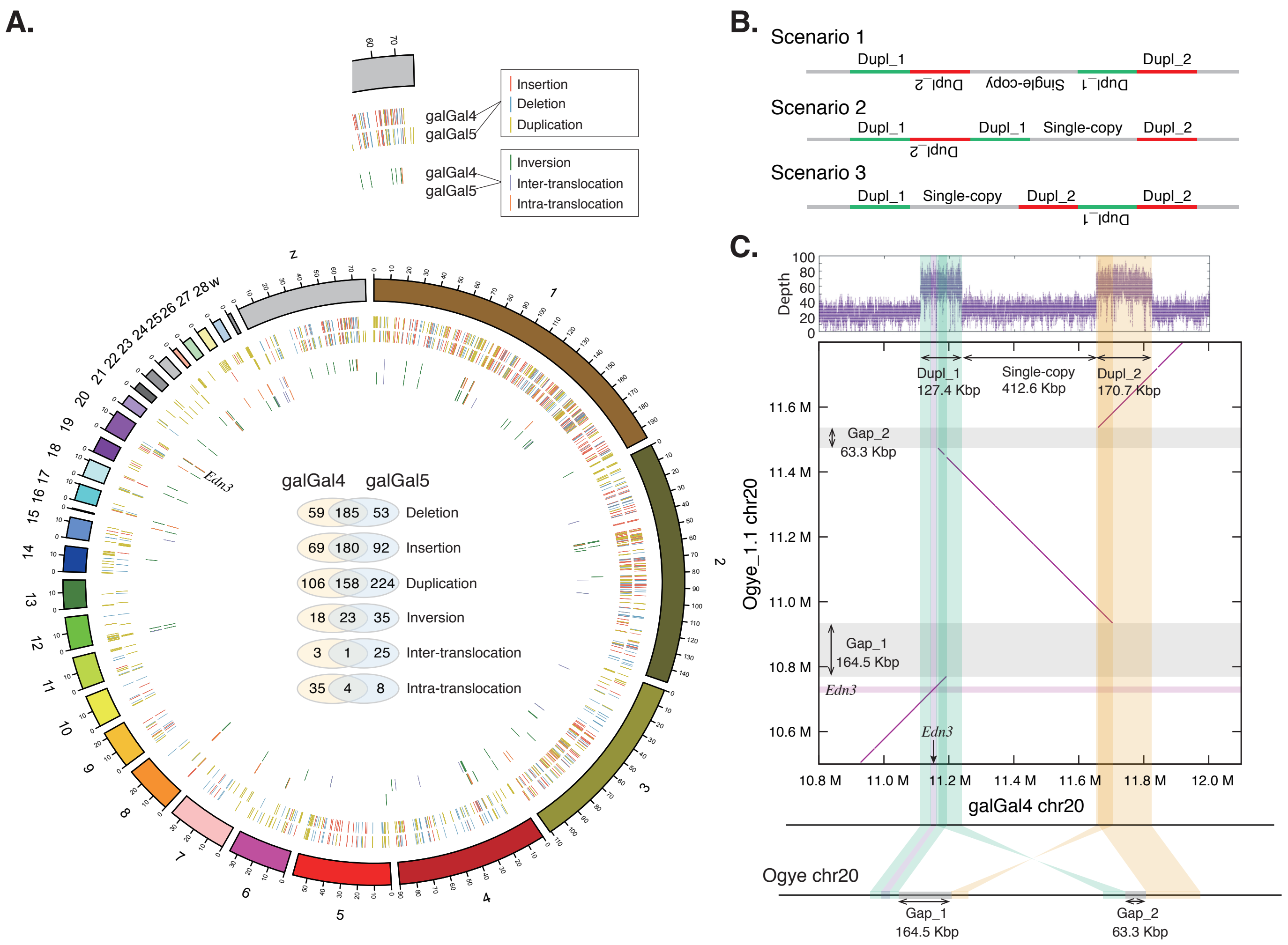

Figure 3

Click here to download Figure Figure3_repeats.pdf

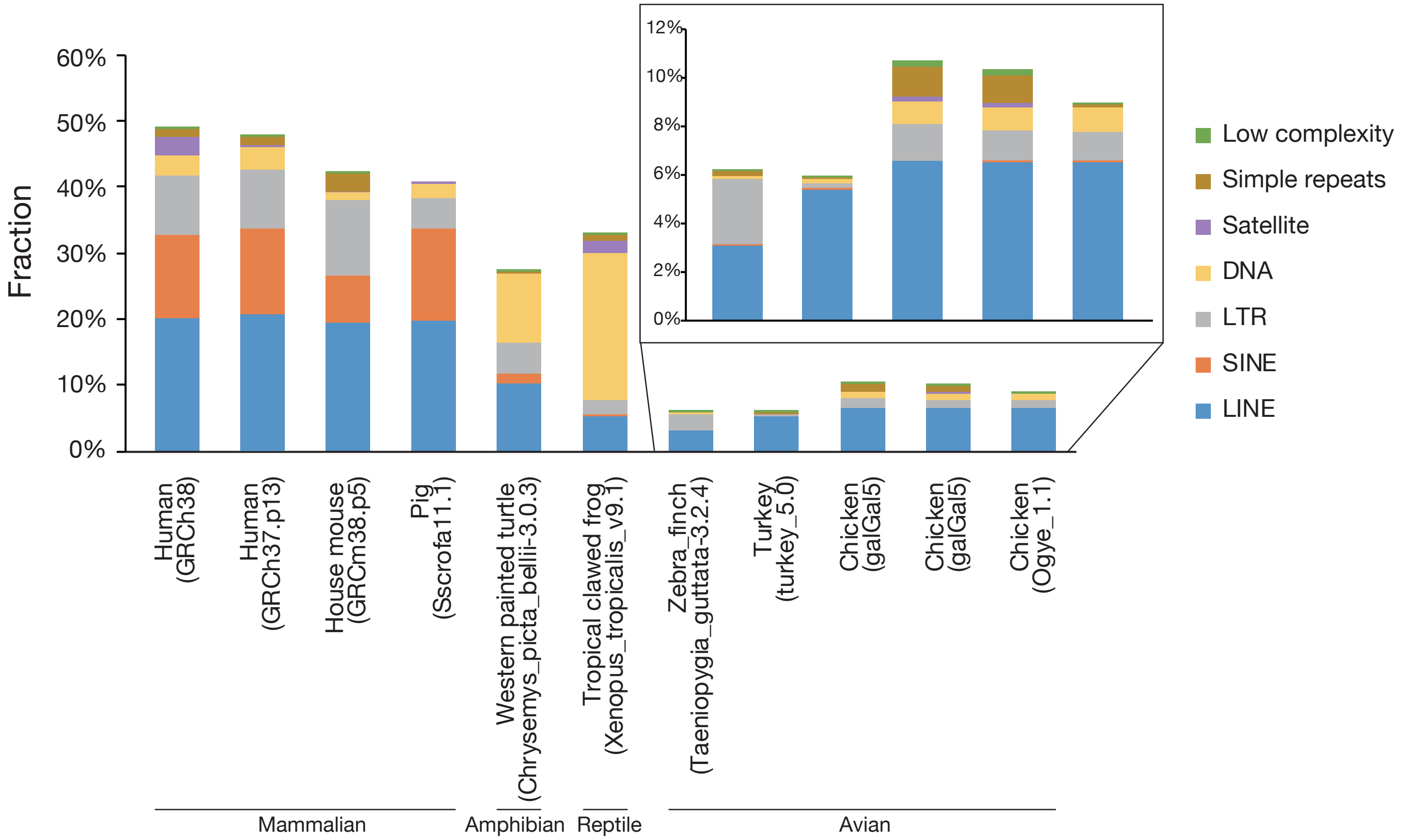

Figure 4 Click here to download Figure Figure4_annotations.pdf

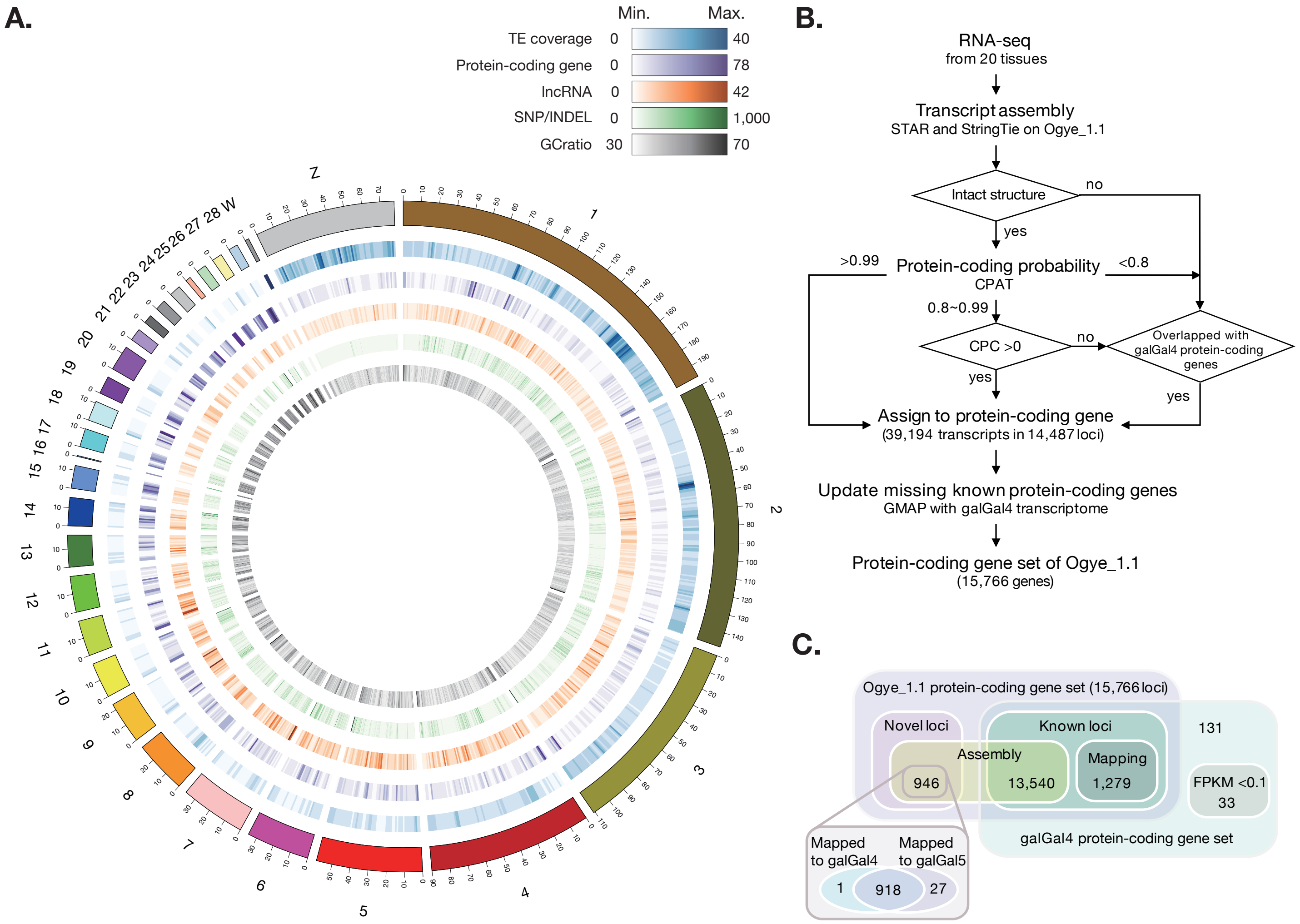

Figure 5

Click here to download Figure Figure5_lncRNA.pdf

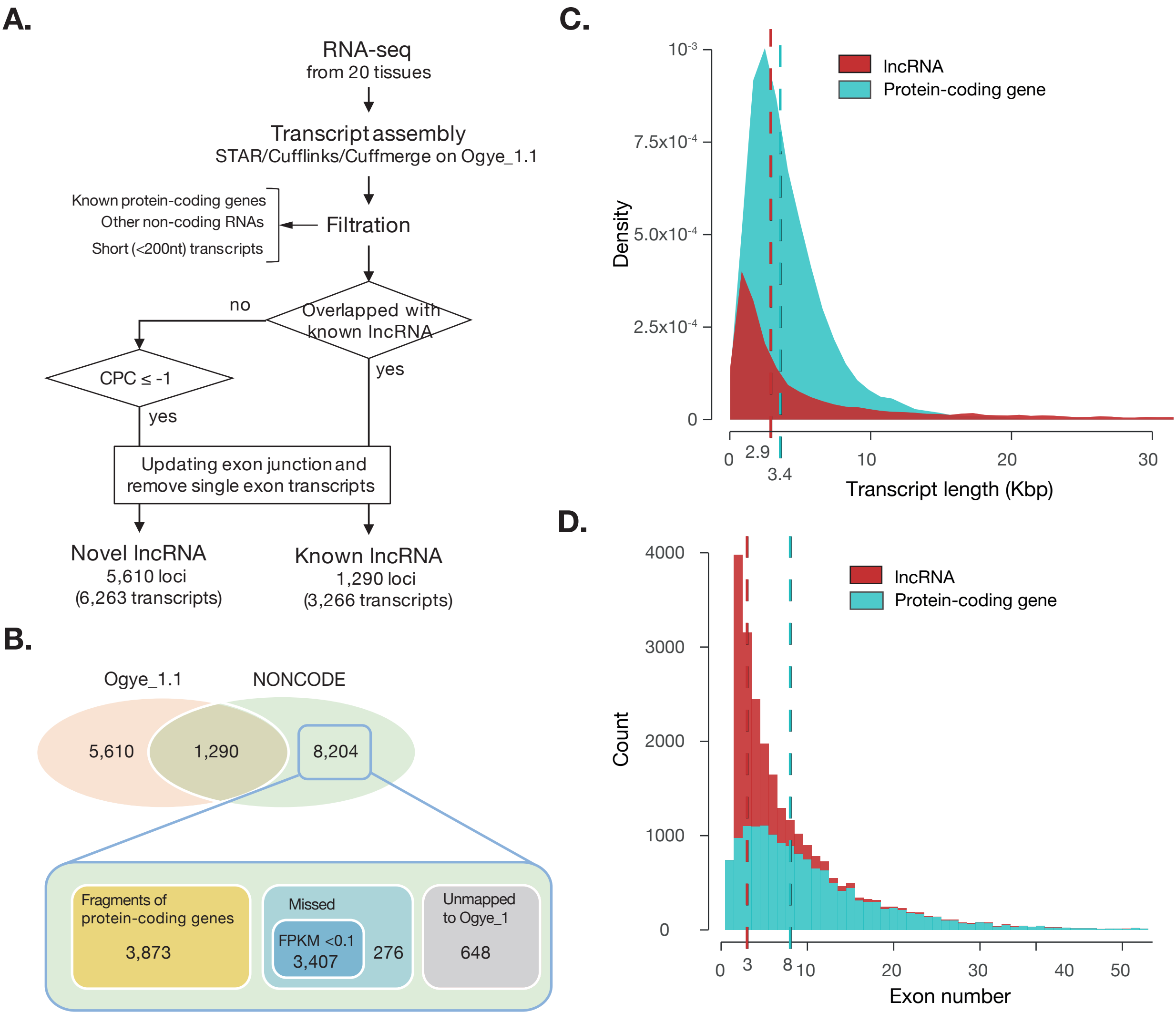

Figure 6 Click here to download Figure Figure6_ExpressionMap.pdf

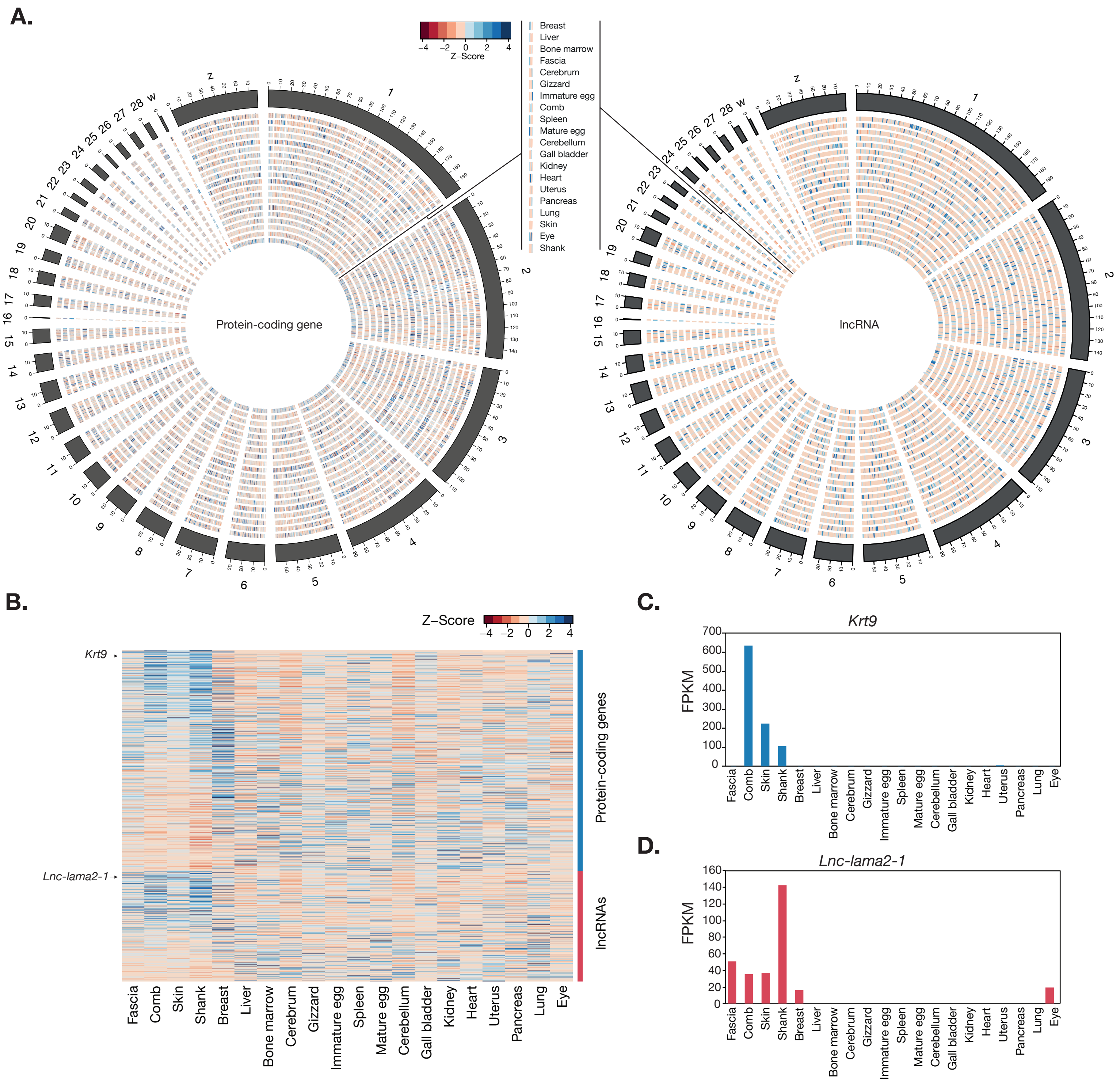

Figure 7

Click here to download Figure Figure7_PCA.pdf

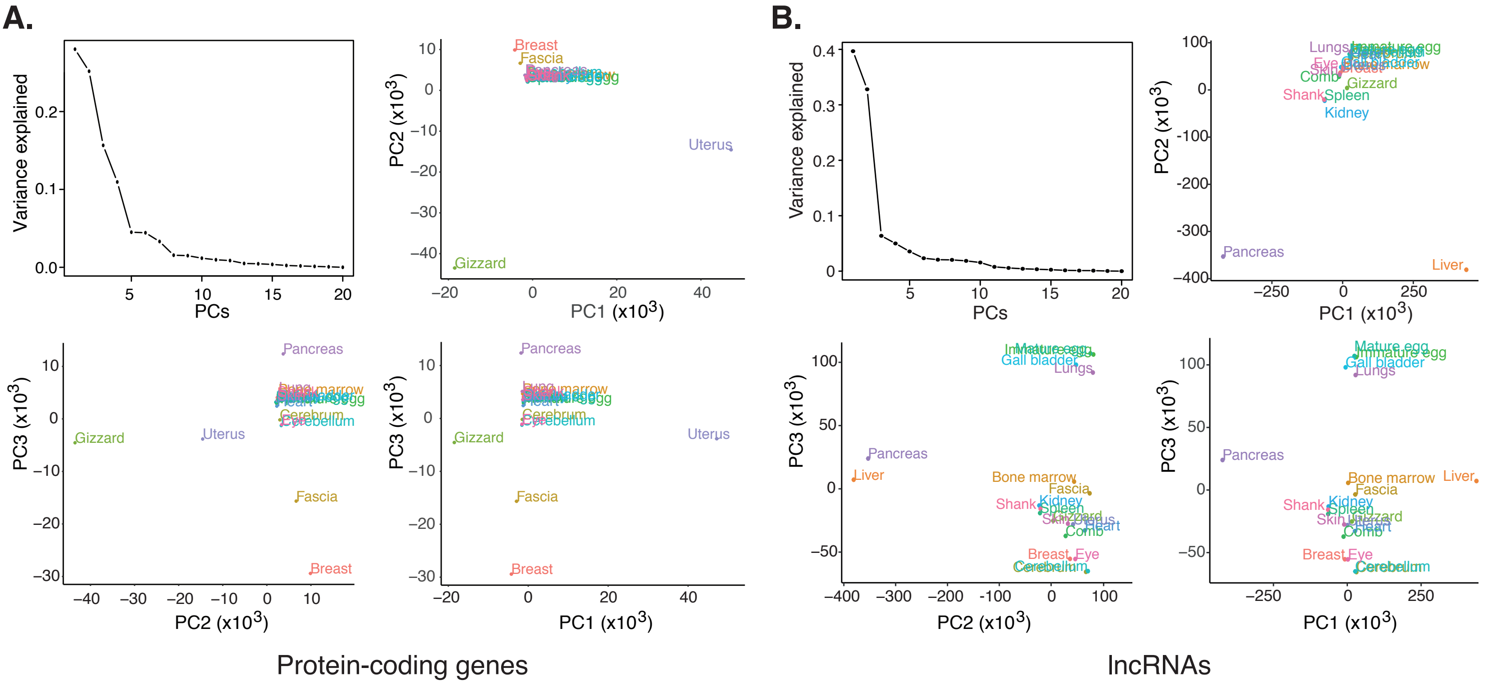

Figure 8

Click here to download Figure Figure8_Methylation.pdf

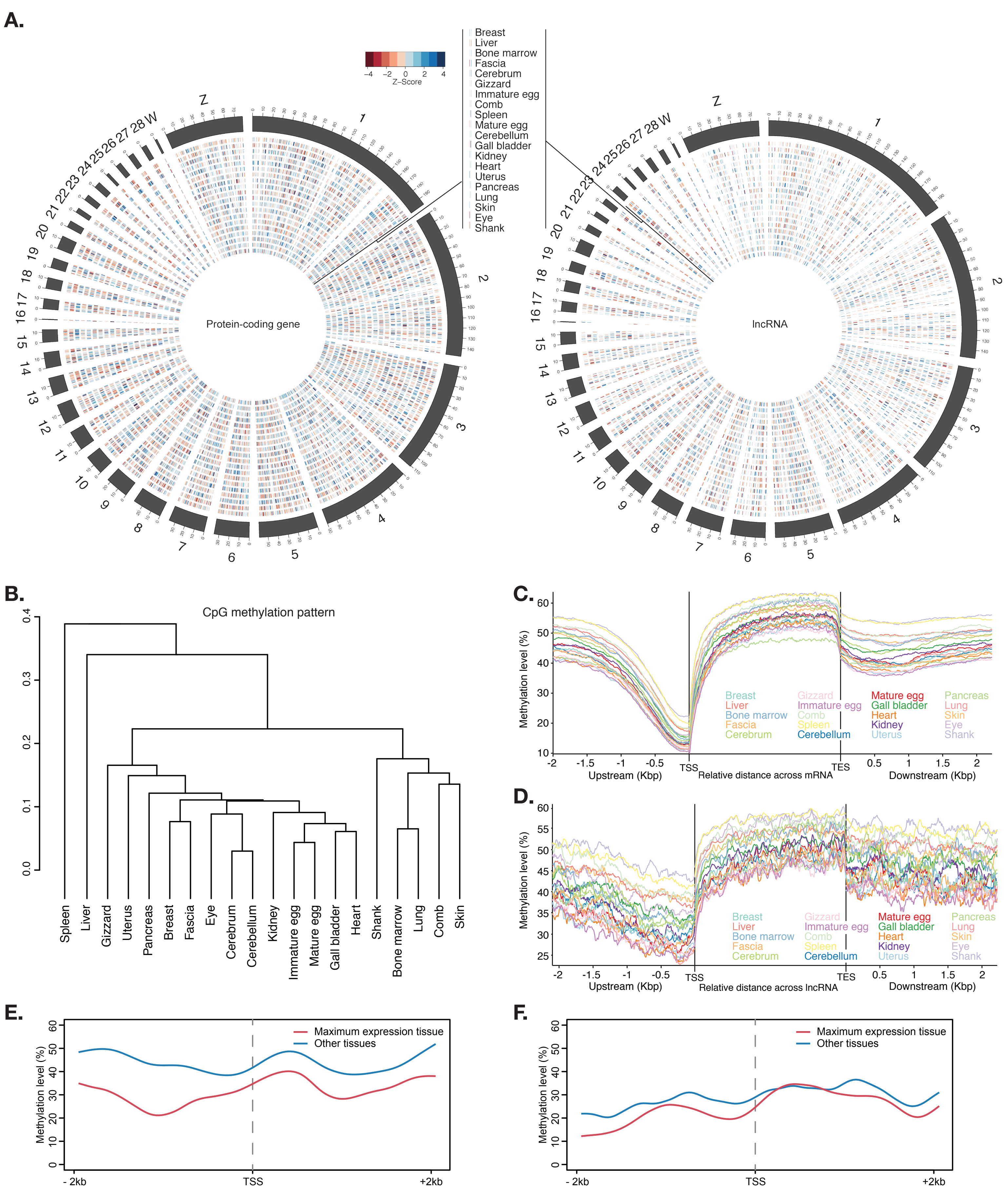

Figure 9

Click here to download Figure Figure9_Correlations.pdf

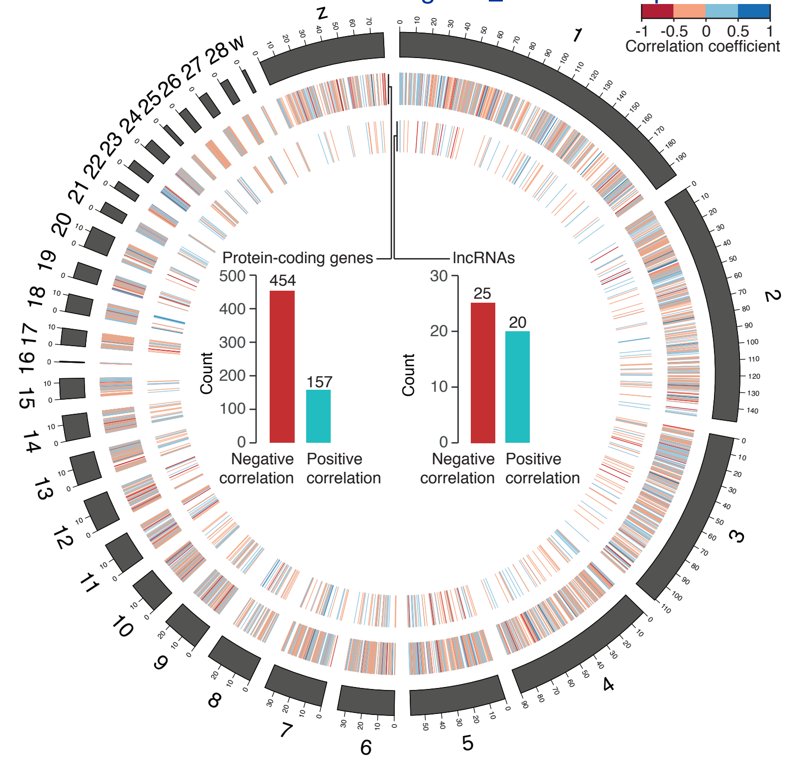

Click here to access/download
Supplementary Material
Ogye_GigaScience_20171208_SuppFigureTable.docx

Cover letter

Click here to download Personal Cover Ogye_GigaScience_20180108_CoverLetter.docx

Jan. 15, 2018

Jin-Wu Nam
Department of Life Science, College of Natural Sciences, Hanyang University, Seoul 133791, Republic of Korea
Telephone: +82-2-2220-2428
Fax: +82-2-2298-0319
jwnam@hanyang.ac.kr

Dear Editor,

Please find in the accompanying files our manuscript, "**Whole genome and transcriptome maps of the entirely black native Korean chicken breed *Yeonsan Ogye***" by Jang-il Sohn, Kyoungwoo Nam, Hyosun Hong, Jun-Mo Kim, Dajeong Lim, Kyung-Tai Lee, Yoon Jung Do, Chang Yeon Cho, Namshin Kim, Han-Ha Chai, and Jin-Wu Nam, for consideration as a Data Note in *GigaScience*.

Chicken breeds such as *Silkie* (white plumage and black skin) or *Ayam Cemani* (black plumage and skin) have received attention because of their unique hyperpigmentation phenotypes. Like *Ayam Cemani*, the indigenous Korean chicken breed *Yeonsan Ogye* (*YO*) has black plumage, skin, comb, shanks and internal organs, but also has unique phenotypes that set it apart from *Ayam Cemani*. Although chicken reference genomes (*gallus gallus red junglefowl*) have been released and are updated constantly, they may not be suitable for studying the genomes of the hyperpigmented chicken breeds (*Silkie*, *Ayam Cemani* or *YO*). For instance, hyperpigmented chicken breeds are known to have large structural variations in the Fibromelanosis locus (the so-called FM locus) on chromosome 20.

In this study, we present a whole genome *de novo* assembly of the *YO* genome. For this work, we produced and analyzed high-depth Illumina and low-depth PacBio whole genome sequencing (WGS) data from *YO*. Hybrid and pseudo-reference-assisted assembly was used to construct a *YO* draft genome, Ogye_1.1, whose quality is comparable with that of the chicken reference genome.

Although comprehensive coding and non-coding transcriptome maps have been competitively constructed and applied to understanding the functional roles of these sequences in humans and model animals, the maps of non-model animals or livestock are relatively poor across diverse developmental stages or tissues. We also reconstruct coding and non-coding transcriptome maps along with DNA methylation maps from twenty different tissues for our *YO* genome (Ogye_1.1). For this, massive RNA sequencing (RNA-seq) and reduced representation bisulfite sequencing (RRBS) data were produced from twenty different tissues of the same individual.

During the analysis of the non-coding transcriptome and methylation profiles, we found 9,529 long non-coding RNAs from 6900 genomic loci including 5,610 novel loci, leading to high-confidence lncRNA annotations. ~30 % of annotated lncRNAs in the reference chicken genome (galGal4) were not detected in our twenty tissues and

~35% are likely to be fragments of protein-coding genes or have coding potential, suggesting that chicken lncRNA annotations must be significantly improved. Our Ogye_1.1 lncRNA annotations may help to improve the quality of chicken lncRNA annotations.

We expect that the resulting genome sequence and transcriptome maps will be valuable resources for studying domestic breeds of chickens, including black-skinned chickens, as well as for understanding genomic differences and evolution of hyperpigmented chickens and functional elements related to hyperpigmentation.

Suitable referees include:

- Prof. Jae Yong Han (jaehan@snu.ac.kr) of Seoul National University, Republic of Korea
- Jianlin Han (h.jianlin@cgiar.org) International Livestock Research Institute (ILRI)
- Prof. Koji Tamura (tam@m.tohoku.ac.jp) of Tohoku University, Japan
- Yoko Satta (sattayk@soken.ac.jp) of The Graduate University for Advanced Science (Sokendai), Japan
- Prof. Yves Bigot (yves.bigot@tours.inra.fr) of Physiologie de la Reproduction et des Comportements, France

This manuscript has been seen and approved by all authors. All of our sequencing data and the genome sequence have been deposited in NCBI's BIOPROJECT under the BioProject number PRJNA412424 and Gene Expression Omnibus (GEO) superseries number GSE104358, and all processed and annotation data have been uploaded to the FTP site.

Thank you for consideration of our manuscript. We hope you will find it suitable for a Data Note of *GigaScience*.

Sincerely,

Jin-Wu Nam.
