# Supplementary material for: Whole genome and transcriptome maps of the entirely black native Korean chicken breed Yeonsan Ogye
Source: Gigascience. 2018 Jul 11;7(7):giy086. doi: 10.1093/gigascience/giy086 (PMC6065499; doi:10.1093/gigascience/giy086)
Supplement: GIGA-D-17-00321_Revision_1.pdf [file giy086_giga-d-17-00321_revision_1.pdf]

GigaScience

Whole genome and transcriptome maps of the entirely black native Korean chicken breed Yeonsan Ogye

--Manuscript Draft--

Manuscript Number:	GIGA-D-17-00321R1
Full Title:	Whole genome and transcriptome maps of the entirely black native Korean chicken breed Yeonsan Ogye
Article Type:	Data Note
Funding Information:	Rural Development Administration (PJ01045303) — Prof Jin-Wu Nam<br>Rural Development Administration (PJ01045301) — Ms. Han-Ha Chai
Abstract:	Yeonsan Ogye (YO), an indigenous Korean chicken breed (Gallus gallus domesticus), has entirely black external features and internal organs. In this study, the draft genome of YO was assembled using a hybrid de novo assembly method that takes advantage of high-depth Illumina short-reads (376.6X) and low-depth PacBio long-reads (9.7X). The contig and scaffold NG50s of the hybrid de novo assembly were 362.3Kbp and 16.8Mbp, respectively. The completeness (97.6%) of the draft genome (Ogye_1.1) was evaluated with single copy orthologous genes using BUSCO, and found to be comparable to the current chicken reference genome (galGal5; 97.4%; contigs were assembled with high-depth PacBio long-reads (50X) and scaffolded with short-reads), and superior to other avian genomes (92~93%; assembled with short-read-only or hybrid methods). Compared to galGal4 and 5, the draft genome included 551 structural variations including the Fibromelanosis (FM) locus duplication, related to hyperpigmentation. To comprehensively reconstruct transcriptome maps, RNA sequencing (RNA-seq) and reduced representation bisulfite sequencing (RRBS) data were analyzed from twenty different tissues, including four black tissues (skin, shank, comb, and fascia). The maps included 15,766 protein-coding and 6,900 long non-coding RNA genes, many of which were tissue-specifically expressed and displayed tissue-specific DNA methylation patterns in the promoter regions. We expect that the resulting genome sequence and transcriptome maps will be valuable resources for studying domestic chicken breeds, including black-skinned chickens, as well as for understanding genomic differences between breeds and the evolution of hyperpigmented chickens and functional elements related to hyperpigmentation.
Corresponding Author:	Jin-Wu Nam<br>Hanyang University<br>Seoul, KOREA, REPUBLIC OF
Corresponding Author Secondary Information:	
Corresponding Author's Institution:	Hanyang University
Corresponding Author's Secondary Institution:	
First Author:	Jang-il Sohn, Ph.D.
First Author Secondary Information:	
Order of Authors:	Jang-il Sohn, Ph.D.<br>Kyoungwoo Nam<br>Hyosun Hong<br>Jun-Mo Kim, Ph.D.<br>Dajeong Lim, Ph.D.<br>Kyung-Tai Lee, Ph.D.<br>Yoon Jung Do, Ph.D.

Powered by Editorial Manager® and ProduXion Manager® from Aries Systems Corporation

	Chang Yeon Cho, Ph.D.
	NamShin Kim, Ph.D.
	Jin-Wu Nam, Ph.D.
	Han-Ha Chai
**Order of Authors Secondary Information:**	
**Response to Reviewers:**	May 18, 2018<br><br>Dear Dr. Hans Zauner, and reviewers.<br><br>Thank you for sending the referee reports and inviting us to submit a revised manuscript. Please find in the accompanying files our manuscript, “Whole genome and transcriptome maps of the entirely black native Korean chicken breed Yeonsan Ogye” by Jang-il Sohn, Kyoungwoo Nam, Hyosun Hong, Jun-Mo Kim, Dajeong Lim, Kyung-Tai Lee, Yoon Jung Do, Chang Yeon Cho, Namshin Kim, Han-Ha Chai, and Jin-Wu Nam, for consideration as a Data Note in GigaScience.<br><br>We appreciate the reviewers’ many helpful comments and insights, which have led to substantial improvements to our paper. We have addressed all of the concerns of the referees, as detailed in our point-by-point response to their comments. Based on your helpful comments, our manuscript has been substantially improved.<br><br>We have attached CoverLetter and Point-by-point response files as response letters for editor and reviewers, respectively. The same response, written here, have been written in the files with tables and figures. Please find the files in attached files.<br><br>We hope that we have adequately responded to the reviewers’ inquiries and that you will find our revised manuscript suitable for publication in GigaScience.<br><br>Sincerely,<br><br>Jin-Wu Nam<br>Department of Life Science, College of Nature Sciences, Hanyang University, Seoul 133791, Republic of Korea<br>Telephone: +82-2-2220-2428<br>Fax: +82-2-2298-0319<br>jwnam@hanyang.ac.kr<br><br>==<br><br>* Comments are repeated with numbering, and our detailed responses are written below each comment. Every detailed response begins with “A:” and ends with dashes, “---------”.<br><br><br># Response to editor<br><br>1) Reviewer 2 mentions a number of points that should be discussed at least briefly in the manuscript (e.g. why Gal4 and not Gal5 was used, and a question (point 5) regarding the scaffolding procedure, among others).<br><br>A:<br>Both reviewers asked why galGal4 was used as a reference instead of galGal5. Because galGal4 was the most current version of chicken reference genome when we finished our Ogye whole genome assembly, we compared our draft genome to galGal4. Later on, galGal5 was released at NCBI (November 2015) and then published (Warren et al., 2017). We then additionally compared our Ogye draft genome with galGal5 when we analyzed the completeness of the draft genome using BUSCO and structural variations.<br><br>Regarding the scaffolding procedure (point 5 of reviewer 2), the scaffold N50 of the initial ALLPATHS-LG assembly was 10.7Mb, which jumped up to 27.8Mb after the

second scaffolding using PacBio and Fosmid data (Figure 1B and S1 in the revised manuscript). After breaking mis-assemblies, the N50 then increased to 18.7Mb. Further, we recognized that the word “scaffolding” might be confusing because there are several scaffolding steps in our pipeline. Hence, we have added a detailed description of the scaffolding steps to clarify this point in the revised manuscript on page 6 and 7 and have labeled the second and third scaffolding and gap-filling steps in Figure 1B.
----

2) I feel reviewer 1 is correct that some of the figures could be moved to the supplementary material - we don't have strict rules on the number of figures, but I would suggest to move at least the images demonstrating the pipelines (1B, 4B, 5A) to the supplemental material, to present the manuscript more concisely. Most of our readers are familiar with this type of analysis pipeline, so arguably don't need these figures to understand the work - but helpful to have it in the supplemental material! (But see also comment 2) of reviewer 2 regarding adding more information to Fig 1B - that seems also a good option for this figure).

A:
Based on these suggestions, we have moved Figures 4B and 5A (gene annotation pipelines) from the original manuscript to the supplemental figures in the revised manuscript. Regarding the whole genome assemble pipeline (Figure 1B), we decided to add detailed information to Figure 1B in the revised manuscript, based on the comment from reviewer 2.
----

3) Maybe you would like to also move a couple of the circus plots to the supplemental material as well, but that's up to you really - if you feel strongly about them, you can keep them in the main text.

A:
Following this suggestion, we have moved the Circos plots in Figures 2A, 4A, 6A, and 8A of the original manuscript to the supplemental materials but we kept the Circos plot of Figure 9 in Figure 6F in the revised version.
----

4) Both reviewer have good suggestions to improve reproducibility: I agree with reviewer 1 that a Supplemental file containing an inventory of commands, including runtime options, would be very useful.

A:
We agree with this suggestion. We have added a supplemental material file including all command lines with runtime options that we used in the analyses including whole genome assembly, transcriptome assembly and gene annotations, and so on.
----

5) Reviewer 2 suggests to include a README file for the dataset, including a short description of the data inventory. This is also a good suggestion; prior to publication our data curators will have a look at the GigaDB dataset and they will be in touch before publication, this would then also a good time to include a README file.

A:
According to this suggestion, we have added a README file including data description.
----

6) Finally, I also agree that the article would benefit from a native speaker reading over it once more before submission of the revised version.

A:
Following this suggestion, we have revised the manuscript, which was edited by a native speaker.

Powered by Editorial Manager® and ProduXion Manager® from Aries Systems Corporation

Response to reviewer #1:

Dr. Robert Kraus' special comment:
I like the article because it describes a rich and interesting data set of a very interesting chicken breed. We do not only get presented the genome and high quality assembly, but also various transcriptomes and methylomes. This includes not only annotation but also quantitative information and tissue specific expression atlases. I could not find major problems with this article. Analyses seem to be correctly performed and well described.

A:
We are very pleased that Dr. Robert Kraus thought that our study and results include valuable information with respect to not only genome assembly and annotations but also quantitative transcriptome and DNA methylome data. Very useful points were raised about specific details of our methods and descriptions of results and we feel that our manuscript has significantly benefited from making changes in response to all of the reviewer's major and minor comments.

Major comments:
1) During the review process it was hard to refer to places in the text because the lines numbers started over all over on each page; and on top of that, pages were not numbered… I counted page number on page 3 of the document that was available to me because the first two pages were info pages from the editorial office. Page three is the one where the abstract starts. E.g., P1L34 means line 34 on that first page.

A:
We are very sorry for causing the confusion about line numbers and for not including page numbers in the pdf file. In the revised version, we included the appropriate line numbers and page numbers in the main and supplementary manuscripts.
----

2) I further found that the quality of English could be improved. It is OK for now but the article would really benefit from a native speaker reading over it once more (I am not a native speaker myself, though). Where possible I indicated potential changes to the language but could not be exhaustive.

A:
As the reviewer suggested, we improved the quality of English throughout the manuscript by a commercial English editing service.
----

3) Data availability: I understand that this journal has (luckily!) high standards regarding data availability. The pipelines are well illustrated in the paper and also in the Supp Mat. However, not all runtime parameters are specified for all programmes. Often it is mentioned in the text. I am not sure now if this is sufficient, for a regular journal that does not focus so much on the technicalities I would suggest this is sufficient. For this journal, with its special emphasis on technicalities and bioinformatics protocols, I could imagine that a Supp Mat file containing an inventory of all the command incl. their runtime options would be useful. This may be added at the discretion of the Editor of this journal, if otherwise done regularly here.

A:
We absolutely agree with Robert's comment. All command lines with runtime options that we used for genome assembly, gene annotation, transcriptome assembly, RNA-seq and DNA methylation profiling are summarized in the file "Supplementary_Command.docx".

Specific comments
4) P1L34: gallus gallus domesticus -> Gallus gallus domesticus

A:
Thank you for the comment. We have corrected it as the reviewer suggested.

Powered by Editorial Manager® and ProduXion Manager® from Aries Systems Corporation

----

5) P1L40: I don't think you should present the definition of N50 in the abstract

A:
As the reviewer suggested, we have removed this definition from the abstract.
----

6) P1L46: The statement about Galgal5 using only long-read technology is not true. It also used previous data to build on - and that includes 1st and 2nd generation sequencing data - plus Illumina (>30x) was used for error correction. Let alone BAC sequencing and the likes.

A:
We sincerely thank the reviewer for this comment. Our intended meaning was that the genome assembly version of galGal5 took advantage of long read data along with other short read data. In detail, according to the manuscript about galGal5 assembly (Warren et al., 2017), contig assembly was done with 50X PacBio long reads, and then the resulting contigs were scaffolded with Fosmid paired-end sequences (3 Kb and 40 Kb insert-sizes) and with BAC end sequences, as the reviewer commented. Hence, we have changed the description from
“assembled with a long read-only method”
to
“contigs were assembled with high-depth PacBio long-reads (50X) and scaffolded with short reads”
----

7) P3L27: substitute "constructed" with "assembled"

A:
We have changed from “constructed” to “assembled”.
----

8) P3L47: include -> includes

A:
We have changed from “include” to “includes”.
----

9) P4L23: The sequencing library from one bird only or combined?

A:
The sequencing library was obtained from one an individual Yeonsan Ogye. We have found a typo “chickens”, which might have made you confused. Thus, we revised the phrase “8-month-old YO chickens” to “An 8-month-old YO chicken” on page 4 line 1. Additionally, we added a sentence “All sequencing data in this study (including data from whole genome sequencing, RNA-seq, and RRBS) were obtained from this sample bird.” on page 4 line 3. This revised sentence is also related to comments 17 and 18.
----

10) P4L39: The text in the bracket should be changed. It now reads as if most of reads were bad quality. Please clarify whether this was your threshold or observation.

A:
The phrase in the brackets was meant to refer to thresholds for the IlluQC_PRLL.pl module in the NGS QC Toolkit, i.e. options “-l 70 -s 20”, which are default options. Because the phrase was related to the preprocessing step in whole genome assembly, we have moved the phrase to the “Hybrid whole genome assembly” section, and modified a sentence from
“During the preprocessing step, the errors in the Illumina short reads were corrected by KmerFreq and Corrector [12].”
to
“In the preprocessing step, reads in which ≥ 30% of the nucleotides had a Phred score

	< 20 were excluded using NGS QC Toolkit (IlluQC_PRLL.pl) [36], the adaptor sequences of the remaining reads were removed using Trimmomatic [37], and three nucleotides at the 5'-end and five nucleotides at the 3'-end of the reads were trimmed using NGS QC Toolkit (TrimmingReads.pl). After quality control, the sequencing errors in the Illumina paired-end short-reads were corrected using KmerFreq and Corrector [38]." on page 6 line 6-11,<br>and from<br>"In total, 232.2 X Illumina short reads were obtained (59.6 X from the small insert libraries and 172.6 X from the large insert libraries) and, after filtering raw data with low quality (> 30% of the base-pairs in a read have a Phred score <20), 163.5X were used for genome assembly."<br>to<br>"In total, 376.6X raw Illumina short-reads (100.2X from the small insert libraries and 276.4X from the large insert libraries) were generated (Tables 1 and S1)" on page 4 line 14-15.<br>---------<br><br>11) P4L41: How do the 163.5x distribute across paired-end and mate-pair data?<br><br>A:<br>As we mentioned above, we updated the coverage of the total preprocessed short reads from 163.5X to 241.1X. Accordingly, we revised Tables 1 and S1 to include detailed information about the DNA sequencing data used for the whole genome assembly and downstream analyses including detailed coverages of paired-end and mate-pair data in the raw and preprocessed steps.<br>---------<br><br>12) P4L43: I would not say "scaffold quality", because quality is a complex thing. You could be more specific, and refer to "scaffold N50", or "improve scaffolding".<br><br>A:<br>Thank you for this comment. We have changed to "scaffold N50".<br>---------<br><br>13) P4L43: Which PacBio machine was used and which chemistry/version?<br><br>A:<br>PacBio long reads were produced by the PacBio RS II platform with P6C4 chemistry. Thus, we changed from<br>"11.5X PacBio long reads were additionally sequenced"<br>to<br>"9.7X PacBio long-reads were additionally sequenced using the PacBio RS II platform with P6C4 chemistry"<br>The coverage was changed to 9.7X due to using the estimated genome size, 1.25Gbp.<br>---------<br><br>14) P4L51: A reference should be given to the Trizol protocol/company where the reagent was purchased.<br><br>A:<br>The Trizol was purchased from Sigma-Aldrich. We changed from "TRIzol" to "TRIzol (Sigma-Aldrich®, St. Louis, USA)".<br>---------<br><br>15) P5L20: Unclear what "qualified" means, consider rephrasing<br><br>A:<br>We have rephrased "qualified using the Agilent …" to "the integrity of the cDNA libraries was examined using the Agilent …" to clarify the intended meaning on page 5 line 8-9. Similarly, we also rephrased "qualified using the Agilent …" to "and the integrity was examined using the Agilent …" on page 5 line 24.<br>---------<br><br>16) P5L24: tissues, which are -> tissues;

A:
As the reviewer suggested, we have changed this wording.
----

17) P5L29: All tissues from the same bird? The same bird for which genomic DNA was sequenced?

A:
Our RNA-seq data all comes from the same bird. To clarify this point, we revised the original sentence "… and shank (Table 2)." to "… and shank from the same bird (Table 2)."
----

18) P5L37: Again, was it from the same bird(s) as in the previous steps?

A:
We have RRBS data from twenty tissues from the same bird.
----

19) P5L46: How was ligation assessed?

A:
To avoid an unintended meaning, we have removed the phrase "After ligation had been assessed".
----

20) P6L25: In hybrid -> In the hybrid

A:
We have changed this wording to "In the hybrid".
----

21) P6L36: Why not aligned to Galgal5?

A:
As the reviewer pointed, galGal5 is the latest version of the chicken genome assembly. However, because the latest version of the chicken reference genome was galGal4 when we finished our Ogye whole genome assembly, we compared our draft genome to galGal4. Later on, galGal5 was released (November 2015). We then additionally compared our Ogye draft genome with galGal5 when we analyzed structural variations and the completeness of the draft genome using BUSCO.
----

22) P7L26: The mentioning of these stats are the best or second best values is superfluous. Actually, I am sure by now there are better stats for assemblies of zebra finch or hummingsbird, that were treated with the currently best possible protocols. This doesn't mean your stats are bad, not at all. But the statement does very likely not apply - and surely will not apply anymore within a really short amount of time. Delete it.

A:
We agree with the reviewer's comment and removed the statement.
----

23) P7L49: "by at least a different"; should that be …by at least four different SV prediction programs?

A:
We have changed from "by at least a different" to "by four different", and revised the following sentence, "SVs, validated by at least one program, included" to clarify.
----

24) P8L13: "three possible mechanistic scenarios" -> explain what scenarios

Powered by Editorial Manager® and ProduXion Manager® from Aries Systems Corporation

A:
We recognized that the original description and Figure 2B could be misleading; thus, we changed the sentence,
“A previous study suggested that the inverted duplication of the FM locus could be explained by three possible mechanistic scenarios (Figure 2B) [3].”
to
“A previous study, using conventional PCR assays, suggested three possible rearrangements at the FM locus [3]” on page 8 line 19-20.

And we also updated the Figure 2 legend to include a description of the three possible scenarios.

“Figure 2. Three possible scenarios for DNA rearrangements that could lead to the inverted duplication of the Fibromelanosis (FM) locus in the genomes of hyperpigmented chicken breeds; A. Three possible scenarios were developed based on the overlap patterns. The green and red lines indicated two duplicated genomic loci (Dupl_1 and Dupl_2, respectively) including the FM locus. Scenario 1 consists of a one-step rearrangement—an inverted duplication—whereas Scenarios 2 and 3 consist of a simultaneous rearrangement of an inverted duplication and an inversion; B. The read depth of a locus on chromosome 20 is shown in the top panel and the continuous/discontinuous patterns between mapped scaffolds are shown in the bottom panel. The star indicates the pattern that is discontinuous on both sides, validated in C; C. A comparison of the FM locus in galGal4 and the Ogye draft genome with aligned contigs (black lines) in each scaffold. The gray bands indicate the estimated gaps between contigs. The estimated sizes of Gap_1 and Gap_2 are 164.5Kbp and 63.3Kbp, respectively. The purple lines in the box indicate the END3 gene locus and the green and yellow shades indicate the duplicated regions (Dupl_1 and Dupl_2, respectively). The dark green and yellow shades indicate the discontinuous regions between scaffolds.”

In addition, we have updated the description in the main text as follow.
“To discern which rearrangement best fits our results, the FM loci from galGal4 and the Ogye draft were compared with the resulting scaffolds, showing an inverted duplication with discontinued scaffolds at both duplicated regions (Figures 2A; C). The results, with a discontinued scaffold on both sides, support rearrangement 1 rather than rearrangement 2 or 3, which have a discontinued scaffold on only one side. Although rearrangement 1 needs to be further validated, the FM locus in the Ogye_1.1 genome was updated according to the first rearrangement (Figure 2C).” on page 9 line 3-8.
----

25) P8L46: "and other genomes": what other genomes?

A:
We have changed from “Repeat elements in the Ogye_1.1 and other genomes were predicted” to “Repeat elements in the Ogye_1.1 and other genomes (human, mouse, pig, western painted turtle, tropical clawed frog, zebra finch, turkey, and chicken) were predicted” on page 9 line 12-13.
----

26) P9L4: the first word in the first line on this page, "to", should be removed, right?

A:
Thank you for this comment. We have removed the word.
----

27) P9L11: was depicted -> is depicted

A:
We have changed the phrase to “is depicted”.
----

28) P9L19: The number of SNPs seems little. How does this compare to the Galgal 4/5 or other, actually more outbred bird genomes?

A:
To investigate the small numbers of SNPs and INDELs, we checked and found a technical problem in the GATK pipeline that was installed in our system. To fix this problem, SNPs and INDELs were re-identified using VarScan 2 (Koboldt et al., 2012, Genome research), which found 3,206,794 SNPs and 302,463 INDELs. Hence, we have changed the description from
"As a result, 599,326 SNPs and 23,274 insertions/deletions (INDELs) were identified across the genome using Genome Analysis Toolkit (GATK) modules: HaplotypeCaller, combineGVCF, GenotypeGVCFs and VariantFiltration (with options "QD < 2.0 || FS > 200.0 || ReadPosRankSum < -20.0") [20]."
to
"We identified 3,206,794 SNPs and 302,463 INDELs across the genome using VarScan 2 with options --min-coverage 8 --min-reads2 2 --min-avg-qual 15 --min-var-freq 0.2 --p-value 1e-2 [57]." on page 10 line 2-4.
----

29) P10L11: Where are those 33 genes situated, as evidenced by comparative mapping. Exclusively on microchromosomes?

A:
We have checked where the 33 genes were assigned and found that the majority (26) of them were located on unknown chromosomes. We thus added a sentence as follows:
"Of the 33 missing genes, 26 appeared to be located on unknown chromosomes and the remainder are on autosomes (six genes) or the W sex chromosome (one gene) in galGal4." on page 10 line 23-24.

Additionally, we updated Table S5 (previously S4) to add the genomic positions of the 164 protein-coding genes missed in the Ogye_1.1 gene annotations.
----

30) P10L19: annotated -> annotate

A:
We corrected the word to "annotate".
----

31) P11L29: I am not sure what you mean by "which were dynamically changed". Please explain/rephrase.

A:
To avoid an unintended meaning and confusion, we removed the phrase. Thank you for the comment.
----

32) P12L12: It should be "in the genome" and not "in genomes", right?

A:
We corrected the phrase to "in the genome".
----

33) P12L31: Remove the two commas around "then".

A:
We removed two commas from "We, then, examined" to "We then examined".
----

34) P12L40: "specifically" should be changed to "specific", I think?

A:
We changed from "tissue specifically expressed genes" to "genes with tissue-specific expression".
----

35) P12L42: Should "only" be "one"? This sentence need to be clarified.

A:
We changed the original description to “280 protein-coding and 392 lncRNA genes with expression ≥ 10 FPKM in at least one tissue and with a maximum expression value four-fold higher than the mean expression level in twenty tissues” on page 13 line 11-13.
----

36) P12L53: noises -> noise

A:
We changed the word to “noise”.
----

37) P12L56: taken account -> taken into account

A:
We changed the phrase to “taken into account”.
----

38) P13L5: That should be "Discussion" in singular, right?

A:
We changed the word to “Discussion”.
----

39) P13L25-30: Cite the Lemur study and/or the MaSuRCA assembler

A:
We have cited the Lemur study and MaSuRCA assembler as follows.
“A similar tendency can be seen in the Golden-collared manakin genome (ASM171598v1) (Figure 3), which was also assembled in a hybrid manner using MaSuRCA assembler with high-depth Illumina short reads and low-depth PacBio long reads.”
to
“A similar tendency can be seen in the Golden-collared manakin genome (ASM171598v1) [32] (Figure 3) and the gray mouse lemur genome (Mmur3.0) [77], which were also assembled in a hybrid manner with high-depth Illumina short-reads and low-depth PacBio long-reads.”
including their references on page 14 line 7-9.
----

40) P13L59: Remove "The" at beginning of sentence.

A:
We removed “The” at the beginning of the sentence.
----

41) P14L17: gnome -> genome

A:
Thank you for this comment. We corrected the spelling to “genome”.
----

42) Table 1: You should also include a row that summarises the total of coverage (x) across all PE and mate-pair, and the total all. The numbers are in the text, but should re-appear in the table.

A:
At the reviewer’s suggestion, we added rows to summarize the total coverage (x) of paired-end reads, mate-pair reads, Illumina short-read totals, and the final totals, all in Table 1.

Powered by Editorial Manager® and ProduXion Manager® from Aries Systems Corporation

43) Table 4: Add a column that gives the species name.

A:
We have added a column giving species names in Table 4.
----

44) Table 5: all "site" should become plural, i.e., "sites".

A:
We changed all “site” entries to the plural “sites” in Table 5.
----

45) Figure 1C: Do I see correctly that is says "Chichen" and not "Chicken" in line 2? Also: Space missing between "Hooded" and "crow". Assemblies mentioned in this table should have footnotes to them and below the table cite the actual scientific reference articles.

A:
We have fixed to “Chicken” and “Hooded crow” in Figure 1C, and added scientific references in the table. We could not, however, find an appropriate reference for the Blue-crowned manakin genome.

Response to reviewer #2:

1) Why wasn't Galgal5 used in the overall analyses? (only Galgal4 used) It was presented in the beginning and end of the manuscript.

A:
galGal5 is the latest version of chicken genome assembly. However, because the latest version of the chicken reference genome was galGal4 when we finished the Ogye whole genome assembly, we compared our draft genome to galGal4. Later on, galGal5 was released at NCBI (November 2015) and published (Warren et al., 2017). We then additionally compared our Ogye draft genome with galGal5 when we analyzed structural variations and the completeness of the draft genome using BUSCO.
----

2) Figure 1B is referenced quite often in the manuscript, however the problem with 1B is that the figure is very simple and not detailed enough. You may want to break up 1B into its own figure, and substitute it with a variation of S1B.

A:
As the reviewer’s suggestion, we have replaced Figure 1B with S1A after making some updates, including the addition of detailed lists of programs used at each step.
----

3) When comparing N50 across different genomes/assemblies of varying sizes, you may want to use the NG50 metric as see in the assemblathon package.

A:
We appreciate this comment. In this study, although we used genome assembly results uploaded at NCBI, there is no guarantee that the sum of the resulting scaffolds or chromosomes is the same as the actual genome size due to unknown gaps between scaffolds. Hence, we changed the metric from N50 to NG50 using the estimated genome sizes acquired from the references in Figure 1C, as the reviewer suggested. However, for three genome assemblies (Little egret, Hoatzin, and Golden-collared manakin), for which there is no information about the estimated genome size in either the related paper or at NCBI Genome, the genome sizes were estimated with a k-mer counting method using KMC 2 (Deorowicz et al., 2015).
----

4) Which BUSCO dataset version did you use, odb9?

Powered by Editorial Manager® and ProduXion Manager® from Aries Systems Corporation

A:
We used OrthoDB v9 as the BUSCO dataset. We thus changed the text from “using BUSCO [22]” to “using BUSCO [49] with OrthoDB v9 (http://www.orthodb.org/)” on page 8 line 5-6.
----

5) As the scaffolding was reference guided, breaks were made, were joins also done using Galgal4 alignments? From the primary manuscript the overall N50 jumped from 10.7Mb to 18.7 Mb (initial ALLPATHS scaffold to pseudo contig/scaffold), but it sounds like it was breaks that did this. You may want to add a sentence to clear this up by saying the intermediate scaffolding and gapfilling pushed the N50 to 27Mb before breaking after aligning to Galgal4 down to 18.7Mb

A:
The scaffold N50 of the initial ALLPATHS-LG assembly was 10.7Mb, which jumped up to 27.8Mb after scaffolding and gap-filling using PacBio long-read and short-read data. In this step, no reference genome was used. As we found some mis-assemblies by genome-to-genome alignment (Figure S2A), breaks were made in the mis-assemblies. After breaking these mis-assemblies, the scaffold N50 was down to 18.7Mb. Given these results, we updated the description from

“Next, the scaffolds were additionally connected with corrected PacBio long reads and FOSMID reads using SSPACE-LongRead [15] and OPERA [16]. The gaps within and between scaffolds were re-examined with GapCloser [12] with error-corrected short reads. All resulting scaffolds were aligned to the galGal4 genome (GenBank assembly accession: GCA_000002315.2) by LASTZ [17].”
to
“In the second round of scaffolding and gap-filling (after the first scaffolding and gap-filling done during ASM1), the ASM1 scaffolds were connected with corrected PacBio long-reads using SSPACE-LongRead [41], and gaps within and between scaffolds were examined with error-corrected short-reads using GapCloser [38]. Then, the gap-filled scaffolds were connected again with FOSMID reads using OPERA [42] and the remaining gaps were re-examined with error-corrected short-reads using GapCloser, resulting in scaffolds with an N50 length of 27.8Mbp. However, some mis-assemblies (as illustrated in Figure S2A) were found by alignment of the resulting scaffolds with the galGal4 genome (GenBank assembly accession: GCA_000002315.2) using LASTZ [43].” on from page 6 line 20 to page 7 line 3.
----

6) Figure 3 shows Chicken(galGal5) twice, I expect one should be Galgal4. Also regarding figure 3, the end discussion section refers to the golden-collared manakin genome in figure 3, but this does not exist.

A:
Thank you for the comment. We fixed the typo and added golden-collared manakin in Figure3. Additionally, we found other typos and fixed them.
----

7) Figure 7, PCA plots, instead of having text as the components (which gets mixed up and illegible), consider using coloured data points which correspond to a legend.

A:
As the reviewer’s suggestion, we have moved the text to the legend and used colored data points in Figure 5 (originally Figure 7).
----

8) Data repository - would like to have a README file describing the datasets in repository.

A:
As the reviewer’s suggestion, we have added a README file that describes the datasets in the data repository. The contents of the README file are shown below.

Powered by Editorial Manager® and ProduXion Manager® from Aries Systems Corporation

[README]
Whole genome and transcriptome maps of the entirely black native Korean chicken breed Yeonsan Ogye
==

Directory lists:
1 - lncRNA
2 - Protein_coding_gene
3 - RRBS
4 - SNPs_INDELs
5 - Repeat
6 - Genome

Files:
1. lncRNA/
ogye1.1_lncRNA_annotation.gtf
lncRNA annotations comprising 6,900 novel and known lncRNAs identified from 20 tissues of Ogye
ogye1.1_lncRNA_expression_table.txt
Tab-delimited expression table of lncRNAs. Fragments Per Kilobase of exons per million mapped reads (FPKM) was calculated using the RSEM program. The values across tissues were normalized using the quantile normalization method.

2. Protein_coding_gene/
ogye1.1_PCG_annotation.gtf
Protein-coding gene annotations comprising 15,766 genes identified from 20 tissues.

ogye1.1_PCG_CDS.fasta
CDS sequences of ogye1.1 protein-coding genes.

ogye1.1_PCG_GENE.fasta
Gene sequences of ogye1.1 protein-coding genes.

ogye1.1_PCG_SE_expression_table.xlsx
The tab-delimited expression table of protein-coding genes using single-end RNA seq libraries. The expression (FPKM) was calculated using RSEM programThe values across tissues were normalized using the quantile normalization method.

ogye1.1_PCG_PE_expression_table.xlsx
The tab-delimited expression table of protein-coding genes using paired-end RNA seq libraries. The expression (FPKM) was calculated using RSEM programThe values across tissues were normalized using the quantile normalization method.

3. RRBS/
ogye1.1_RRBS_[xxxxxx].bedgraph, where [xxxxxx] is a tissue name.
Tab-delimited CpG methylation signal across 20 tissues. The signals were calculated using the Bismark CpG coverage report, which Include 1-based genomic coordinates for every covered cytosine position in each sample in the following format:
<chromosome> <start position> <end position> <methylation percentage> <count methylated> <count non-methylated>.

4. SNPs_INDEL/
ogye1.1.total.snps.filtered.vcf
SNP and insertion and deletion (INDEL) were called using VarScan 2 with options -mfileup2snp --min-coverage 8 --min-reads2 2 --min-avg-qual 15 --min-var-freq 0.2 --p-value 1e-2.

ogye1.1.total.indels.filtered.vcf
SNP and insertion and deletion (INDEL) were called using VarScan 2 with options -

mfileup2indel --min-coverage 8 --min-reads2 2 --min-avg-qual 15 --min-var-freq 0.2 --p-value 1e-2.

5. Repeat/
ogye1.1.fasta.out
Repeat lists identified from an input genome sequence using the RepeatMasker ver.4-0-6.

ogye1.1.fasta.tbl
The summary table of repeats identified from an input genome sequence using the RepeatMasker ver.4-0-6.

6. Genome/
ogye1.1.fasta
A genome sequence of Yeonsan Ogye genome assembly version 1.1., which include chromosome sequences and unplaced sequences.

===

Other changes in the revised manuscript

In the initial assembly, the short-read datasets were originally split into two groups, each of which was used for de novo assembly (ASM1 and AMS2) using ALLPATHS-LG. The results of ASM1 were subjected to the second scaffolding step, whereas the results of ASM2 were used in the pseudo-reference-assisted assembly step. To clarify the methods, we have added a detailed description of the whole genome assembly pipeline, which was missing from the original manuscript, as below.
"Additionally, another assembly (ASM2) was built with 109.2X paired-end and mate-pair reads that were unused in the initial assembly (see Table 1) using ALLPATHS-LG, resulting in 34,539 contigs with an N50 length of 59.2Kbp. The resulting ASM2 contigs were then subjected to the pseudo-reference-assisted assembly step"
on page 6 line 17-20

and changed from
"Next, the scaffolds were additionally connected"
to
"In the second round of scaffolding and gap-filling (after the first scaffolding and gap-filling done during ASM1), the ASM1 scaffolds were connected" on page 6 line 20-21.

Also, we changed from
"Based on the results, super-scaffolding and additional gap-filling was performed by SSPACE-LongRead and PBJelly, respectively, resulting in scaffold N50 of 21.2Mbp (Figure 1C)"
to
"Based on these results, a third round of scaffolding and gap-filling was performed with the long reads and the ASM2 contigs in each chromosome group using SSPACE-LongRead and PBJelly [46], respectively, resulting in a scaffold N50 of 21.2Mbp with 0.85% gaps (Figure S1)"
on page 7 line 15-18.

To reflect these changes, we have modified Figure 1B, with some changes in the polishing and finishing step.

Additionally, we found some errors in the read numbers and coverages of the whole genome sequencing data in Tables 1 and S1. The original numbers came from partially preprocessed rather than raw data. We thus have updated the numbers throughout the manuscript, Table1 and Table S1 using the estimated genome size of 1.25Gbp. Now, the total coverage of raw short reads is 376.6X and the coverage of preprocessed short reads is 241.1X.

Powered by Editorial Manager® and ProduXion Manager® from Aries Systems Corporation

	In addition, to clarify our intended meaning of the term "pseudo-contig", we updated our description from<br>"Breaking scaffolds at the break points resulted in pseudo-contig N50 of 108.6 Kbp and scaffold N50 of 18.7 Mb (Figure S1B). A pseudo-contig is defined by a sequence broken by gaps of >1bp, which are assumed to be gaps or errors."<br>to<br>"Breaking scaffolds at the break points resulted in a scaffold N50 length of 18.7Mbp (Figure S1). For contigs, we considered a pseudo-contig, broken at positions where 2 or more contiguous Ns appeared in scaffolds, resulting in a pseudo contig N50 of 108.6Kbp." on page 7 line 5-7.<br><br>Minorly, we changed the section title "Result" to "Data description", and the sub-section title "Sample collection and data description" to just "Sample collection".<br>---------<br><br>References<br><br>Deorowicz, S., Kokot, M., Grabowski, S., & Debudaj-Grabysz, A. (2015). KMC 2: fast and resource-frugal k-mer counting. Bioinformatics, 31(10), 1569-1576.<br><br>Warren, W. C., Hillier, L. W., Tomlinson, C., Minx, P., Kremitzki, M., Graves, T., . . . Cheng, H. H. (2017). A New Chicken Genome Assembly Provides Insight into Avian Genome Structure. G3 (Bethesda), 7(1), 109-117.
**Additional Information:**	
**Question**	**Response**
Are you submitting this manuscript to a special series or article collection?	No
**Experimental design and statistics**<br><br>Full details of the experimental design and statistical methods used should be given in the Methods section, as detailed in our Minimum Standards Reporting Checklist. Information essential to interpreting the data presented should be made available in the figure legends.<br><br>Have you included all the information requested in your manuscript?	Yes
**Resources**<br><br>A description of all resources used, including antibodies, cell lines, animals and software tools, with enough information to allow them to be uniquely identified, should be included in the Methods section. Authors are strongly encouraged to cite Research Resource Identifiers (RRIDs) for antibodies, model organisms and tools, where possible.	Yes

Powered by Editorial Manager® and ProduXion Manager® from Aries Systems Corporation

Have you included the information requested as detailed in our Minimum Standards Reporting Checklist?	
**Availability of data and materials**<br><br>All datasets and code on which the conclusions of the paper rely must be either included in your submission or deposited in publicly available repositories (where available and ethically appropriate), referencing such data using a unique identifier in the references and in the "Availability of Data and Materials" section of your manuscript.<br><br>Have you have met the above requirement as detailed in our Minimum Standards Reporting Checklist?	Yes

Powered by Editorial Manager® and ProduXion Manager® from Aries Systems Corporation

Manuscript

Click here to access/download;Manuscript;Ogye_GigaScience_20180518-

Click here to view linked References

DATA NOTE

Whole genome and transcriptome maps of the entirely black native Korean chicken breed *Yeonsan Ogye*

Jang-il Sohn[1,2*], Kyoungwoo Nam[1,*], Hyosun Hong[1,*], Jun-Mo Kim[3,*], Dajeong Lim[4], Kyung-Tai Lee[4], Yoon Jung Do[4], Chang Yeon Cho[5], Namshin Kim[6], Han-Ha Chai[4,7,§] and Jin-Wu Nam[1,2,‡]

[1]Department of Life Science, Hanyang University, Seoul 133-791,
[2]Research Institute for Convergence of Basic Sciences, Hanyang University, Seoul 133-791,
[3]Department of Animal Science and Technology, Chung-Ang University, Anseong, Gyeonggi-do, 17546, Republic of Korea.
[4]Department of Animal Biotechnology & Environment, National Institute of Animal Science, RDA, Wanju 55365,
[5]Animal Genetic Resource Research Center, National Institute of Animal Science, RDA, Namwon 55717,
[6]Personalized Genomic Medicine Research Center, KRIBB, Daejeon 34141, Republic of Korea
[7]College of Pharmacy, Chonnam National University, Kwangju 61186, Republic of Korea

* These authors contributed equally to this paper
§ hanha@korea.kr
‡ jwnam@hanyang.ac.kr

Abstract

Background: *Yeonsan Ogye* (*YO*), an indigenous Korean chicken breed (*Gallus gallus domesticus*), has entirely black external features and internal organs. In this study, the draft genome of *YO* was assembled using a hybrid *de novo* assembly method that takes advantage of high-depth Illumina short-reads (376.6X) and low-depth PacBio long-reads (9.7X). **Findings:** The contig and scaffold NG50s of the hybrid *de novo* assembly were 362.3Kbp and 16.8Mbp, respectively. The completeness (97.6%) of the draft genome (Ogye_1.1) was evaluated with single copy orthologous genes using BUSCO, and found to be comparable to the current chicken reference genome (galGal5; 97.4%; contigs were assembled with high-depth PacBio long-reads (50X) and scaffolded with short-reads), and superior to other avian genomes (92~93%; assembled with short-read-only or hybrid methods). Compared to galGal4 and 5, the draft genome included 551 structural variations including the Fibromelanosis (*FM*) locus duplication, related to hyperpigmentation. To comprehensively reconstruct transcriptome maps, RNA sequencing (RNA-seq) and reduced representation bisulfite sequencing (RRBS) data were analyzed from twenty different tissues, including four black tissues (skin, shank, comb, and fascia). The maps included 15,766 protein-coding and 6,900 long non-coding RNA genes, many of which were tissue-specifically expressed and displayed tissue-specific

DNA methylation patterns in the promoter regions. **Conclusions:** We expect that the resulting genome sequence and transcriptome maps will be valuable resources for studying domestic chicken breeds, including black-skinned chickens, as well as for understanding genomic differences between breeds and the evolution of hyperpigmented chickens and functional elements related to hyperpigmentation.

Keywords: *Gallus gallus domesticus*; *Yeonsan Ogye*; whole genome *de novo* assembly; Transcriptome maps; Hyperpigmentation

Background

The *Yeonsan Ogye* (*YO*), a designated natural monument of Korea (No. 265), is an indigenous Korean chicken breed that is notable for its entirely black plumage, skin, beak, comb, eyes, shank, claws, and internal organs [1]. In terms of its plumage and body color, as well as its number of toes, this unique chicken breed resembles the indigenous Indonesian chicken breed *Ayam cemani* [2-4]. *YO* also has some morphological features that are similar to those of the *Silkie* fowl, with the exception of the *Silkie*'s veiled black walnut comb and hair-like, fluffy plumage that is white or variably colored [5, 6]. Although the exact origin of the *YO* breed has not yet been clearly defined, its features and medicinal usages were recorded in *Dongui Bogam* [7], a traditional Korean medical encyclopedia compiled and edited by Heo Jun in 1613.

To date, a number of avian genomes from both domestic and wild species have been assembled and compared, revealing genomic signatures associated with the domestication process and genomic differences that provide an evolutionary perspective [8]. The chicken reference genome was first assembled using the *Red junglefowl* [9], first domesticated at least five thousand years ago in Asia; the latest version of the reference genome was released in 2015 (galGal5, GenBank Assembly ID GCA_000002315.3) [10]. However, because domesticated chickens exhibit diverse morphological features, including skin and plumage colors, the genome sequences of unique breeds are necessary for understanding their characteristic phenotypes through analyses of single nucleotide polymorphisms (SNPs), insertions and deletions (INDELs), structural variations (SVs), and coding and non-coding transcriptomes. Here, we provide the first version of the *YO* genome (Ogye_1.1), which includes annotations of large SVs, SNPs, INDELs, and repeats, as well as coding and non-coding transcriptome maps along with DNA methylation landscapes across twenty different *YO* tissues.

Data description

Sample collection

An 8-month-old *YO* chicken (object number: 02127), obtained from the Animal Genetic Resource Research Center of the National Institute of Animal Science (Namwon, Korea), was used in the study (**Figure 1A**). All sequencing data in this study (including data from whole genome sequencing, RNA-seq, and RRBS) were obtained from this sample bird. The protocols for the care and experimental use of *YO* were reviewed and approved by the Institutional Animal Care and Use Committee of the National Institute of Animal Science (IACUC No.: 2014-080). *YO* management, treatment, and sample collection took place at the National Institute of Animal Science.

Whole genome sequencing

Genomic DNA was extracted from blood using Wizard DNA extraction kit [35] and prepared for DNA sequencing library construction. According to the DNA fragment (insert) size, three different library types were constructed: paired-end libraries for small inserts (280 and 500bp), mate-pair libraries for large inserts (3, 5, 8, and 10Kbp), and FSMID libraries for very large inserts (40Kbp) using Illumina's protocols (Illumina, San Diego, CA, USA) (**Table 1**). The constructed libraries were sequenced using Illumina's Hiseq2000 platform. In total, 376.6X raw Illumina short-reads (100.2X from the small insert libraries and 276.4X from the large insert libraries) were generated (**Tables 1** and **S1**). To fill gaps and improve the scaffold N50, 9.7X PacBio long-reads were additionally sequenced using the PacBio RS II platform with P6C4 chemistry; the average length of the long-reads was 6Kbp (**Table 1**).

Whole transcriptome sequencing

Total RNAs were extracted from twenty different tissues using 80% EtOH and TRIzol (Sigma-Aldrich®, St. Louis, USA). The RNA concentration was checked using Quant-IT RiboGreen (Invitrogen, Carlsbad, USA). To assess the integrity of the total RNA, samples were run on the Agilent 2200 TapeStation system (Agilent Technologies, Waldbronn, Germany). Only high-quality RNA samples (RNA Integrity Number, RIN $\geq$ 7.0) were used for RNA-seq library construction. Each library was independently prepared with 300ng of total RNA using an Illumina TruSeq Stranded Total RNA Sample Prep Kit (Illumina, San

Diego, CA, USA). The rRNA in the total RNA was depleted using a Ribo-Zero kit. After rRNA depletion, the remaining RNA was purified, fragmented, and primed for cDNA synthesis. The cleaved RNA fragments were copied into the first cDNA strand using reverse transcriptase and random hexamers. This step was followed by second strand cDNA synthesis using DNA Polymerase I, RNase H, and dUTP. The resulting cDNA fragments then underwent an end repair process, the addition of a single 'A' base, after which adapters were ligated. The products were purified and enriched with PCR to create the final cDNA library. The libraries were quantified using qPCR according to the qPCR Quantification Protocol Guide (KAPA Library Quantificatoin kits for Illumina Sequencing platforms) and the integrity of the cDNA libraries was examined using the Agilent 2200 TapeStation system. In sum, about 1.5 billion RNA-seq reads were sequenced from twenty different tissues; breast, liver, bone marrow, fascia, cerebrum, gizzard, mature and immature eggs, comb, spleen, cerebellum, gall bladder, kidney, heart, uterus, pancreas, lung, skin, eye, and shank from the same bird (**Table 2**).

Reduced representation bisulfite sequencing

Preparation of reduced representation bisulfite sequencing (RRBS) libraries was done following Illumina's RRBS protocol. 5μg of genomic DNA that had been digested with the restriction enzyme MspI and purified with a QIAquick PCR purification kit (QIAGEN, Hilden, Germany) was used for library preparation, which was done using a TruSeq Nano DNA Library Prep Kit (Illumina, San Diego, USA). Eluted DNA fragments were end-repaired, extended on the 3' end with an 'A', and ligated with Truseq adapters. The products, which ranged from 175 to 225bp in length (insert DNA of 55–105bp plus adaptors of 120bp), were excised from a 2%(w/v) Low Range Ultra Agarose gel (Biorad, Hercules, USA) and purified using the QIAquick gel extraction protocol. The purified DNA underwent bisulfite conversion using an EpiTect Bisulfite Kit (Qiagen, 59104). The bisulfite-converted DNA libraries were amplified by PCR (four cycles) using PfuTurbo Cx DNA polymerase (Agilent, 600410). The quantity of the DNA libraries was then examined using qPCR and the integrity was examined using the Agilent 2200 TapeStation system. The final product was sequenced using the HiSeq™ 2500 platform (Illumina, San Diego, USA).

Ultimately, 123 million RRBS reads were produced from twenty different tissues from the same bird (see **Table 3**).

Hybrid whole genome assembly

The Ogye_1.1 genome was assembled using our hybrid genome assembly pipeline, employing the following four steps: 1) preprocessing, 2) hybrid *de novo* assembly, 3) pseudo-reference-assisted assembly, and 4) polishing and finishing (**Figures 1B** and **S1)**. In the preprocessing step, reads in which ≥ 30% of the nucleotides had a Phred score < 20 were excluded using NGS QC Toolkit (IlluQC_PRLL.pl) [36], the adaptor sequences of the remaining reads were removed using Trimmomatic (Trimmomatic, RRID:SCR_011848) [37], and three nucleotides at the 5'-end and five nucleotides at the 3'-end of the reads were trimmed using NGS QC Toolkit (TrimmingReads.pl). After quality control, the sequencing errors in the Illumina paired-end short-reads were corrected using KmerFreq and Corrector [38]. After these steps, 241.1X preprocessed reads were obtained for whole genome assembly. In turn, using the corrected short-reads, the sequencing errors in the PacBio long-reads were corrected using LoRDEC [39].

In the hybrid *de novo* genome assembly, the initial assembly (ASM1) was done with 121.2X error-corrected short-reads from the paired-end and mate-pair libraries (see **Table 1**) using ALLPATHS-LG (ALLPATHS-LG, RRID:SCR_010742) [40] with the default option, producing contigs and scaffolds with N50 lengths of 53.6Kbp and 10.7Mbp, respectively (**Figure 1B; S1**). Additionally, another assembly (ASM2) was built with 109.2X paired-end and mate-pair reads that were unused in the initial assembly (see **Table 1**) using ALLPATHS-LG, resulting in 34,539 contigs with an N50 length of 59.2Kbp. The resulting ASM2 contigs were then subjected to the pseudo-reference-assisted assembly step. In the second round of scaffolding and gap-filling (after the first scaffolding and gap-filling done during ASM1), the ASM1 scaffolds were connected with corrected PacBio long-reads using SSPACE-LongRead [41], and gaps within and between scaffolds were examined with error-corrected short-reads using GapCloser (GapCloser, RRID:SCR_015026) [38]. Then, the gap-filled scaffolds were connected again with FOSMID reads using

OPERA [42] and the remaining gaps were re-examined with error-corrected short-reads using GapCloser, resulting in scaffolds with an N50 length of 27.8Mbp. However, some mis-assemblies (as illustrated in **Figure S2A**) were found by alignment of the resulting scaffolds with the galGal4 genome (GenBank assembly accession: GCA_000002315.2) using LASTZ [43]. During an analysis of the resulting alignments, 30 mis-assemblies were detected and broken at each break point, as described in **Figure S2**. Breaking scaffolds at the break points resulted in a scaffold N50 length of 18.7Mbp (**Figure S1**). For contigs, we considered a pseudo-contig, broken at positions where 2 or more contiguous Ns appeared in scaffolds, resulting in a pseudo contig N50 of 108.6Kbp.

In the pseudo-reference-assisted assembly step, error-corrected PacBio long-reads and ASM2 contigs were utilized to reduce the topological complexity of the assembly graphs [44] (**Figure 1B**). Because even scaffolding with long-reads can be affected by repetitive sequences, the scaffolds mapped to each chromosome were transformed into a hierarchical bipartite graph to minimize the influence of repetitive sequences using TSRATOR [45] (**Figure S3**). In detail, error-corrected PacBio reads and ASM2 contigs were mapped to the scaffolds using BWA-MEM and, in turn, the scaffolds were mapped to the galGal4 genome using LASTZ to build the hierarchical bipartite graph. Using the hierarchical bipartite graphs, all scaffolds, PacBio reads, and ASM2 contigs were finally grouped to each chromosome. Based on these results, a third round of scaffolding and gap-filling was performed with the long reads and the ASM2 contigs in each chromosome group using SSPACE-LongRead and PBJelly (PBJelly, RRID:SCR_012091) [46], respectively, resulting in a scaffold N50 of 21.2Mbp with 0.85% gaps (**Figure S1**).

In the last step, nucleotide errors or ambiguities were corrected using the GATK (GATK , RRID:SCR_001876) pipeline [47] with paired-end reads, and in turn, any vector contamination was removed using VecScreen with the UniVec database [48] (**Figure 1B**), resulting in 506.3Kbp and 21.2Mbp contig and scaffold N50 lengths, respectively. The final assembly results (Ogye_1.1 scaffold) showed that the gap percentage and (pseudo-)contig N50 were significantly improved, from 1.87% and 53.6Kbp in the

initial assembly to 0.85% and 506.3Kbp in the final assembly, respectively (**Figure S1**). Using the estimated chicken genome size (1.25Gbp [10]), Ogye_1.1 scaffold's contig and scaffold NG50 lengths were estimated at 362.3Kbp and 16.8Mbp, respectively (**Figure 1C**). The complete genome sequence at the chromosome level was built by connecting the final scaffolds in their order of appearance in each chromosome with the introduction of 100Kbp 'N' gaps between them (**Figure S4**) (see *Giga*DB). To evaluate its completeness, the Ogye_1.1 genome was compared to the galGal4 (short-read-based assembly) and galGal5 (long-read-based assembly) genomes, with respect to 2,586 conserved vertebrate genes, using BUSCO (BUSCO, RRID:SCR_015008) [49] with OrthoDB v9 (OrthoDB, RRID:SCR_011980) (http://www.orthodb.org/). The Ogye_1.1 genome contained more complete single-copy BUSCO genes (**Table 4**).

Large structural variations

When the Ogye_1.1 genome was compared to galGal4 and 5 using LASTZ [43], putative large SVs (>1Kbp) were detected for each reference genome, and they were validated by four different SV prediction programs (Delly, Lumpy, FermiKit, and novoBreak) [50-53] (**Figure S5**; **Table S2**). SVs, validated by at least one program, included 185 deletions (DELs), 180 insertions (INSs), 158 duplications (DUPs), 23 inversions (INVs), and 5 intra or inter-chromosomal translocations (TRAs). 290 and 447 distinct SVs were detected relative to galGal 4 and galGal5, respectively, suggesting that either reference assembly could include mis-assemblies.

Although the Fibromelanosis (*FM*) locus, which contains the hyperpigmentation-related *edn3* gene, is known to be duplicated in the genomes of certain hyperpigmented chicken breeds, such as *Silkie* and *Ayam cemani* [3, 6], the exact structure of the duplicated *FM* locus in such breeds has not been completely resolved due to its large size (~1Mbp). A previous study, using conventional PCR assays, suggested three possible rearrangements at the *FM* locus [3]. To understand more about the mechanism of *FM* locus rearrangement in the Ogye_1.1 genome, the *FM* loci from YO and galGal4 were compared with mapped paired-end and mate-pair reads. A doubled read depth at two loci including the *FM* locus was detected in

YO, indicating that the loci had been duplicated (**Figure 2A** top). As previously reported [3, 6], our paired-end and mate-pair reads of *YO*'s *FM* locus were discordantly mapped to the galGal4 *FM* locus (**Figure S6**). The intervening region between the two duplicated regions was estimated to be 412.6Kbp in length in the Ogye_1.1 genome. Based on these results, we propose three possible scenarios that might have produced the *FM* locus rearrangement (**Figure 2B**). To discern which rearrangement best fits our results, the *FM* loci from galGal4 and the Ogye draft were compared with the resulting scaffolds, showing an inverted duplication with discontinued scaffolds at both duplicated regions (**Figures 2A; C**). The results, with a discontinued scaffold on both sides, support rearrangement 1 rather than rearrangement 2 or 3, which have a discontinued scaffold on only one side. Although rearrangement 1 needs to be further validated, the *FM* locus in the Ogye_1.1 genome was updated according to the first rearrangement (**Figure 2C**). Given the resulting alignment, the size of Gap_1 and Gap_2 were estimated to be 164.5Kbp and 63.3Kbp, respectively.

Annotations

Repeats

Repeat elements in the Ogye_1.1 and other genomes (human, mouse, pig, western painted turtle, tropical clawed frog, zebra finch, turkey, and chicken) were predicted by a reference-guided approach using RepeatMasker (RepeatMasker, RRID:SCR_012954) [54] with Repbase libraries [55]. In the Ogye_1.1 genome, 205,684 retro-transposable elements (7.65%), including long interspersed nuclear elements (LINEs; 6.41%), short interspersed nuclear elements (SINEs; 0.04%), and long terminal repeat (LTR) elements (1.20%), 27,348 DNA transposons (0.94%), 7,721 simple repeats (0.12%), and 298 low-complexity repeats (0.01%), were annotated (**Figure 3** and **Table S3**). Repeats are similarly distributed in the Ogye_1.1 and other avian genomes (**Figure 3** and **Table S4**). Compared with other avian genomes, the Ogye_1.1 genome resembles galGal4 and 5 the most in terms of repeat composition except for that of simple repeats (0.12% for Ogye_1.1, 1.12% for galGal4 and 1.24% for galGal5) and low-complexity (0.01% for Ogye_1.1, 0.24% for galGal4 and 0.25% for galGal5) and satellite DNA repeats (0.01% for Ogye_1.1,

0.20% for galGal4 and 0.22% for galGal5). The distribution of transposable elements (TEs) across all chromosomes is depicted in **Figure S7**.

SNPs/INDELs

To annotate SNPs and INDELs in the Ogye_1.1 genome, all paired-end libraries were mapped to the Ogye_1.1 genome using BWA-MEM and deduplicated using Picard modules [56]. We identified 3,206,794 SNPs and 302,463 INDELs across the genome using VarScan 2 with options --min-coverage 8 --min-reads2 2 --min-avg-qual 15 --min-var-freq 0.2 --p-value 1e-2 [57]. The densities of SNPs and INDELs across all chromosomes are depicted in **Figure S7**.

Protein-coding genes

To sensitively annotate protein-coding genes, all paired-end RNA-seq data were mapped on the Ogye_1.1 genome using STAR [58] for each tissue and the mapping results were then assembled into potential transcripts using StringTie [59]. Assembled transcripts from each sample were merged using StringTie and the resulting transcriptome was subjected to the prediction of coding DNA sequences (CDSs) using TransDecoder [60]. For high-confidence prediction, transcripts with intact gene structures (5'UTR, CDS, and 3'UTR) were selected. To verify their coding potential, the candidate sequences were examined using CPAT [61] and CPC [62]. Candidates with a high CPAT score (>0.99) were directly assigned to be protein-coding genes, and those with an intermediate score (0.8-0.99) were re-examined to determine whether the CPC score is >0. Candidates with low coding potential or that were partially annotated were examined to determine if their loci overlapped with annotated protein-coding genes from galGal4 (ENSEMBL cDNA release 85). Overlapping genes were added to the set of Ogye_1.1 protein-coding genes. Using this protein-coding gene annotation pipeline (**Figure S8**), 15,766 protein-coding genes were finally annotated in the Ogye_1.1 genome, including 946 novel genes and 14,819 known genes (**Figure 4A**). However, 164 galGal4 protein-coding genes were not mapped to the Ogye_1.1 genome by GMAP (**Table S5**), 131 of which were confirmed to be expressed in *YO* (≥ 0.1 FPKM) using all paired-end *YO* RNA-seq

data. In contrast, the remaining 33 genes were not expressed in *YO* (< 0.1 FPKM) or were lost from the Ogye_1.1 genome. Of the 33 missing genes, 26 appeared to be located on unknown chromosomes and the remainder are on autosomes (six genes) or the W sex chromosome (one gene) in galGal4. The density of protein-coding genes across all chromosomes is depicted in **Figure S7**.

lncRNAs

To annotate and profile lncRNA genes, we used our lncRNA annotation pipeline (**Figure S9**), adopted from our previous study [63]. Pooled single- and paired-end RNA-seq reads from each tissue were mapped to the Ogye_1.1 genome (PRJNA412424) using STAR [58], and subjected to transcriptome assembly using Cufflinks (Cufflinks, RRID:SCR_014597) [64], leading to the construction of transcriptome maps for twenty tissues. The resulting maps were combined by Cuffmerge and, in total, 206,084 transcripts from 103,405 loci were reconstructed in the Ogye genome. We removed other RNA biotypes (the sequences of mRNAs, tRNAs, rRNAs, snoRNAs, miRNAs, and other small non-coding RNAs downloaded from ENSEMBL biomart) and short transcripts (less than 200nt in length). 54,760 lncRNA candidate loci (60,257 transcripts) were retained and compared with a chicken lncRNA annotation from NONCODE (v2016) [65]. Of the candidates, 2,094 loci (5,215 transcripts) overlapped with previously annotated chicken lncRNAs. 52,666 non-overlapping loci (55,042 transcripts) were further examined to determine whether they had coding potential using CPC score [62]. Those with a score greater than -1 were filtered out, and the remainder (14,108 novel lncRNA candidate loci without coding potential) were subjected to the next step. Because many candidates still appeared to be fragmented, those with a single exon but with neighboring candidates within 36,873bp, which is the length of introns in the 99th percentile, were re-examined using both exon-junction reads consistently presented over twenty tissues and the maximum entropy score [66], as done in our previous study [63]. If there were at least two junction reads spanning two neighboring transcripts or if the entropy score was greater than 4.66 in the interspace, the two candidates were reconnected, and those with a single exon were discarded. In the final version, 6,900 loci (5,610 novel and 1,290 known) were annotated as lncRNAs (see **Figure 4B**), which included 6,170

(89.40 %) intergenic lncRNAs and 730 (10.57 %) anti-sense ncRNAs. Consistent with previous results [67-70], the median Ogye lncRNA transcript length and exon number were less than those of protein-coding genes (**Figure 4C** and **D**).

Whereas 13,540 of 14,983 protein-coding genes (90.4%) were redetected in our protein-coding gene annotations (see **Figure 4A**) only 1,290 (13.6%) of NONCODE lncRNAs were redetected in our Ogye_1.1 lncRNA annotations (**Figure 4B**). The majority of the missing NONCODE lncRNAs were either fragments of protein-coding genes or not expressed in all twenty Ogye tissues (**Figure 4B**). Only 276 were actually missing in the transcriptome assembly and 648 were not mapped to the Ogye_1.1 genome.

Coding and non-coding transcriptome maps

Using paired-end *YO* RNA-seq data, the expression levels of protein-coding and lncRNA genes were calculated across twenty tissues (**Figure S10**). In the profiled transcriptomes, 1,814 protein-coding and 1,226 lncRNA genes were expressed with ≥ 10 FPKM in only one tissue, whereas 1,559 protein-coding and 351 lncRNA genes were expressed with ≥ 10 FPKM in all tissues. In black tissues (fascia, comb, skin, and shank), we have found that 6,702 protein-coding and 3,291 lncRNA genes were expressed with ≥ 10 FPKM, the majority of which appeared to be expressed in tissue-specific manner (**Figure 5A**). For instance, the protein-coding gene *krt9* and the lncRNA *lnc-lama2-1* are highly expressed in black tissues, particularly in comb and shank, respectively (**Figure 5B** and **C**).

Because lncRNAs tend to be specifically expressed in a tissue or in related tissues, they could be more useful than protein-coding genes for defining genomic characteristics of tissues. To prove this idea, principle component analyses (PCAs) were performed with 9,153 tissue-specific protein-coding and 5,191 tissue-specific lncRNA genes using the reshape2 R package (**Figure 5D** and **E**) [71]. Here, we classified a gene as tissue-specific if the maximum expression value was at least four-fold higher than the mean value over twenty tissues. As expected, the 1st, 2nd, and 3rd PCs of lncRNAs enabled us to predict the majority of variances, and better discerned distantly-related tissues and functionally and histologically-related tissues

(*i.e.*, black tissues and brain tissues) (**Figure 5E**) than those of protein-coding genes (**Figure 5C**).

DNA methylation maps

After mapping RRBS reads to the Ogye_1.1 genome (**Table 3**), DNA methylation signals (C to T changes in CpGs) were calculated across chromosomes using Bismark [72]. Of all CpG sites in the genome, 31~65% were methylated across tissues whereas only 19~43% were methylated in gene promoters (the region 2Kbp upstream of the transcription start site (TSS)) (**Table 5**), indicating that the promoters of expressed genes tended to be hypomethylated. The DNA methylation landscapes in the regions 2Kbp upstream of the protein-coding and lncRNA gene TSSs are shown in **Figure S11**. Based on the CpG methylation pattern, hierarchical clustering was performed using the rsgcc R package, and clusters including adjacent or functionally related tissues, such as cerebrum and cerebellum, immature and mature eggs, or comb and skin were identified (**Figure 6A**).

We then examined the average methylation landscapes over protein-coding and lncRNA loci to check whether the CpG methylation profiles were properly processed. As previously shown [73-76], the average methylation levels in gene body regions were much higher than those in promoters across tissues (**Figure 6B** and **C**). To investigate the association between CpG methylation in the promoter and target gene expression, the average methylation levels of tissue-specific genes (280 protein-coding and 392 lncRNA genes with expression ≥ 10 FPKM in at least one tissue and with a maximum expression value four-fold higher than the mean expression level in twenty tissues) were compared to those of others expressed in their specific tissues. The methylation levels of highly expressed genes appeared to be lower than those of others (**Figure 6D** and **E**). We then searched for genes with tissue-specific expression that was significantly correlated to the promoter methylation level using Spearman's correlation method (**Figure 6F**). To exclude stochastic noise, only tissues in which a certain position had a sufficient number of reads (at least five) were taken into account for measuring the correlation. We found that the expression levels of 454 protein-coding and 25 lncRNA genes displayed a negative correlation to promoter methylation

levels, whereas 157 protein-coding and 20 lncRNA genes had a positive correlation (box plots in **Figure 6F**).

Discussion

In this work, the first draft genome of *YO*, Ogye_1.1, was constructed with genomic variation, repeat, and protein-coding and non-coding gene maps. Compared with the chicken reference genome maps, many more novel coding and non-coding elements were identified from large-scale RNA-seq datasets across twenty different tissues. Although the Ogye_1.1 genome is comparable with galGal5 with respect to genome completeness evaluated by BUSCO, Ogye_1.1 seems to lack simple and long repeats compared with galGal5, which was assembled from high-depth PacBio long-reads (50X) that can capture simple and long repeats. Although PacBio long-reads were also produced in our study, they were only used for scaffolding and gap-filling because of their shallow depth (9.7X), probably resulting in some simple and satellite repeats being missed in Ogye_1.1. A similar tendency can be seen in the Golden-collared manakin genome (ASM171598v1) [32] (**Figure 3**) and the gray mouse lemur genome (Mmur3.0) [77], which were also assembled in a hybrid manner with high-depth Illumina short-reads and low-depth PacBio long-reads.

15,766 protein-coding and 6,900 lncRNA genes were annotated from twenty *YO* tissues. 946 novel protein-coding genes were identified, while 164 *Galllus gallus red junglefowl* genes were missed in our annotations. In the case of lncRNAs, only about 13.6% of previously annotated chicken lncRNAs were redetected, and the remainder were mostly not expressed in *YO* or were false annotations, suggesting that the current chicken lncRNA annotations should be carefully examined. Our Ogye lncRNAs resembled previously annotated mammalian lncRNAs in their genomic characteristics, including transcript length, exon number, and tissue-specific expression patterns, providing evidence for the accuracy of the new annotations. Hence, our lncRNA catalogue may help us improve lncRNA annotations in the chicken reference genome.

Availability of data

All of our sequence data and the genome sequence have been deposited in NCBI's Gene Expression Omnibus (GEO) superseries GSE 104358 and BioProject PRJNA412408. All supporting data (genome and gene sequence files, the expression tables for protein-coding and lncRNA genes, and the RRBS, protein-coding, lncRNA, SNP, and INDEL annotation files) are available in the *GigaScience* repository *Giga*DB [78].

Additional files

Additional file 1: Supplementary Figures and Tables

Additional file 2: Description (README) of available data in *Giga*DB

Additional file 3: Command lines of programs and pipelines with run-time options used in this study

Acknowledgements

We thank all members of the BIG lab for helpful comments and discussions. This work was supported by the Cooperative Research Program for Agriculture Science and Technology Development (Project title: National Agricultural Genome Program, Project No. PJ01045301 and PJ01045303).

Authors' contributions

KTL, NSK, HHC, and JWN designed the study, KTL, YJD, and CYC collected samples, DJL, HHC, and KTL collected sequencing data, and JIS, KWN, NSK, JMK, HHC, and JMN performed the analysis and developed the methodology. JIS, KWN, JMK, and JWN wrote the manuscript.

Competing interests

The authors declare that they have no competing interests.

Tables

Table 1. Summary of whole genome sequencing data (estimated genome size 1.25Gbp)

Table 2. Sequencing and mapping summary of RNA-seq data

Table 3. Sequencing and mapping summary of RRBS data

Table 4. Comparison of genome completeness using BUSCO

Table 5. Summary of methylated CpG sites across twenty tissues

Figure legends

Figure 1. A. A photograph of *Yeonsan Ogye* (*YO*), taken before sampling; **B.** Hybrid genome assembly pipeline comprising four steps, each of which utilizes a different set of sequencing reads (see **Table 1**). Detailed methods for breaking mis-assembly and pseudo-reference-assisted assembly are depicted in Figures **S2** and **S3**; **C.** The NG50 and average length of pseudo-contigs and scaffolds for the Ogye_1.1 and other avian genomes, generated using the indicated assembly methods (in the last column, sequencing platforms are designated as follows: "I" indicates Illumina, "P" is PacBio, "S" is Sanger, and "4" is Roche454).

Figure 2. A. The read depth of a locus on chromosome 20 is shown in the top panel and the continuous/discontinuous patterns between mapped scaffolds are shown in the bottom panel. The star indicates the pattern that is discontinuous on both sides, validated in **C**; **B.** Three possible scenarios were developed based on the overlap patterns. The green and red lines indicated two duplicated genomic loci (Dupl_1 and Dupl_2, respectively) including the *FM* locus. Scenario 1 consists of a one-step rearrangement—an inverted duplication—whereas Scenarios 2 and 3 consist of a simultaneous rearrangement of an inverted duplication and an inversion. The three rearrangements were suggested in a previous study [3]; **C.** A comparison of the *FM* locus in galGal4 and the Ogye draft genome with aligned contigs (black lines) in each scaffold. The gray bands indicate the estimated gaps between contigs. The estimated sizes of Gap_1 and Gap_2 are 164.5Kbp and 63.3Kbp, respectively. The purple lines in the box indicate the *edn3* gene locus and the green and yellow shades indicate the duplicated regions (Dupl_1 and Dupl_2, respectively). The dark green and yellow shades indicate the discontinuous regions between scaffolds.

Figure 3. Composition of repeat elements in different assemblies of avian, amphibian, reptile, and mammalian genomes. The repeats in unplaced scaffolds were not considered.

Figure 4. A. A Venn diagram showing the number of protein-coding genes in the Ogye_1.1 genome**; B.** A Venn diagram showing the number of Ogye_1.1 and galGal4 lncRNAs; **C.** Distribution of transcript length (red for lncRNAs and cyan for protein-coding genes). The vertical dotted lines indicate the median length; **D.** Distribution of the number of exons per transcript. Otherwise, as in **C**.

Figure 5. A. The expression patterns of the genes expressed with ≥ 10 FPKM in black tissues. Expression levels are indicated with a color-coded Z-score (red for low and blue for high expression) as shown in the key; **B**. Expression levels of *krt9* across twenty tissues; **C.** Expression levels of *lnc-lama2-1* across twenty tissues; **D.** Principal component analysis (PCA) using tissue-specific protein-coding genes. PCs explaining the variances are indicated with the amount of the contribution in the top-left plot. PCA plots are shown with PC1, PC2, and PC3 plotted in a pairwise manner. Each tissue is indicated on the PCA plot with a specific color; **E.** PCA using tissue-specific lncRNAs. Otherwise, as in **A**.

Figure 6. A. Hierarchical clustering using Pearson's correlation of DNA methylation patterns between tissues; **B-C.** Average DNA methylation landscapes along protein-coding (**B**) and lncRNA (**C**) gene bodies and their flanking regions across twenty tissues; **D-E.** Average DNA methylation levels of protein-coding (**D**) and lncRNA (**E**) genes in the tissue of maximum expression (red) and the other tissues (blue); **F.** Spearman's correlation coefficients between gene expression and promoter methylation levels are shown across chromosomes (heatmaps) in a Circos plot. The bar charts indicate the number of genes (left for protein-coding genes and right for lncRNAs) with significant negative (red) and positive (cyan) correlations ($P < 0.05$) between their promoter methylation levels and their expression values.

References

1. Domestic Animal Diversity Information System. http://dad.fao.org/.
2. Dorshorst B, Okimoto R and Ashwell C. Genomic regions associated with dermal hyperpigmentation, polydactyly and other morphological traits in the Silkie chicken. J Hered. 2010;101 3:339-50. doi:10.1093/jhered/esp120.
3. Dorshorst B, Molin AM, Rubin CJ, Johansson AM, Stromstedt L, Pham MH, et al. A complex genomic rearrangement involving the endothelin 3 locus causes dermal hyperpigmentation in the chicken. PLoS Genet. 2011;7 12:e1002412. doi:10.1371/journal.pgen.1002412.
4. Arora G, Mishra SK, Nautiyal B, Pratap SO, Gupta A, Beura CK, et al. Genetics of hyperpigmentation associated with the Fibromelanosis gene (Fm) and analysis of growth and meat quality traits in crosses of native Indian Kadaknath chickens and non-indigenous breeds. Br Poult Sci. 2011;52 6:675-85. doi:10.1080/00071668.2011.635637.
5. Łukasiewicz M, Niemiec J, Wnuk A and Mroczek-Sosnowska N. Meat quality and the histological structure of breast and leg muscles in Ayam Cemani chickens, Ayam Cemani× Sussex hybrids and slow-growing Hubbard JA 957 chickens. Journal of the Science of Food and Agriculture. 2015;95 8:1730-5.
6. Dharmayanthi AB, Terai Y, Sulandari S, Zein MS, Akiyama T and Satta Y. The origin and evolution of fibromelanosis in domesticated chickens: Genomic comparison of Indonesian Cemani and Chinese Silkie breeds. PLoS One. 2017;12 4:e0173147. doi:10.1371/journal.pone.0173147.
7. It has been registered on UNESCO's Memory of the World Programme in 2009. http://www.unesco.org/new/en/communication-and-information/memory-of-the-world/.
8. Zhang G, Li C, Li Q, Li B, Larkin DM, Lee C, et al. Comparative genomics reveals insights into avian genome evolution and adaptation. Science. 2014;346 6215:1311-20. doi:10.1126/science.1251385.
9. International Chicken Genome Sequencing C. Sequence and comparative analysis of the chicken genome provide unique perspectives on vertebrate evolution. Nature. 2004;432 7018:695-716. doi:10.1038/nature03154.
10. Warren WC, Hillier LW, Tomlinson C, Minx P, Kremitzki M, Graves T, et al. A New Chicken Genome Assembly Provides Insight into Avian Genome Structure. G3 (Bethesda). 2017;7 1:109-17. doi:10.1534/g3.116.035923.
11. Warren WC, Clayton DF, Ellegren H, Arnold AP, Hillier LW, Kunstner A, et al. The genome of a songbird. Nature. 2010;464 7289:757-62. doi:10.1038/nature08819.
12. Animal genome size database (release 2.0). http://www.genomesize.com/.
13. Dalloul RA, Long JA, Zimin AV, Aslam L, Beal K, Blomberg LA, et al. Multi-platform next-generation sequencing of the domestic turkey (Meleagris gallopavo): genome assembly and analysis. PLoS biology. 2010;8 9:e1000475.
14. Krishan A, Dandekar P, Nathan N, Hamelik R, Miller C and Shaw J. DNA index, genome size, and electronic nuclear volume of vertebrates from the Miami Metro Zoo. Cytometry A. 2005;65 1:26-34. doi:10.1002/cyto.a.20130.
15. Poelstra JW, Vijay N, Bossu CM, Lantz H, Ryll B, Muller I, et al. The genomic landscape underlying phenotypic integrity in the face of gene flow in crows. Science. 2014;344 6190:1410-4. doi:10.1126/science.1253226.

16. Doyle JM, Katzner TE, Bloom PH, Ji Y, Wijayawardena BK and DeWoody JA. The genome sequence of a widespread apex predator, the golden eagle (Aquila chrysaetos). PLoS One. 2014;9 4:e95599. doi:10.1371/journal.pone.0095599.
17. Zhang G, Parker P, Li B, Li H and Wang J. The genome of Darwin's Finch (Geospiza fortis). 2012.
18. Lepidothrix coronata (blue-crowned manakin). https://www.ncbi.nlm.nih.gov/genome/?term=Blue-crowned%20manakin.
19. Tuttle EM, Bergland AO, Korody ML, Brewer MS, Newhouse DJ, Minx P, et al. Divergence and Functional Degradation of a Sex Chromosome-like Supergene. Curr Biol. 2016;26 3:344-50. doi:10.1016/j.cub.2015.11.069.
20. Andrews CB, Mackenzie SA and Gregory TR. Genome size and wing parameters in passerine birds. Proc Biol Sci. 2009;276 1654:55-61. doi:10.1098/rspb.2008.1012.
21. Cornetti L, Valente LM, Dunning LT, Quan X, Black RA, Hébert O, et al. The genome of the "great speciator" provides insights into bird diversification. Genome biology and evolution. 2015;7 9:2680-91.
22. Qu Y, Zhao H, Han N, Zhou G, Song G, Gao B, et al. Ground tit genome reveals avian adaptation to living at high altitudes in the Tibetan plateau. Nature communications. 2013;4:2071.
23. Li S, Li B, Cheng C, Xiong Z, Liu Q, Lai J, et al. Genomic signatures of near-extinction and rebirth of the crested ibis and other endangered bird species. Genome Biol. 2014;15 12:557. doi:10.1186/s13059-014-0557-1.
24. Warren W, Jarvis ED, Wilson RK, Howard JT, Gilbert MTP, Zhang G, et al. Genomic data of the Bald Eagle (Haliaeetus leucocephalus). 2014.
25. Zhang G, Li B, Li C, Gilbert MTP, Jarvis E, Consortium TAG, et al. Genomic data of the American Crow (Corvus brachyrhynchos). 2014.
26. Zhan XJ, Pan SK, Wang JY, Dixon A, He J, Muller MG, et al. Peregrine and saker falcon genome sequences provide insights into evolution of a predatory lifestyle. Nature Genetics. 2013;45 5:563-U142. doi:10.1038/ng.2588.
27. Shapiro MD, Kronenberg Z, Li C, Domyan ET, Pan H, Campbell M, et al. Genomic diversity and evolution of the head crest in the rock pigeon. Science. 2013;339 6123:1063-7. doi:10.1126/science.1230422.
28. Ganapathy G, Howard JT, Ward JM, Li J, Li B, Li Y, et al. High-coverage sequencing and annotated assemblies of the budgerigar genome. Gigascience. 2014;3 1:11. doi:10.1186/2047-217X-3-11.
29. Andrews CB and Gregory TR. Genome size is inversely correlated with relative brain size in parrots and cockatoos. Genome. 2009;52 3:261-7. doi:10.1139/G09-003.
30. Zhang G, Li B, Li C, Gilbert MTP, Jarvis E, Consortium TAG, et al. Genomic data of the Little egret (Egretta garzetta). 2014.
31. Zhang G, Li B, Li C, Gilbert MTP, Houde P, Jarvis ED, et al. Genomic data of the Hoatzin (Opisthocomus hoazin). 2014.
32. Zhang G, Li B, Li C, Gilbert MTP, Schlinger B, Jarvis ED, et al. Genomic data of the Golden-collared Manakin (Manacus vitellinus). 2014.
33. Deorowicz S, Kokot M, Grabowski S and Debudaj-Grabysz A. KMC 2: fast and resource-frugal k-mer counting. Bioinformatics. 2015;31 10:1569-76. doi:10.1093/bioinformatics/btv022.

34. Earl D, Bradnam K, St John J, Darling A, Lin D, Fass J, et al. Assemblathon 1: a competitive assessment of de novo short read assembly methods. Genome Res. 2011;21 12:2224-41. doi:10.1101/gr.126599.111.
35. Miller SA, Dykes DD and Polesky HF. A simple salting out procedure for extracting DNA from human nucleated cells. Nucleic Acids Res. 1988;16 3:1215.
36. Patel RK and Jain M. NGS QC Toolkit: a toolkit for quality control of next generation sequencing data. PloS one. 2012;7 2:e30619.
37. Bolger AM, Lohse M and Usadel B. Trimmomatic: a flexible trimmer for Illumina sequence data. Bioinformatics. 2014;30 15:2114-20.
38. Luo R, Liu B, Xie Y, Li Z, Huang W, Yuan J, et al. SOAPdenovo2: an empirically improved memory-efficient short-read de novo assembler. Gigascience. 2012;1 1:18. doi:10.1186/2047-217X-1-18.
39. Salmela L and Rivals E. LoRDEC: accurate and efficient long read error correction. Bioinformatics. 2014;30 24:3506-14. doi:10.1093/bioinformatics/btu538.
40. Gnerre S, Maccallum I, Przybylski D, Ribeiro FJ, Burton JN, Walker BJ, et al. High-quality draft assemblies of mammalian genomes from massively parallel sequence data. Proc Natl Acad Sci U S A. 2011;108 4:1513-8. doi:10.1073/pnas.1017351108.
41. Boetzer M and Pirovano W. SSPACE-LongRead: scaffolding bacterial draft genomes using long read sequence information. BMC Bioinformatics. 2014;15 1:211. doi:10.1186/1471-2105-15-211.
42. Gao S, Sung WK and Nagarajan N. Opera: reconstructing optimal genomic scaffolds with high-throughput paired-end sequences. J Comput Biol. 2011;18 11:1681-91. doi:10.1089/cmb.2011.0170.
43. Harris R. *Improved pairwise alignment of genomic DNA*. PhD Thesis, 2007.
44. Sohn JI and Nam JW. The present and future of de novo whole-genome assembly. Brief Bioinform. 2018;19 1:23-40. doi:10.1093/bib/bbw096.
45. TSRATOR. https://github.com/sohnjangil/tsrator.git.
46. English AC, Richards S, Han Y, Wang M, Vee V, Qu J, et al. Mind the gap: upgrading genomes with Pacific Biosciences RS long-read sequencing technology. PLoS One. 2012;7 11:e47768. doi:10.1371/journal.pone.0047768.
47. McKenna A, Hanna M, Banks E, Sivachenko A, Cibulskis K, Kernytsky A, et al. The Genome Analysis Toolkit: a MapReduce framework for analyzing next-generation DNA sequencing data. Genome Res. 2010;20 9:1297-303. doi:10.1101/gr.107524.110.
48. VecScreen https://anonsvn.ncbi.nlm.nih.gov/repos/v1/trunk/c++/ and UniVec database https://www.ncbi.nlm.nih.gov/tools/vecscreen/univec/.
49. Simao FA, Waterhouse RM, Ioannidis P, Kriventseva EV and Zdobnov EM. BUSCO: assessing genome assembly and annotation completeness with single-copy orthologs. Bioinformatics. 2015;31 19:3210-2. doi:10.1093/bioinformatics/btv351.
50. Rausch T, Zichner T, Schlattl A, Stutz AM, Benes V and Korbel JO. DELLY: structural variant discovery by integrated paired-end and split-read analysis. Bioinformatics. 2012;28 18:i333-i9. doi:10.1093/bioinformatics/bts378.
51. Layer RM, Chiang C, Quinlan AR and Hall IM. LUMPY: a probabilistic framework for structural variant discovery. Genome Biol. 2014;15 6:R84. doi:10.1186/gb-2014-15-6-r84.
52. Li H. FermiKit: assembly-based variant calling for Illumina resequencing data. Bioinformatics. 2015;31 22:3694-6. doi:10.1093/bioinformatics/btv440.

53. Chong Z, Ruan J, Gao M, Zhou W, Chen T, Fan X, et al. novoBreak: local assembly for breakpoint detection in cancer genomes. Nat Methods. 2017;14 1:65-7. doi:10.1038/nmeth.4084.
54. Tempel S. Using and understanding RepeatMasker. Mobile Genetic Elements: Protocols and Genomic Applications. 2012:29-51.
55. Bao W, Kojima KK and Kohany O. Repbase Update, a database of repetitive elements in eukaryotic genomes. Mob DNA. 2015;6 1:11. doi:10.1186/s13100-015-0041-9.
56. Picard Tools. http://broadinstitute.github.io/picard/.
57. Koboldt DC, Zhang Q, Larson DE, Shen D, McLellan MD, Lin L, et al. VarScan 2: somatic mutation and copy number alteration discovery in cancer by exome sequencing. Genome Res. 2012;22 3:568-76. doi:10.1101/gr.129684.111.
58. Dobin A, Davis CA, Schlesinger F, Drenkow J, Zaleski C, Jha S, et al. STAR: ultrafast universal RNA-seq aligner. Bioinformatics. 2013;29 1:15-21. doi:10.1093/bioinformatics/bts635.
59. Pertea M, Pertea GM, Antonescu CM, Chang TC, Mendell JT and Salzberg SL. StringTie enables improved reconstruction of a transcriptome from RNA-seq reads. Nat Biotechnol. 2015;33 3:290-5. doi:10.1038/nbt.3122.
60. TransDecoder. https://github.com/TransDecoder/TransDecoder/.
61. Wang L, Park HJ, Dasari S, Wang S, Kocher JP and Li W. CPAT: Coding-Potential Assessment Tool using an alignment-free logistic regression model. Nucleic Acids Res. 2013;41 6:e74. doi:10.1093/nar/gkt006.
62. Kong L, Zhang Y, Ye ZQ, Liu XQ, Zhao SQ, Wei L, et al. CPC: assess the protein-coding potential of transcripts using sequence features and support vector machine. Nucleic Acids Res. 2007;35 Web Server issue:W345-9. doi:10.1093/nar/gkm391.
63. You BH, Yoon SH and Nam JW. High-confidence coding and noncoding transcriptome maps. Genome Res. 2017;27 6:1050-62. doi:10.1101/gr.214288.116.
64. Trapnell C, Williams BA, Pertea G, Mortazavi A, Kwan G, van Baren MJ, et al. Transcript assembly and quantification by RNA-Seq reveals unannotated transcripts and isoform switching during cell differentiation. Nat Biotechnol. 2010;28 5:511-5. doi:10.1038/nbt.1621.
65. Zhao Y, Li H, Fang S, Kang Y, Wu W, Hao Y, et al. NONCODE 2016: an informative and valuable data source of long non-coding RNAs. Nucleic Acids Res. 2016;44 D1:D203-8. doi:10.1093/nar/gkv1252.
66. Yeo G and Burge CB. Maximum entropy modeling of short sequence motifs with applications to RNA splicing signals. J Comput Biol. 2004;11 2-3:377-94. doi:10.1089/1066527041410418.
67. Pauli A, Valen E, Lin MF, Garber M, Vastenhouw NL, Levin JZ, et al. Systematic identification of long noncoding RNAs expressed during zebrafish embryogenesis. Genome Res. 2012;22 3:577-91. doi:10.1101/gr.133009.111.
68. Weikard R, Hadlich F and Kuehn C. Identification of novel transcripts and noncoding RNAs in bovine skin by deep next generation sequencing. BMC Genomics. 2013;14 1:789. doi:10.1186/1471-2164-14-789.
69. Billerey C, Boussaha M, Esquerre D, Rebours E, Djari A, Meersseman C, et al. Identification of large intergenic non-coding RNAs in bovine muscle using next-generation transcriptomic sequencing. BMC Genomics. 2014;15 1:499. doi:10.1186/1471-2164-15-499.

70. Al-Tobasei R, Paneru B and Salem M. Genome-Wide Discovery of Long Non-Coding RNAs in Rainbow Trout. PLoS One. 2016;11 2:e0148940. doi:10.1371/journal.pone.0148940.
71. reshape2. https://github.com/hadley/reshape.
72. Krueger F and Andrews SR. Bismark: a flexible aligner and methylation caller for Bisulfite-Seq applications. Bioinformatics. 2011;27 11:1571-2. doi:10.1093/bioinformatics/btr167.
73. Laurent L, Wong E, Li G, Huynh T, Tsirigos A, Ong CT, et al. Dynamic changes in the human methylome during differentiation. Genome Res. 2010;20 3:320-31. doi:10.1101/gr.101907.109.
74. Huang YZ, Sun JJ, Zhang LZ, Li CJ, Womack JE, Li ZJ, et al. Genome-wide DNA methylation profiles and their relationships with mRNA and the microRNA transcriptome in bovine muscle tissue (Bos taurine). Sci Rep. 2014;4:6546. doi:10.1038/srep06546.
75. Laine VN, Gossmann TI, Schachtschneider KM, Garroway CJ, Madsen O, Verhoeven KJ, et al. Evolutionary signals of selection on cognition from the great tit genome and methylome. Nat Commun. 2016;7:10474. doi:10.1038/ncomms10474.
76. Li A, Zhou ZY, Hei X, Otecko NO, Zhang J, Liu Y, et al. Genome-wide discovery of long intergenic noncoding RNAs and their epigenetic signatures in the rat. Sci Rep. 2017;7 1:14817. doi:10.1038/s41598-017-13844-9.
77. Larsen PA, Harris RA, Liu Y, Murali SC, Campbell CR, Brown AD, et al. Hybrid de novo genome assembly and centromere characterization of the gray mouse lemur (Microcebus murinus). BMC Biol. 2017;15 1:110. doi:10.1186/s12915-017-0439-6.
78. Sohn J, Nam K, Hong H, Kim J, Lim D, Lee K et al. Supporting data for "Whole genome and transcriptome maps of the entirely black native Korean chicken breed Yeonsan Ogye" *GigaScience* Database 2018. http://dx.doi.org/10.5524/100467

Click here to access/download;Table;Table1.docx

Table 1. Summary of whole genome sequencing data (estimated genome size 1.25Gbp)

Platform	Library type	Insert-size	Raw data: No. of read (10^6)	Raw data: Total base (Gbp)	Raw data: Coverage (X)	Raw data: SRA accession	Preprocessed data: Coverage (X)	Usage of data† (coverage, X): SEC	ASM1	ASM2	SCF	GF	SV	SIC
Illumina HiSeq 2000	Paired-end	280 bp	259.2	39.0	31.2	SRR6189087	21.4	O	O				O	O
			248.9	37.4	29.9	SRR6189084	20.5	O		O		O	O	O
		500 bp	87.1	13.1	10.5	SRR6189095	4.8	O	O				O	O
			94.4	14.2	11.4	SRR6189097	5.2	O	O			O	O	O
			28.1	4.2	3.4	SRR6189096	1.3	O	O				O	O
			28.3	4.3	3.4	SRR6189098	1.2	O		O		O	O	O
			29.2	4.4	3.5	SRR6189082	1.8	O		O			O	O
			57.4	8.6	6.9	SRR6189094	4.5	O	O			O	O	O
	Paired-end total		832.5	125.2	100.2		60.7	60.7	37.2	23.5	–	31.4	60.7	60.7
	Mate-pair	3Kbp	293.1	43.6	34.9	SRR6189093	23.6			O			O	
			270.0	40.2	32.1	SRR6189083	21.6		O					
		5Kbp	229.6	34.2	27.4	SRR6189081	16.9		O				O	
			212.8	31.7	25.4	SRR6189088	15.7			O				
		8Kbp	273.1	40.7	32.6	SRR6189085	20.2		O				O	
			270.5	40.4	32.3	SRR6189086	19.7			O				
		10Kbp	338.2	50.4	40.3	SRR6189091	26.7			O			O	
			315.9	47.1	37.7	SRR6189092	25.3		O					
		40Kbp&	169.9	17.2	13.7	SRR6189089	10.7				O			
	Mate-pair total		2,373.2	345.5	276.4		180.4	–	84.0	85.7	10.7	–	87.4	–
PacBio RS II	Long-read	6Kbp*	1.7	12.1	9.7	SRR6189090	9.3				O	O		
Illumina total			3,205.7	470.7	376.6		241.1	60.7	121.2	109.2	20.0	40.7	148.1	
Illumina + PacBio total			3,207.4	482.8	386.3		250.4	60.7	121.2	109.2	29.3	50.0	148.1	

* Average read length.
& Fosmid.
† Abbreviations: SEC (sequencing error correction), ASM1 (initial ALLPATHS-LG assembly), ASM2 (additional ALLPATHS-LG assembly), SCF (scaffolding), GF (gap-filling), SV (structural variation detection), and SIC (SNP/INDEL calling).

Table 2. Sequencing and mapping summary of RNA-seq data

Samples	Paired-end			Single-end		
	No. of reads	Mapping rate	SRA accession	No. of reads	Mapping rate	SRA accession
Breast	34,893,064	92.05%	SRX3223583	43,294,022	90.70%	SRX3223603
Liver	33,476,266	85.75%	SRX3223584	48,032,813	85.81%	SRX3223604
Bone marrow	30,975,506	85.00%	SRX3223585	40,286,974	87.99%	SRX3223605
Fascia	33,316,764	84.61%	SRX3223586	42,425,452	87.93%	SRX3223606
Cerebrum	30,887,821	89.95%	SRX3223587	46,455,658	92.32%	SRX3223607
Gizzard	31,537,118	84.00%	SRX3223588	38,689,871	85.82%	SRX3223608
Immature egg	32,009,437	87.73%	SRX3223589	32,048,703	87.80%	SRX3223609
Comb	31,936,332	85.34%	SRX3223590	37,985,049	87.76%	SRX3223610
Spleen	28,946,777	89.70%	SRX3223591	38,704,448	89.33%	SRX3223611
Mature egg	30,873,699	91.98%	SRX3223592	40,650,664	92.17%	SRX3223612
Cerebellum	30,798,145	93.53%	SRX3223593	39,940,946	93.34%	SRX3223613
Gall bladder	35,862,229	84.83%	SRX3223594	35,423,339	87.06%	SRX3223614
Kidney	29,953,007	87.25%	SRX3223595	39,894,009	89.99%	SRX3223615
Heart	30,986,431	94.14%	SRX3223596	45,951,338	91.49%	SRX3223616
Uterus	33,444,002	91.89%	SRX3223597	46,650,355	90.63%	SRX3223617
Pancreas	30,595,568	82.52%	SRX3223598	47,361,192	84.35%	SRX3223618
Lung	31,533,498	87.63%	SRX3223599	45,552,982	92.34%	SRX3223619
Skin	34,442,464	82.36%	SRX3223600	41,934,970	84.00%	SRX3223620
Eye	33,006,509	89.21%	SRX3223601	44,044,630	91.82%	SRX3223621
Shank	28,643,334	94.07%	SRX3223602	47,716,995	79.86%	SRX3223622

 Click here to access/download;Table;Table3.docx

Table 3. Sequencing and mapping summary of RRBS data

Samples	No. of reads	Mapping rate	SRA accession
Breast	6,042,106	68.90%	SRX3223667
Liver	6,744,208	74.20%	SRX3223668
Bone marrow	5,736,011	72.00%	SRX3223669
Fascia	5,720,194	68.90%	SRX3223670
Cerebrum	6,078,989	70.00%	SRX3223671
Gizzard	5,731,878	69.40%	SRX3223672
Immature egg	6,741,258	67.70%	SRX3223673
Comb	5,948,687	72.90%	SRX3223674
Spleen	6,307,517	77.60%	SRX3223675
Mature egg	6,246,607	69.20%	SRX3223676
Cerebellum	6,291,610	68.20%	SRX3223677
Gall bladder	5,738,180	70.10%	SRX3223678
Kidney	5,470,502	68.60%	SRX3223679
Heart	5,462,739	69.40%	SRX3223680
Uterus	6,046,764	67.90%	SRX3223681
Pancreas	7,100,215	70.30%	SRX3223682
Lung	5,640,120	67.60%	SRX3223683
Skin	7,226,309	72.40%	SRX3223684
Eye	6,956,141	71.90%	SRX3223685
Shank	5,924,463	74.20%	SRX3223686

 Click here to access/download;Table;Table4.docx

Table 4. Comparison of genome completeness using BUSCO

Species	Assembly name	Complete		Fragment	Missing
		Single-copy	Duplication		
Chicken	Ogye_1.1	97.60%	0.50%	0.90%	1.00%
	Gallus_gallus-4.0	96.90%	0.90%	1.10%	1.10%
	Gallus_gallus-5.0	97.40%	0.90%	0.70%	1.00%
Turkey	Turkey_5.0	93.70%	0.50%	4.10%	1.70%
Duck	BGI_duck_1.0	92.60%	0.40%	4.80%	2.20%
Zebra finch	Taeniopygia_guttata-3.2.4	93.60%	2.20%	2.70%	1.50%

Click here to access/download;Table;Table5.docx

Table 5. Summary of methylated CpG sites across twenty tissues

	All genomic region			Promoter region		
	Total No. of sites	Methylated CpG sites		Total No. of sites	Methylated CpG sites	
		No. of sites	Fraction (%)		No. of sites	Fraction (%)
Breast	994,326	621,751	62.53	228,673	91,704	40.10
Liver	1,641,060	505,775	30.82	522,590	97,597	18.68
Bone marrow	1,096,466	671,781	61.27	254,978	100,385	39.37
Fascia	1,146,350	670,181	58.46	278,618	99,802	35.82
Cerebrum	1,246,514	748,323	60.03	298,677	112,689	37.73
Gizzard	1,024,125	609,010	59.47	234,379	85,273	36.38
Immature egg	1,416,686	809,214	57.12	334,813	115,195	34.41
Comb	1,035,966	642,138	61.98	239,319	92,436	38.62
Spleen	995,639	401,080	40.28	298,833	74,473	24.92
Mature egg	1,144,589	695,258	60.74	269,124	102,282	38.01
Cerebellum	1,279,666	775,513	60.60	305,489	117,950	38.61
Gall bladder	953,630	595,681	62.46	225,122	89,174	39.61
Kidney	1,016,035	610,941	60.13	238,066	89,255	37.49
Heart	1,000,957	611,343	61.08	235,853	90,434	38.34
Uterus	893,101	543,931	60.90	203,102	77,365	38.09
Pancreas	1,119,795	647,577	57.83	267,036	94,371	35.34
Lung	985,824	594,046	60.26	229,316	87,140	38.00
Skin	868,368	565,815	65.16	198,275	85,094	42.92
Eye	1,051,332	663,413	63.10	252,991	105,539	41.72
Shank	862,931	512,853	59.43	210,905	76,512	36.28

Figure 1 Click here to access/download;Figure;Figure1.pdf

A.

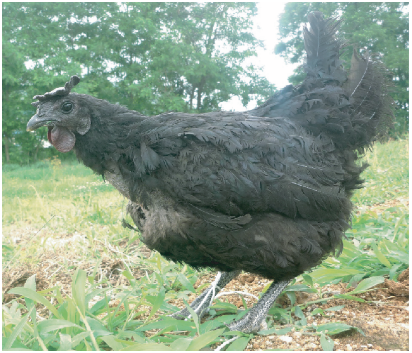

B.

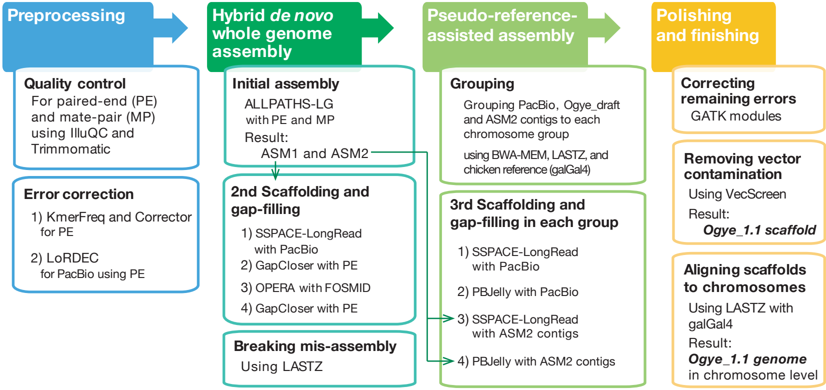

C.

Species	NCBI assembly name	Est. size (Gbp)	Assembly length (Gbp)	Pseudo-contig			Scaffold			Gaps in scaffold		Assembly method	
				Number	Ave. len. (Kbp)	NG50† (Kbp)	Number	Ave. len. (Kbp)	NG50† (Mbp)	Total length (Mbp)	Fraction (%)	Assembler	Sequencing platform
Chicken (Yeonsan Ogye)	Ogye_1.1	1.25 [10]	1.00	8,241	119.8	362.3	1,906	517.8	16.8	8.5	0.85	Our pipeline	I/P
Chicken (Red junglefowl) [8-10]	Gallus_gallus-4.0	1.25 [10]	1.05	27,142	38.1	211.9	16,846	62.1	11.0	13.4	1.28	Celara	S/4
	Gallus_gallus-5.0	1.25 [10]	1.22	24,701	49.3	2,718.7	23,870	51.2	6.3	2.8	0.23	MHAP/PBcR	I/S/4/P
Zebra finch [11]	Taeniopygia_guttata-3.2.4	1.22 [12]	1.23	124,806	9.8	38.8	37,422	32.9	8.5	8.7	0.71	PCAP	S
Turkey [13]	Turkey_5.0	1.28 [14]	1.13	296,315	3.7	26.7	233,806	4.8	3.0	35.0	3.11	MaSuRCA	IS/4
Hooded crow [15]	Hooded_Crow_genome	1.26 [15]	1.05	28,920	35.4	68.7	1,299	787.1	13.5	27.5	2.62	ALLPATHS-LG	I
Golden eagle [16]	Aquila_chrysaetos-1.0.2	1.28 [16]	1.19	17,032	69.3	156.4	1,142	1,033.3	8.7	12.7	1.07	ALLPATHS-LG	I
Medium ground-finch [8, 17]	GeoFor_1.0	1.25 [16]	1.07	95,828	10.9	24.0	27,239	38.2	3.7	24.0	2.25	ALLPATHS-LG	I
Blue-crowned manakin [18]	Lepidothrix_coronata-1.0	1.16 [12]	1.08	23,501	45.0	127.1	4,612	229.2	4.6	22.4	2.07	ALLPATHS-LG	I
White-throated sparrow [19]	Zonotrichia_albicollis-1.0.1	1.30 [20]	1.05	37,661	26.7	68.5	6,018	167.2	3.5	46.3	4.40	ALLPATHS-LG	I
Silvereye [21]	ASM128173v1	1.35 [12]	1.04	65,519	15.3	20.7	2,933	341.5	2.3	34.3	3.31	ALLPATHS-LG	I
Tibetan ground-tit [22]	PseHum1.0	1.22 [22]	1.04	27,052	38.1	132.7	5,406	190.5	11.8	13.0	1.24	SOAPdenovo	I
Bald eagle [8, 23, 24]	Haliaeetus_leucocephalus-4.0	1.40 [12]	1.18	31,786	36.5	82.3	1,023	1,133.2	7.4	19.2	1.63	SOAPdenovo	I
American crow [8, 25]	ASM69197v1	1.24 [20]	1.09	89,646	11.7	23.6	10,547	99.7	6.2	39.5	3.62	SOAPdenovo	I
Saker falcon [26]	F_cherrug_v1.0	1.19 [26]	1.17	75,898	15.2	30.2	5,863	196.3	4.1	23.8	2.03	SOAPdenovo	I
Peregrine falcon [8, 26]	F_peregrinus_v1.0	1.22 [26]	1.17	83,081	13.9	27.0	7,021	164.3	3.7	18.6	1.58	SOAPdenovo	I
Rock pigeon [27]	Cliv_1.0	1.30 [27]	1.11	100,099	10.9	21.3	14,923	72.8	2.5	21.1	1.90	SOAPdenovo	I
Budgerigar [28]	Melopsittacus_undulatus_6.3	1.19 [12, 29]	1.12	70,891	15.3	49.2	25,212	43.1	9.3	30.8	2.75	Celara	I/4
Little egret [8, 23, 30]	ASM68718v1	1.39 *	1.21	100,662	11.5	23.0	11,791	98.2	2.5	48.7	4.04	SOAPdenovo	I
Hoatzin [8, 31]	ASM69207v1	1.68 *	1.20	109,627	10.4	14.8	10,256	111.4	1.6	61.5	5.11	SOAPdenovo	I
Golden-collared manakin [8, 32]	ASM171598v1	1.38 *	1.21	29,998	38.9	137.7	15,315	76.3	13.1	45.5	3.75	MaSuRCA	I/P

* Estimated by k-mer counting method using KMC 2 [32] with 23-mer (SRR1144870-1 for Little egret, SRR947162-3 for Hoatzin, and SRR946955 for Golden-collared manakin.).

† In NG50 metric, estimated genome size is used rather than assembly length [34].

Figure 2 Click here to access/download;Figure;Figure2.pdf

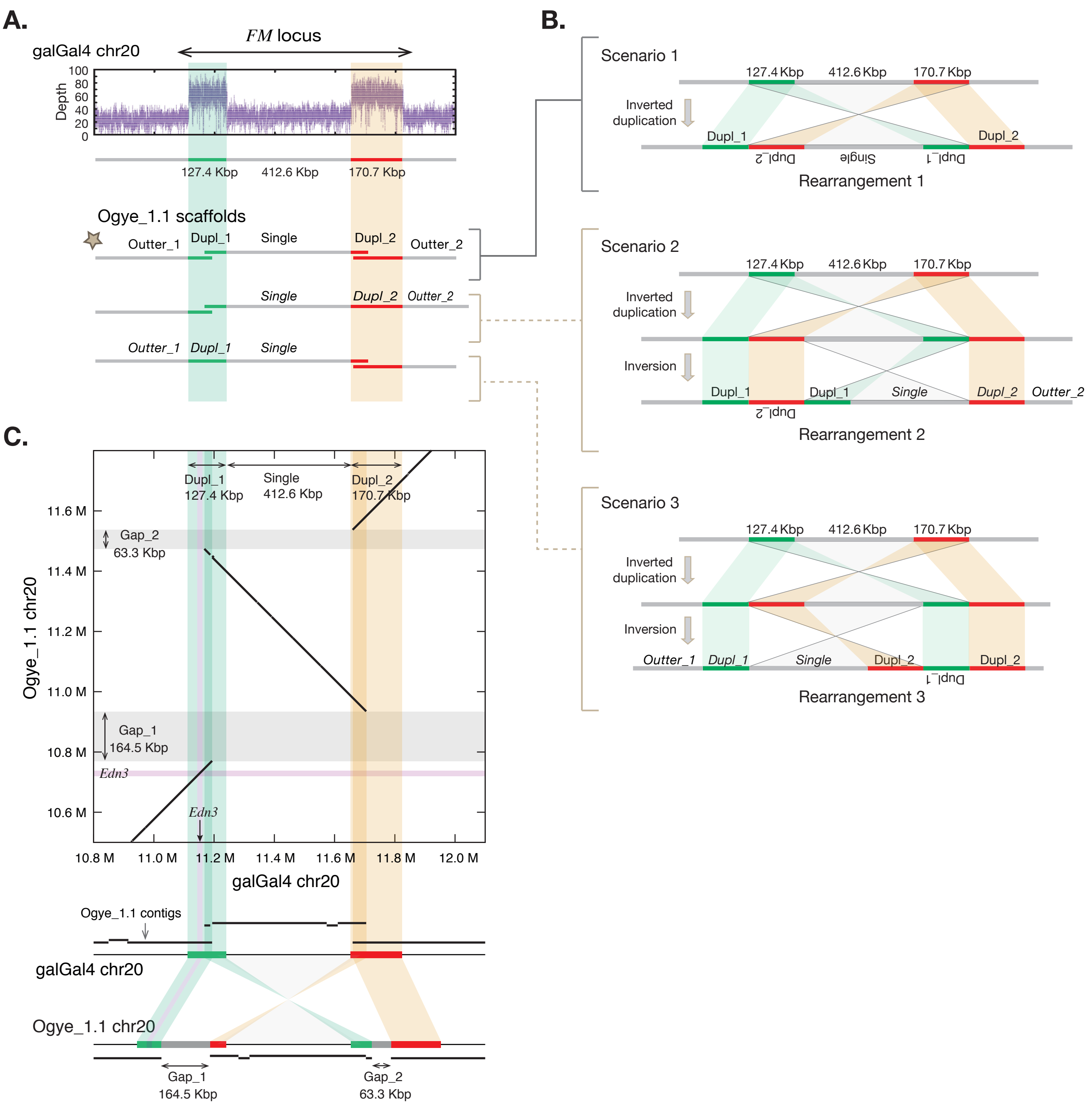

Figure 3

Click here to access/download;Figure;Figure3.pdf

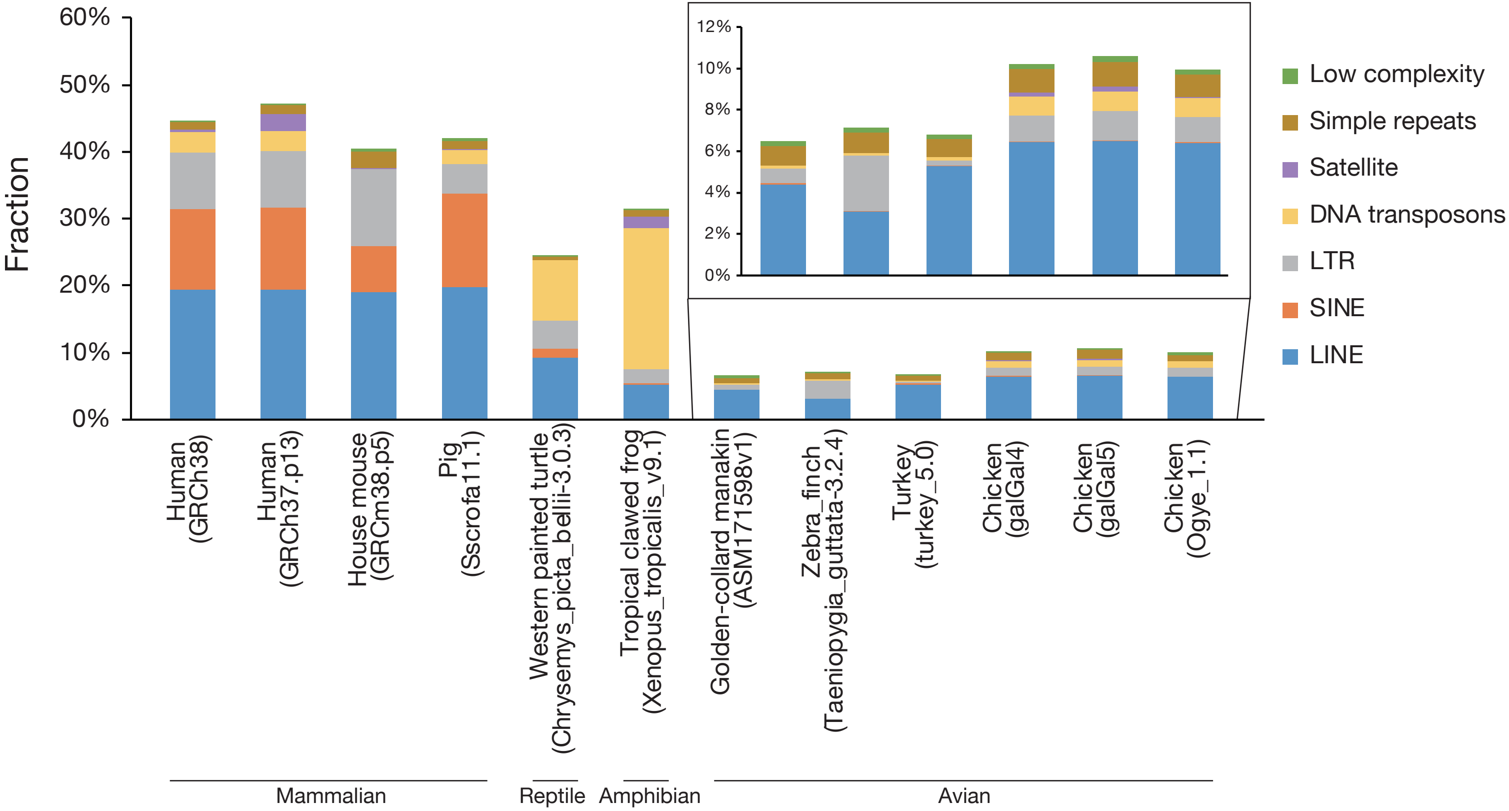

Figure 4

Click here to access/download;Figure;Figure4.pdf

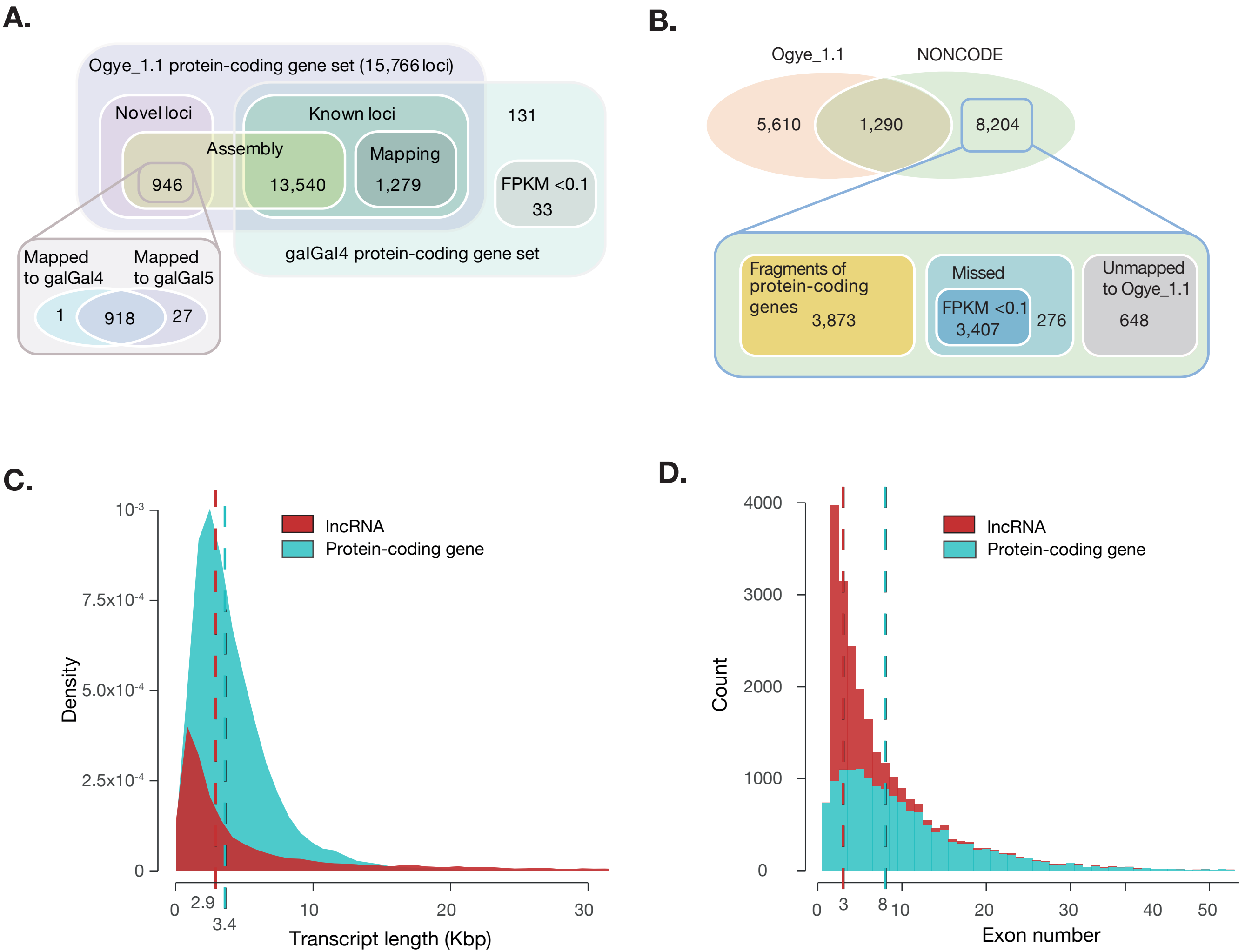

Figure 5

Click here to access/download;Figure;Figure5.pdf

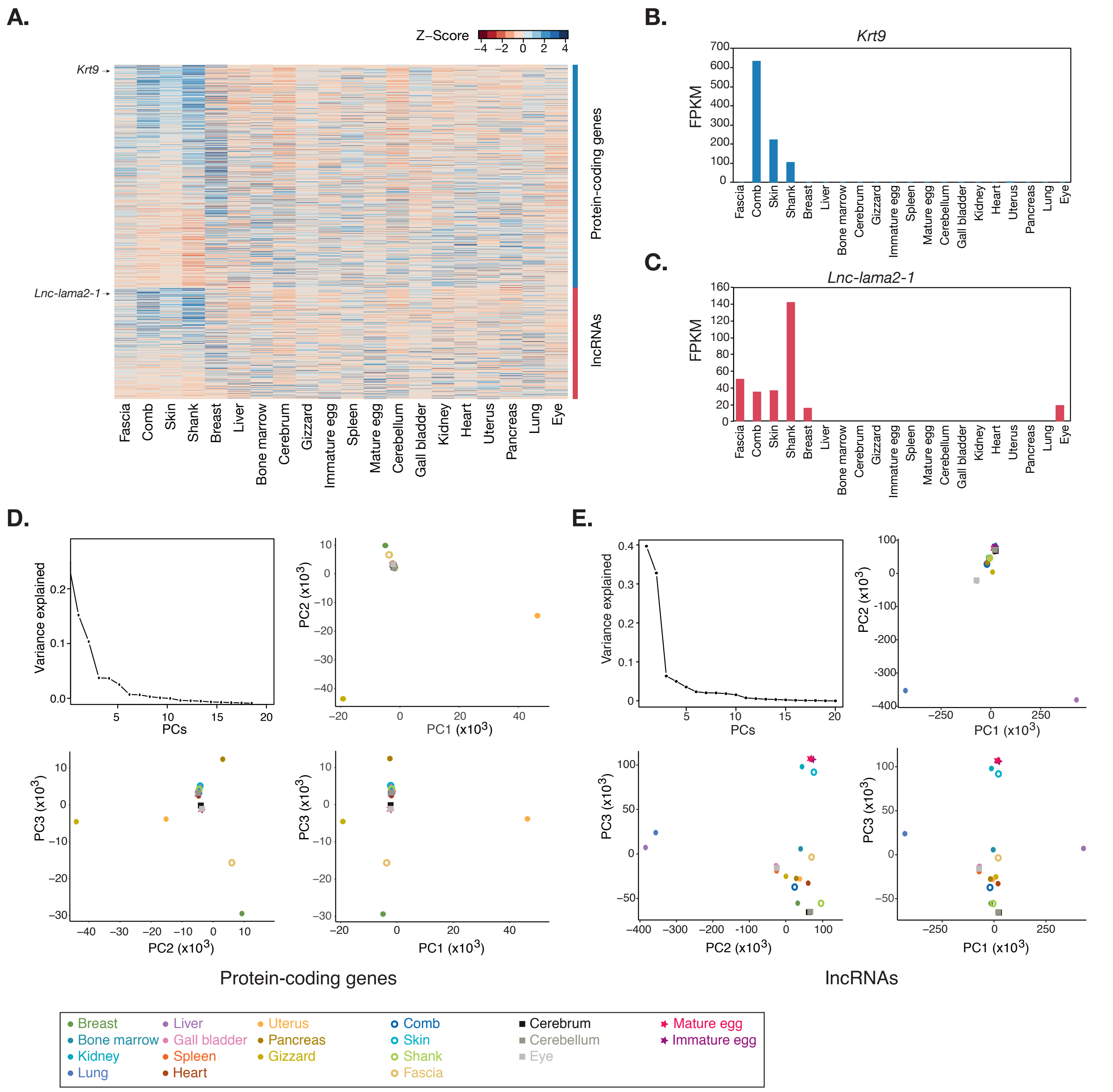

Figure 6

Click here to access/download;Figure;Figure6.pdf

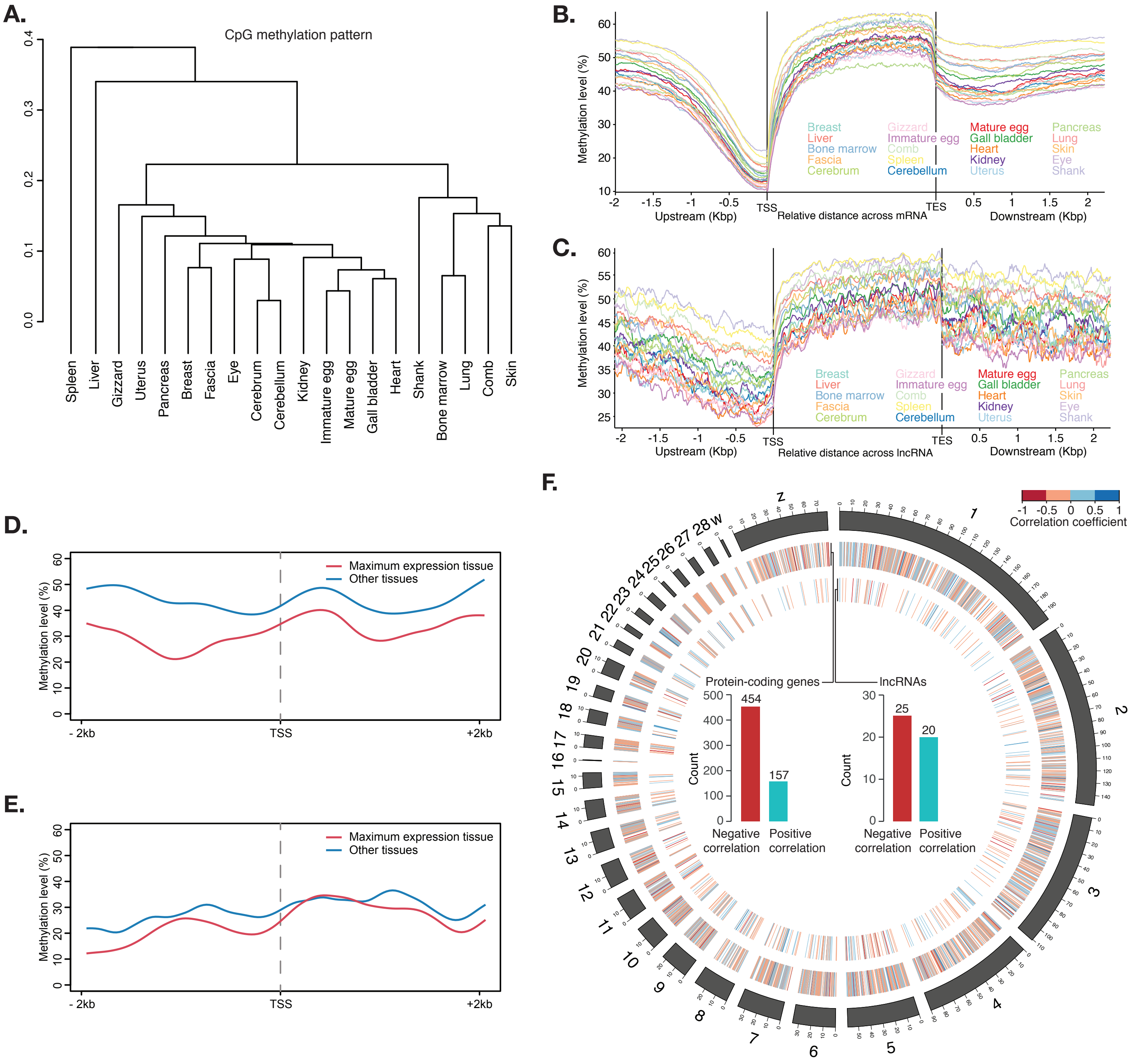

Supplementary Figures and Tables

Click here to access/download
Supplementary Material
Supplementary_Figure_Table.docx

Description (README) of available data in GigaDB

Click here to access/download
Supplementary Material
Supplementary_Data_README.docx

Command lines of programs and pipelines with run-time options used in this study

Click here to access/download
Supplementary Material
Supplementary_Command.docx
