# Supplementary material for: Whole genome and transcriptome maps of the entirely black native Korean chicken breed Yeonsan Ogye
Source: Gigascience. 2018 Jul 11;7(7):giy086. doi: 10.1093/gigascience/giy086 (PMC6065499; doi:10.1093/gigascience/giy086)
Supplement: Supplemental Files [file giy086_supplemental_files.zip › Supplementary_Command.docx]

**Commands of programs/pipelines with options**

**Whole genome assembly**

1. Preprocessing

a. Quality control

#step1

IlluQC_PRLL.pl -pe <left.fastq> <right.fastq> N A -s 20 \

-o <output.prefix>

#step2

trimmomatic-0.32.jar PE <left.fastq> <right.fastq> \

<left.P1.fastq> <left.U1.fastq> <right.P2.fastq> <right.U2.fastq> \

ILLUMINACLIP:/PATH/TO/TRIMMOMATIC/adapters/TruSeq3-PE-2.fa:2:30:10 \

LEADING:3 TRAILING:3 SLIDINGWINDOW:4:15 MINLEN:80

#step3

TrimmingReads.pl -i <left.fastq> -irev <right.fastq> -l 3 -r 5

b. Error correction

#making a list of paired-end data

ls /path-to-PE/ogye-280-?_P?.fq.gz > PE-280.list

ls /path-to-PE/ogye-500-?_P?.fq.gz > PE-500.list

#k-mer counting before short-read error-correction (default k-mer 17)

KmerFreq_AR -q 33 -p ogye-280 PE-280.list

KmerFreq_AR -q 33 -p ogye-500 PE-500.list

#short-read error-correction (default k-mer 17)

Corrector_AR -l 3 -Q 33 ogye-280.freq.cz ogye-280.freq.cz.len \

PE-280.lst

Corrector_AR -l 3 -Q 33 ogye-280.freq.cz ogye-500.freq.cz.len \

PE-500.lst

#LoRDEC: hybrid-long-read error-correction

lordec-correct -T 30 -S lordec_CR_OG_pacbio.stat \

-i /path-to-pacbio/OG_60cells_filtered_subreads.fastq \

-2 /path-to-PE-280/EC-PE-280.lst \

-k 23 -o /path-to-result/lordec_CR_OG_pacbio.fastq \

-s 3

#trimming LoRDEC result

lordec-trim -i lordec_CR_OG_pacbio.fasta -o LoRDEC_K23.fasta

#list of EC-PE-280.txt (error-corrected paired-end 280 library)

$cat EC-PE-280.txt

/path-to-EC-PE/EC-PE-280-1_1.fq.gz

/path-to-EC-PE/EC-PE-280-1_2.fq.gz

/path-to-EC-PE/EC-PE-280-2_1.fq.gz

/path-to-EC-PE/EC-PE-280-2_2.fq.gz

1. De novo assembly
   1. ALLPATHS-LG

#in_libs.csv and in_groups.csv are required for ALLPATHS-LG

$ cat in_libs.csv

library_name, project_name, organism_name, type, paired, frag_size, frag_stddev, insert_size, insert_stddev, read_orientation, genomic_start, genomic_end

280, ogye, ogye, fragment, 1, 280, 20, , , inward, 0, 0

500, ogye, ogye, fragment, 1, 500, 50, , , inward, 0, 0

3kb, ogye, ogye, jumping, 1, , , 3000, 600, outward, 0, 0

5kb, ogye, ogye, jumping, 1, , , 5000, 1000, outward, 0, 0

$ cat in_groups.csv

file_name, library_name, group_name

/path-to-EC-PE/ogye-280-1_P?.fq.gz, 280, 280-1

/path-to-EC-PE/ogye-500-1_P?.fq.gz, 500, 500-1

/path-to-EC-PE/ogye-500-2_P?.fq.gz, 500, 500-2

/path-to-EC-PE/ogye-500-3_P?.fq.gz, 500, 500-3

/path-to-EC-PE/ogye-500-6_P?.fq.gz, 500, 500-6

/path-to-MP-data/ogye-MP-3kb-2_P?.fq.gz, 3kb, 3kb-2

/path-to-MP-data/ogye-MP-5kb-1_P?.fq.gz, 5kb, 5kb-1

/path-to-MP-data/ogye-MP-8kb-1_P?.fq.gz, 8kb, 8kb-1

/path-to-MP-data/ogye-MP-10kb-2_P?.fq.gz, 10kb, 10kb-2

#prepare ALLPATHS-LG

PrepareAllPathsInputs.pl \

DATA_DIR=/path-to-assembly/pre/OGYE/ASSEMBLY \

PICARD_TOOLS_DIR=/path-to/picard-tools-1.117 \

PHRED_64=0 PLOIDY=2 HOSTS=20 TMP_DIR=/path-to-assembly/.tmp \

OVERWRITE=True

#run ALLPATHS-LG

RunAllPathsLG \

MAXPAR=2 \

THREADS=40 \

PRE=/path-to-assembly/pre \

REFERENCE_NAME=OGYE \

DATA_SUBDIR=ASSEMBLY \

RUN=RUN \

TARGETS=standard \

EVALUATION=NONE \

K=96 \

OVERWRITE=True \

MAX_MEMORY_GB=500 \

HAPLOIDIFY=True

$ ln -s /path-to-result/final.assembly.fasta ASM1.fasta

- 1. SSPACE-LongRead

SSPACE-LongRead.pl -c ASM1.fasta -p LoRDEC_K23.fasta -i 97 -l 7 \

-b SLR-i97-l7

- 1. GapCloser

GapCloser -a /path-to/SLR-i97-l7/scaffold.fasta -b GapCloser.cfg \

-o ASM1.SLR-i97.GapCloser.fasta -l 151 -t 50

$ cat GapCloser.cfg

max_rd_len=151

[LIB]

avg_ins=280

reverse_seq=0

asm_flags=4

rank=1

q1=/path-to-EC-PE/EC-PE-280-2_1.fq.gz

q2=/path-to-EC-PE/EC-PE-280-2_2.fq.gz

[LIB]

avg_ins=500

reverse_seq=0

asm_flags=4

rank=1

q1=/path-to-EC-PE/EC-PE-500-2_1.fq.gz

q2=/path-to-EC-PE/EC-PE-500-2_2.fq.gz

q1=/path-to-EC-PE/EC-PE-500-4_1.fq.gz

q2=/path-to-EC-PE/EC-PE-500-4_2.fq.gz

q1=/path-to-EC-PE/EC-PE-500-6_1.fq.gz

q2=/path-to-EC-PE/EC-PE-500-6_2.fq.gz

- 1. Opera

$ cat opera.sh

bwa index ASM1.SLR-i97.GapCloser.fasta.fasta

bwa mem -t 30 ASM1.SLR-i97.GapCloser.fasta \

FOSMID_1.fasta FOSMID_2.fasta > FOSMID.sam

opera opera.cfg

$ cat opera.cfg

output_folder=./

contig_file=ASM1.SLR-i97.GapCloser.fasta

kmer=23

[LIB]

map_file=FOSMID.sam

lib_mean=40000

lib_std=4000

read_ori=in

cluster_increased_step=1

$ ./opera.sh

$ ln -s scaffoldSeq.fasta ASM1.SLR-i97.GapCloser.Opera.fasta

- 1. GapCloser

GapCloser -a /path-to/Opera/ ASM1.SLR-i97.GapCloser.Opera.fasta \

-b GapCloser.cfg \

-o ASM1.SLR-i97.GapCloser.Opera.GapCloser.fasta -l 151 -t 50

$ cat GapCloser.cfg

max_rd_len=151

[LIB]

avg_ins=280

reverse_seq=0

asm_flags=4

rank=1

q1=/path-to-EC-PE/EC-PE-280-2_1.fq.gz

q2=/path-to-EC-PE/EC-PE-280-2_2.fq.gz

[LIB]

avg_ins=500

reverse_seq=0

asm_flags=4

rank=1

q1=/path-to-EC-PE/EC-PE-500-2_1.fq.gz

q2=/path-to-EC-PE/EC-PE-500-2_2.fq.gz

q1=/path-to-EC-PE/EC-PE-500-4_1.fq.gz

q2=/path-to-EC-PE/EC-PE-500-4_2.fq.gz

q1=/path-to-EC-PE/EC-PE-500-6_1.fq.gz

q2=/path-to-EC-PE/EC-PE-500-6_2.fq.gz

- 1. Breaking

lastz galGal4.fa \

ASM1.SLR-i97.GapCloser.Opera.GapCloser.fasta[multiple] \

--ambiguous=iupac --step=10000 --format=general \

> ASM1.SLR-i97.GapCloser.Opera.GapCloser.lastz

Structural variations were detected using our tool.

(<https://github.com/sohnjangil/lastz_analysis.git>)

The structural variations with size >1Mbp have been assumed as mis-assemblies (see Figure S2), and they were validated using igv.

30 mis-assemblies were detected, resulting

Ogye_draft.fasta

1. Pseudo-reference-assisted assembly
   1. BWA for PacBio to scaffolds

bwa index Ogye_draft.fasta

bwa mem -x pacbio Ogye_draft.fasta \

LoRDEC_K23.fasta > LoRDEC_K23.sam

- 1. LASTZ for scaffold to galGal4

lastz galGal4.fa Ogye_draft.fasta[multiple] \

--ambiguous=iupac --step=10000 --format=general \

> Ogye_draft.lastz

lastz galGal4.fa ASM2.fasta[multiple] \

--ambiguous=iupac --step=10000 --format=general \

> ASM2.lastz

- 1. Grouping scaffolds and error-corrected long-read to each chromosome group using own program

(<https://github.com/sohnjangil/tsrator.git>)

- 1. SSPACE-LongRead for each chromosome group using error-corrected long-reads

In case of chr1 group,

$ SSPACE-LongRead.pl -c scaffold_chr1.fasta \

-p LoRDEC_K23_chr1.fasta -t 40 -i97 -l 1 -s 1

$ ln -s scaffolds.fasta scaffold_chr1.SLR.fasta

- 1. PBJelly for each chromosome group using error-corrected long-reads

In case of chr1 group,

$ cat PBJelly_chr1.sh

Jelly.py setup PBJelly_chr1.xml

Jelly.py mapping PBJelly_chr1.xml

Jelly.py support PBJelly_chr1.xml

Jelly.py extraction PBJelly_chr1.xml

Jelly.py assembly PBJelly_chr1.xml -x "--nproc=20"

Jelly.py output PBJelly_chr1.xml

$ cat PBJelly_chr1.xml

<jellyProtocol>

<reference>scaffold_chr1.SLR.fasta</reference>

<outputDir>PBJelly_chr1</outputDir>

<blasr>-minMatch 8 -minPctIdentity 70 -bestn 1 -nCandidates 20 -maxScore -500 -nproc 20 -noSplitSubreads</blasr>

<input baseDir="./">

<job>LoRDEC_K23_chr1.fasta</job>

</input>

</jellyProtocol>

$ PBJelly_chr1.sh

$ ln -s jelly.out.fasta scaffold_chr1.SLR.PBJelly.fasta

- 1. SSPACE-LongRead for each chromosome group using ASM2 contigs

In case of chr1 group,

$ SSPACE-LongRead.pl -c scaffold_chr1.SLR.PBJelly.fasta \

-p LoRDEC_K23_chr1.fasta -t 40 -i97 -l 1 -s 1

$ ln -s scaffolds.fasta scaffold_chr1.SLR.PBJelly.SLR.fasta

- 1. PBJelly with another ALLPATHS-LG scaffold for each chromosome group using ASM2 contigs

In case of chr1 group,

$ cat PBJelly.sh

Jelly.py setup PBJelly_chr1.ASM2.xml

Jelly.py mapping PBJelly_chr1.ASM2.xml

Jelly.py support PBJelly_chr1.ASM2.xml

Jelly.py extraction PBJelly_chr1.ASM2.xml

Jelly.py assembly PBJelly_chr1.ASM2.xml -x "--nproc=20"

Jelly.py output PBJelly_chr1.ASM2.xml

$ cat PBJelly_chr1.ASM2.xml

<jellyProtocol>

<reference>scaffold_chr1.SLR.PBJelly.SLR.fasta</reference>

<outputDir>PBJelly</outputDir>

<blasr>-minMatch 8 -minPctIdentity 70 -bestn 1 -nCandidates 20 -maxScore -500 -nproc 20 -noSplitSubreads</blasr>

<input baseDir="./">

<job>ASM2_chr1.fasta</job>

</input>

</jellyProtocol>

$ PBJelly.sh

$ ln -s jelly.out.fasta scaffold_chr1.SLR.PBJelly.SLR.PBJelly.fasta

- 1. Collecting

$ cat (all results) > draft_genome.fa

1. Polishing
   1. VecScreen

This step has been done by our own pipeline IterativeVecscreenPipeline

(<https://github.com/sohnjangil/IVP.git>)

IVP <draft_genome.fa(sta)> <Vector_file> <working_dir>

Or it can be done manually using vecscreen, which is included in NCBI C++ Toolkit

(https://www.ncbi.nlm.nih.gov/IEB/ToolBox/CPP_DOC/).

- 1. GATK

GenomeAnalysisTK.jar -T UnifiedGenotyper -R <genome.fasta> \

-I <mapped bamfile> -o <output.vcf>

GenomeAnalysisTK.jar -T VariantFiltration -R <genome.fasta> \

-V <vcffile> --filterExpression "MQ0 > 4.0 || QD < 5.0 || \

FS > 200 " --filterName "my_indel_filter" -o <filtered.vcf>

- 1. LASTZ to galGal4 for connecting scaffolds

In case of chr1 group,

lastz galGal4_chr1.fa scaffold_chr1.SLR.PBJelly.fasta[multiple] \

--ambiguous=iupac --step=1000 --format=general \

> scaffold_chr1.SLR.PBJelly.lastz

- 1. Connecting scaffolds to each chromosome introducing 100 Kbp gaps using own program

(<https://github.com/sohnjangil/lastz_analysis.git>)

- 1. In case of FM locus (in chr20), we have rearranged the scaffolds (please see main manuscript)

Ogye_1.1.genome.fasta.

**Repeat annotations**

1. RepeatMasker

RepeatMasker -species chicken -q Ogye_1.1.genome.fasta

**SNPs/INDELs**

1. bwa mem

#samtools version 1.7

bwa mem -t 60 -M Ogye_1.1.genome.fasta left.fastq right.fastq \

| samtools view -Sbh - > aligned.bam

samtools sort aligned.bam > sorted.bam

1. MarkDuplicates

picard.jar MarkDuplicates I=sorted.bam O=markdu.bam M=markdu.txt \

REMOVE_DUPLICATES=true

1. Mpileup

#samtools version 1.7

samtools mpileup -f Ogye_1.1.genome.fasta -o output.mpileup markdu.bam

1. mpileup2snp

java -jar /path-to-vascan/VarScan.v2.3.9.jar mpileup2snp \

--min-coverage 8 --min-reads2 2 --min-avg-qual 15 --min-var-freq 0.2 \

--p-value 1e-2 --output-vcf output.mpileup > Ogye_SNP.vcf

1. mpileup2indel

java -jar /path-to-varscan/VarScan.v2.3.9.jar mpileup2indel \

--min-coverage 8 --min-reads2 2 --min-avg-qual 15 --min-var-freq 0.2 \

--p-value 1e-2 --output-vcf output.mpileup > Ogye_indel.vcf

**RNA-seq preprocessing**

1. Sickle

#iterate for all paired-end RNA-seq

for i in {1..20}

do

sickle pe -t sanger -q 20 -l 20 \

-f raw_RNAs_pe_${i}_1.fastq -r raw_RNAs_pe_${i}_2.fastq \

-o RNAs_pe_${i}_1.fastq -p RNAs_pe_${i}_2.fastq \

-s RNAs_pe_${i}_single.fastq

done

#iterate for all single-end RNA-seq

for i in {1..20}

do

sickle se -t sanger -q 20 -l 20 \

-f raw_RNAs_se_${i}.fastq -o RNAs_se_${i}.fastq

done

**Protein-coding gene annotation**

1. STAR

for i in {1..20} ; do

STAR --runThreadN 20 --runMode alignReads --outReadsUnmapped Fastx \

--outFilterType BySJout --outFilterMismatchNmax 999 \

--outFilterMultimapNmax 20 --alignSJoverhangMin 8 \

--alignSJDBoverhangMin 1 --alignIntronMin 20 --alignIntronMax 1000000 \

--alignMatesGapMax 1000000 --outFilterMismatchNoverLmax 0.02 \

--outSAMtype BAM SortedByCoordinate --genomeDir ./ --outWigType wiggle \

--outWigStrand Stranded --outWigNorm RPM \

--outFileNamePrefix RESULT/PE-${i} \

--readFilesIn RNAs_pe_${i}_1.fastq RNAs_pe_${i}_2.fastq

done

1. StringTie

#assembly

for i in {1..20} ; do

stringtie PE-${i}Aligned.sortedByCoord.out.bam --rf -o \

stringtie.c5.${i}.gtf -p 20 -c 5 &

done

#merge

ls stringtie.c5.*.gtf > gtf_list.txt

stringtie --merge -F 0.1 -T 0.1 -o Merged_F0.1_T0.1.gtf gtf_list.txt

1. TransDecoder

##This script makes Merged_F0.1_T0.1.TransDecoder.gtf

input= Merged_F0.1_T0.1

ln -s Ogye_1.1.genome.fasta genome.fa

#step1

cufflinks_gtf_genome_to_cdna_fasta.pl input.gtf genome.fa \

> input.fasta

#step2

cufflinks_gtf_to_alignment_gff3.pl input.gtf > input.gff3

#step3

TransDecoder.LongOrfs --gene_trans_map input.gene_to_trans.map \

-m 10 -t input.fasta

#step4

TransDecoder.Predict --all_good_orfs -t input.fasta

#step5

cdna_alignment_orf_to_genome_orf.pl input.fasta.transdecoder.gff3 \

input.gff3 input.fasta > input.TransDecoder.gff3

gffread input.TransDecoder.gff3 -T input.TransDecoder.gtf

##extracting intact-structured

grep "ORF type:complete" Merged_F0.1_T0.1.fasta.transdecoder.cds \

> complete.list

fasta_extract Merged_F0.1_T0.1.fasta.transdecoder.cds complete.list

for i in {$(cat complete.list)}

do

samtools faidx Merged_F0.1_T0.1.fasta.transdecoder.cds $i \

> Merged_F0.1_T0.1.fasta.transdecoder.cds.complete.fa

done

1. CPAT

#preparation of CPAT

ln -s galGal4_protein_coding_gene.CDS.fa CODING.fasta

ln -s NONCODE2016_chicken.fa NONCODING.fasta

make_hexamer_tab.py -c CODING.fasta -n NONCODING.fasta > chicken.hex.tsv

make_logitModel.py -c CODING.fasta -n NONCODING.fasta \

-x chicken.hex.tsv -o chicken

#running CPAT

cpat.py -g Merged_F0.1_T0.1.fasta.transdecoder.cds.complete.fa \

-o CPAT.result \

-x chicken.hex.tsv -d chicken.logit.RData

##The output format of .CPAT is as follows

mRNA_size ORF_size Fickett_score Hexamer_score coding_prob

MSTRG.1000::MSTRG.1000.1::g.30240::m.30240 690 690 1.2314 0.249684770852 0.979605792287188

MSTRG.1000::MSTRG.1000.2::g.30259::m.30259 861 861 1.3152 0.331988614497 0.994180677859508

MSTRG.1000::MSTRG.1000.3::g.30269::m.30269 1506 1506 1.2058 0.464416676136 0.999806068615617

MSTRG.1000::MSTRG.1000.4::g.30224::m.30224 1977 1977 1.2058 0.451328343349 0.999974953688727

#Thus, extract the list of transcript as follows

awk '{if($6>0.99) print $1}' CPAT.result > CPAT.0.99.txt

awk '{if($6<=0.99 && $6>0.8) print $1}' CPAT.result > CPAT.0.8-0.99.txt

1. CPC

/path-to-CPC/bin/run_predict.sh \

Merged_F0.1_T0.1.TransDecoder.complete.CPAT_0.8-0.99.fasta \

Merged_F0.1_T0.1.TransDecoder.complete.CPAT_0.8-0.99.fasta.predict \

Workdir \

Merged_F0.1_T0.1.TransDecoder.complete.CPAT_0.8-0.99.fasta.evidence

1. GMAP

gmap_build -D /path-to/gmap_index -d Ogye_1.1 Ogye_1.1.genome.fasta

gmap -D /path-to/gmap_index -d Ogye_1.1 \

--min-intronlength=20 --intronlength=1000000 -n 3 -t 60 -f gff3_gene \

Gallus_gallus.Galgal4.cdna.all.fa \

> GG4_to_OG.cdna.n3.gff3 2> GG4_to_OG.cdna.n3.gff3.err

### NOTE: The list of unpresented protein-coding genes in Ogye_1.1 genome is listed in GG4_to_OG.cdna.n3.gff3.err

1. RSEM

#preparation

prep="rsem-prepare-reference"

GTF="Ogye_protein-coding.gtf"

REF="Ogye_1.1.genome.fasta"

rsem_ref="$PWD/Ogye_1.1"

opt="--gtf $GTF --star -p 60"

cmd="${prep} ${opt} ${REF} ${rsem_ref}"

echo $cmd

eval $cmd

#run RSEM

expr="rsem-calculate-expression"

opt="--star --paired-end --star-gzipped-read-file -p 60"

for i in {1..20}

do

OUT="RESULT/Ogye_RNAs_pe_${i}"

DATA="RNAs_pe_${i}_1.fastq.gz RNAs_pe_${i}_2.fastq.gz"

cmd="${expr} ${opt} ${DATA} ${rsem_ref} ${OUT}"

echo $cmd > RSEM.${i}.log 2> RSEM.${i}.err

eval $cmd >> RSEM.${i}.log 2>> RSEM.${i}.err &

done

**lncRNA annotation**

1. STAR

for i in {1..20} ; do

STAR --runThreadN 20 --runMode alignReads --outReadsUnmapped Fastx \

--outFilterType BySJout --outFilterMismatchNmax 999 \

--outFilterMultimapNmax 20 --alignSJoverhangMin 8 \

--alignSJDBoverhangMin 1 --alignIntronMin 20 --alignIntronMax 1000000 \

--alignMatesGapMax 1000000 --outFilterMismatchNoverLmax 0.02 \

--outSAMtype BAM SortedByCoordinate --genomeDir ./ --outWigType wiggle \

--outWigStrand Stranded --outWigNorm RPM \

--outFileNamePrefix RESULT/PE-${i} \

--readFilesIn RNAs_pe_${i}_1.fastq RNAs_pe_${i}_2.fastq

done

1. cufflinks

for i in {1..20}

do

cufflinks --library-type fr-firststrand \

PE-${i}Aligned.sortedByCoord.out.bam

done

1. cuffmerge

ls *.gtf > assembly_GTF_list.txt

cuffmerge assembly_GTF_list.txt

1. CPC

/path-to-CPC/bin/run_predict.sh \

assembled_transcript.fasta \

assembled_transcript.fasta.predict \

Workdir \

assembled_transcript.fasta.evidence

**RRBS**

1. bismark

bismark --bowtie2 -p 4 --bam \

--samtools_path /path-to/samtools-0.1.19/ \

-o outDir -temp_dir tempDir genome_folder infile

1. bismark_methylation_extractor

bismark_methylation_extractor -s --comprehensive --report --bedGraph \

--cytosine_report --genome_folder genomeDir -o outDir infile
