# Supplementary material for: Whole genome and transcriptome maps of the entirely black native Korean chicken breed Yeonsan Ogye
Source: Gigascience. 2018 Jul 11;7(7):giy086. doi: 10.1093/gigascience/giy086 (PMC6065499; doi:10.1093/gigascience/giy086)
Supplement: Supplemental Files [file giy086_supplemental_files.zip › Supplementary_Data_README.docx]

**Whole genome and transcriptome maps of the entirely black native Korean chicken breed Yeonsan Ogye**

=====================================================================================================

Directory lists:

-------------------------------

1 - lncRNA

2 - Protein_coding_gene

3 - RRBS

4 - SNPs_INDELs

5 - Repeat

6 - Genome

Files:

----------------

1. lncRNA/

# ogye1.1_lncRNA_annotation.gtf

lncRNA annotations comprising 6,900 novel and kwon lncRNAs identified from 20 tissues of Ogye

# ogye1.1_lncRNA_expression_table.txt

Tab-delimited expression table of lncRNAs. Fragments Per Kilobase of exons per million mapped reads (FPKM) was calculated using the RSEM program. The values across tissues were normalized using the quantile normalization method.

2. Protein_coding_gene/

# ogye1.1_PCG_annotation.gtf

Protein-coding gene annotations comprising 15,766 genes identified from 20 tissues.

# ogye1.1_PCG_CDS.fasta

CDS sequences of ogye1.1 protein-coding genes.

# ogye1.1_PCG_GENE.fasta

Gene sequences of ogye1.1 protein-coding genes.

# ogye1.1_PCG_SE_expression_table.xlsx

The tab-delimited expression table of protein-coding genes using single-end RNA seq libraries. The expression (FPKM) was calculated using RSEM programThe values across tissues were normalized using the quantile normalization method.

# ogye1.1_PCG_PE_expression_table.xlsx

The tab-delimited expression table of protein-coding genes using paired-end RNA seq libraries. The expression (FPKM) was calculated using RSEM programThe values across tissues were normalized using the quantile normalization method.

3. RRBS/

# ogye1.1_RRBS_[xxxxxx].bedgraph, where [xxxxxx] is a tissue name.

Tab-delimited CpG methylation signal across 20 tissues. The signals were calculated using the Bismark CpG coverage report, which Include 1-based genomic coordinates for every covered cytosine position in each sample in the following format: <chromosome> <start position> <end position> <methylation percentage> <count methylated> <count non-methylated>.

4. SNPs_INDEL/

# ogye1.1.total.snps.filtered.vcf

SNP and insertion and deletion (INDEL) were called using VarScan 2 with options -mfileup2snp --min-coverage 8 --min-reads2 2 --min-avg-qual 15 --min-var-freq 0.2 --p-value 1e-2.

# ogye1.1.total.indels.filtered.vcf

SNP and insertion and deletion (INDEL) were called using VarScan 2 with options -mfileup2indel --min-coverage 8 --min-reads2 2 --min-avg-qual 15 --min-var-freq 0.2 --p-value 1e-2.

5. Repeat/

# ogye1.1.fasta.out

Repeat lists identified from an input genome sequence using the RepeatMasker ver.4-0-6.

# ogye1.1.fasta.tbl

The summary table of repeats identified from an input genome sequence using the RepeatMasker ver.4-0-6.

6. Genome/

# ogye1.1.fasta

A genome sequence of Yeonsan Ogye genome assembly version 1.1., which include chromosome sequences and unplaced sequences.
