# Supplementary material for: Whole genome and transcriptome maps of the entirely black native Korean chicken breed Yeonsan Ogye
Source: Gigascience. 2018 Jul 11;7(7):giy086. doi: 10.1093/gigascience/giy086 (PMC6065499; doi:10.1093/gigascience/giy086)
Supplement: Supplemental Files [file giy086_supplemental_files.zip › Supplementary_Figure_Table.docx]

**Supplementary figures**

**Figure S1.** Ogye_1.1 genome assembly statistics at each step.


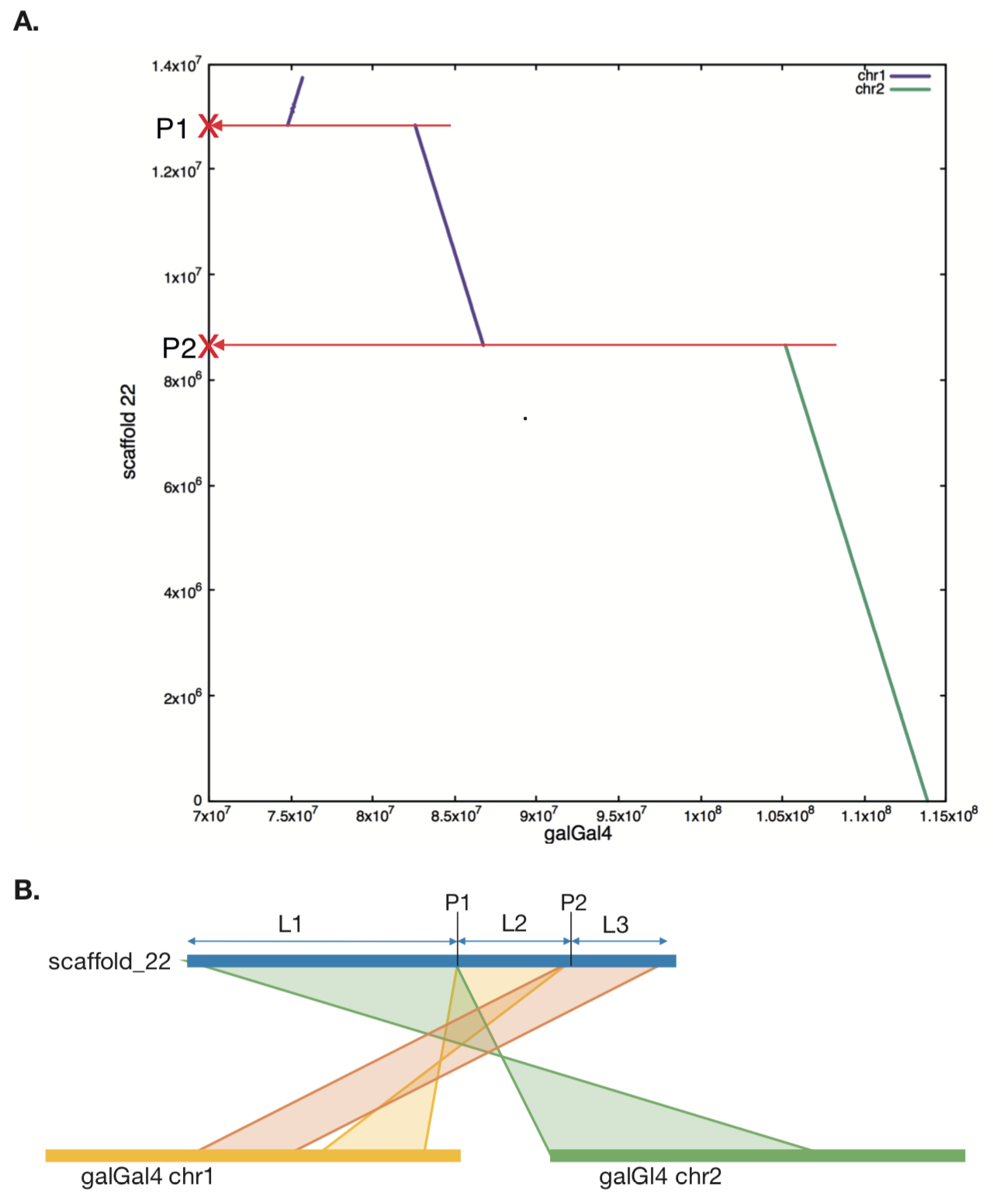


**Figure S2. A.** An example of mis-assemblies in a scaffold. The x-axis represents the positions on chr1 or chr2 in galGal4 and the y-axis represents the position in scaffold_22 of the scaffold at the second step of the second stage (i.e. Opera scaffolder’s result); **B.** In this example, there are two translocations: at P1 between L1 and L_2 and at P2 between L2_and L3. Since L_1, L_2 and L_3 are all >1Mbp, we broke the scaffold at P1 and P2. In this manner, we found 30 break points over all scaffolds in the breaking step of the second stage in **Figures 1B and S1**.


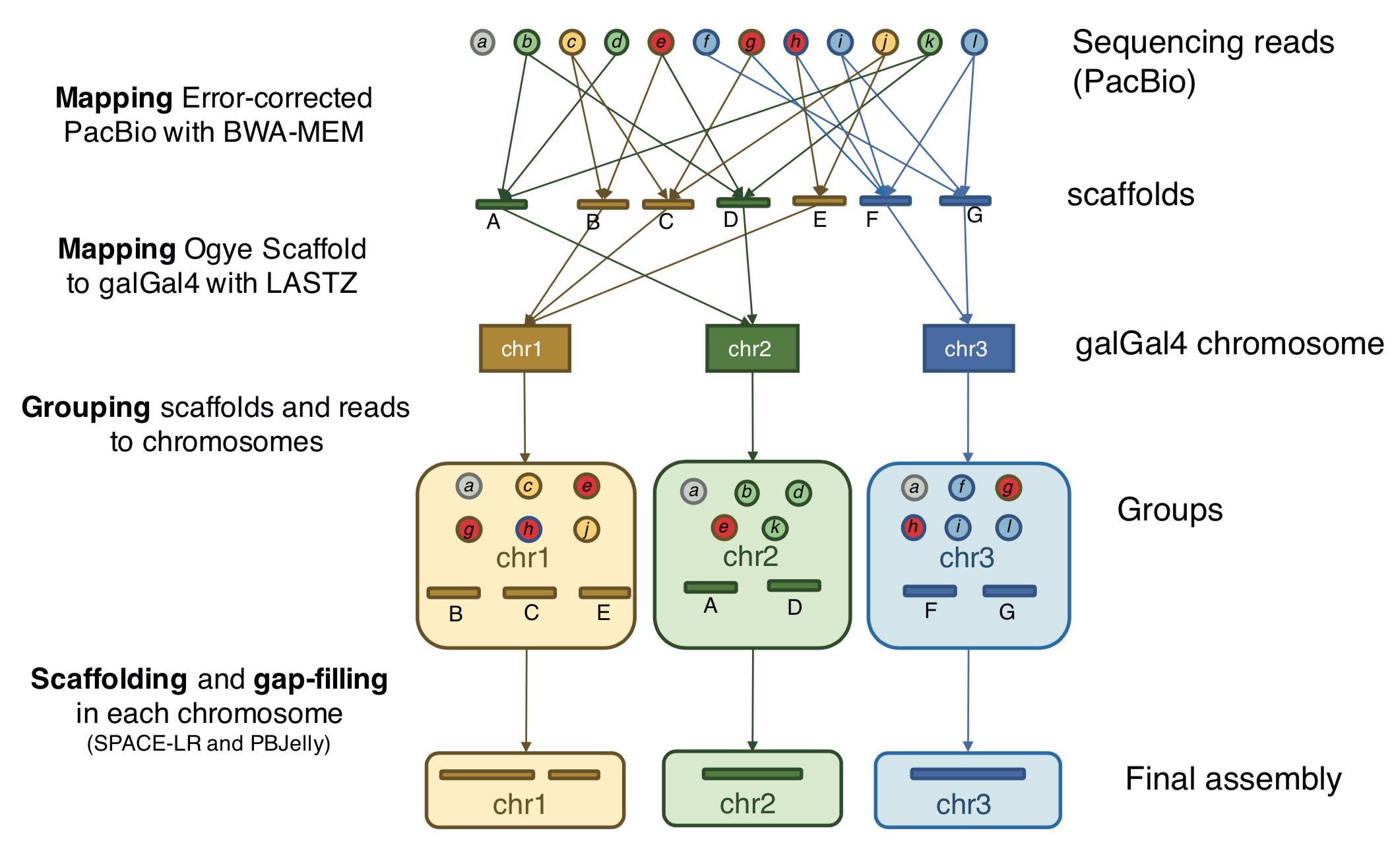


**Figure S3.** Pseudo-reference-assisted assembly pipeline utilizing a hierarchical bipartite graph of PacBio long reads, scaffolds, and galGal4 chromosomes. The tools, used in grouping PacBio reads and scaffolds, are available in <https://github.com/sohnjangil/tsrator.git>.


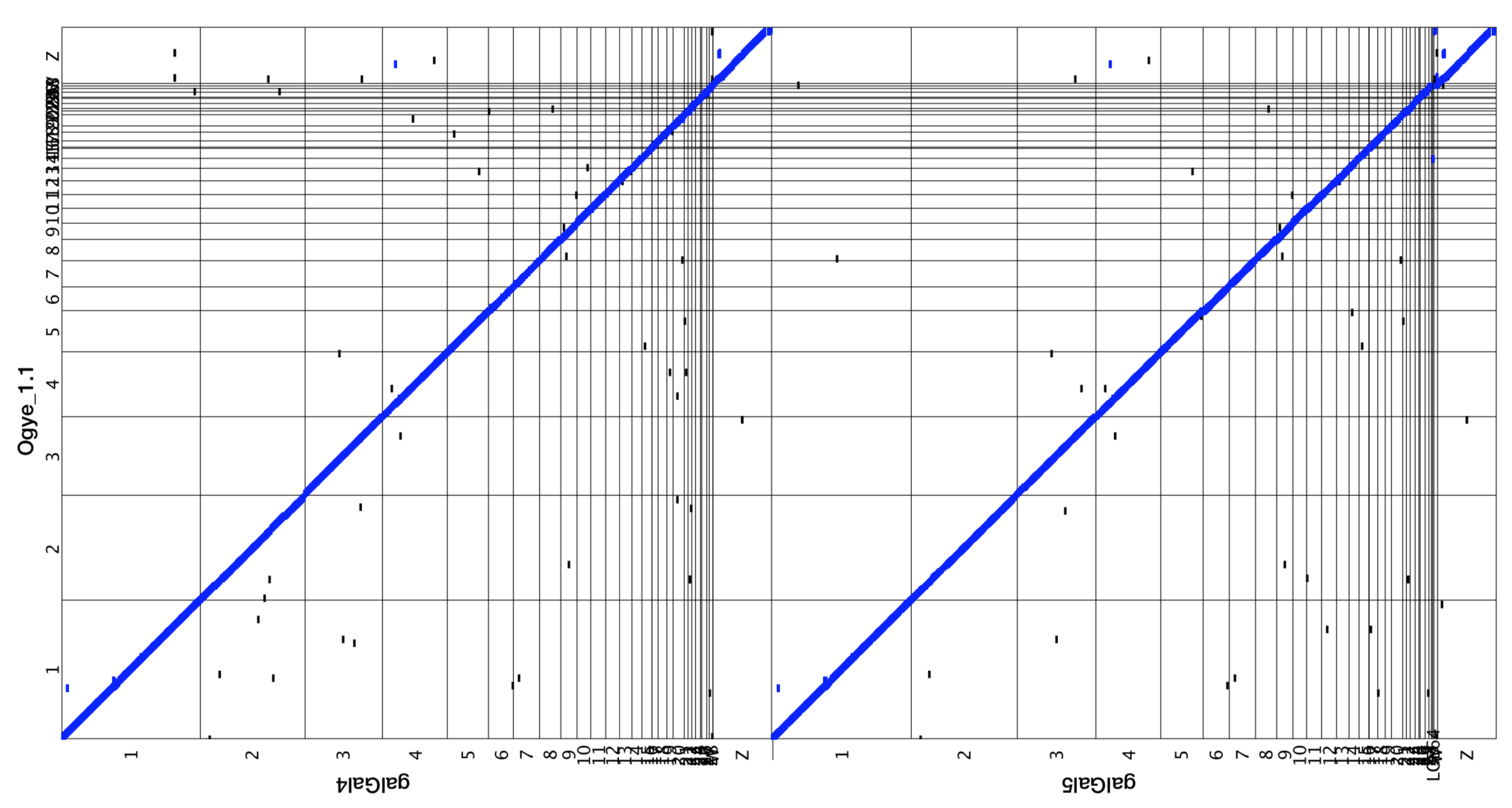


**Figure S4.** Alignment of the Ogye_1.1 genome to galGal4/5 drawn by MUMmer.


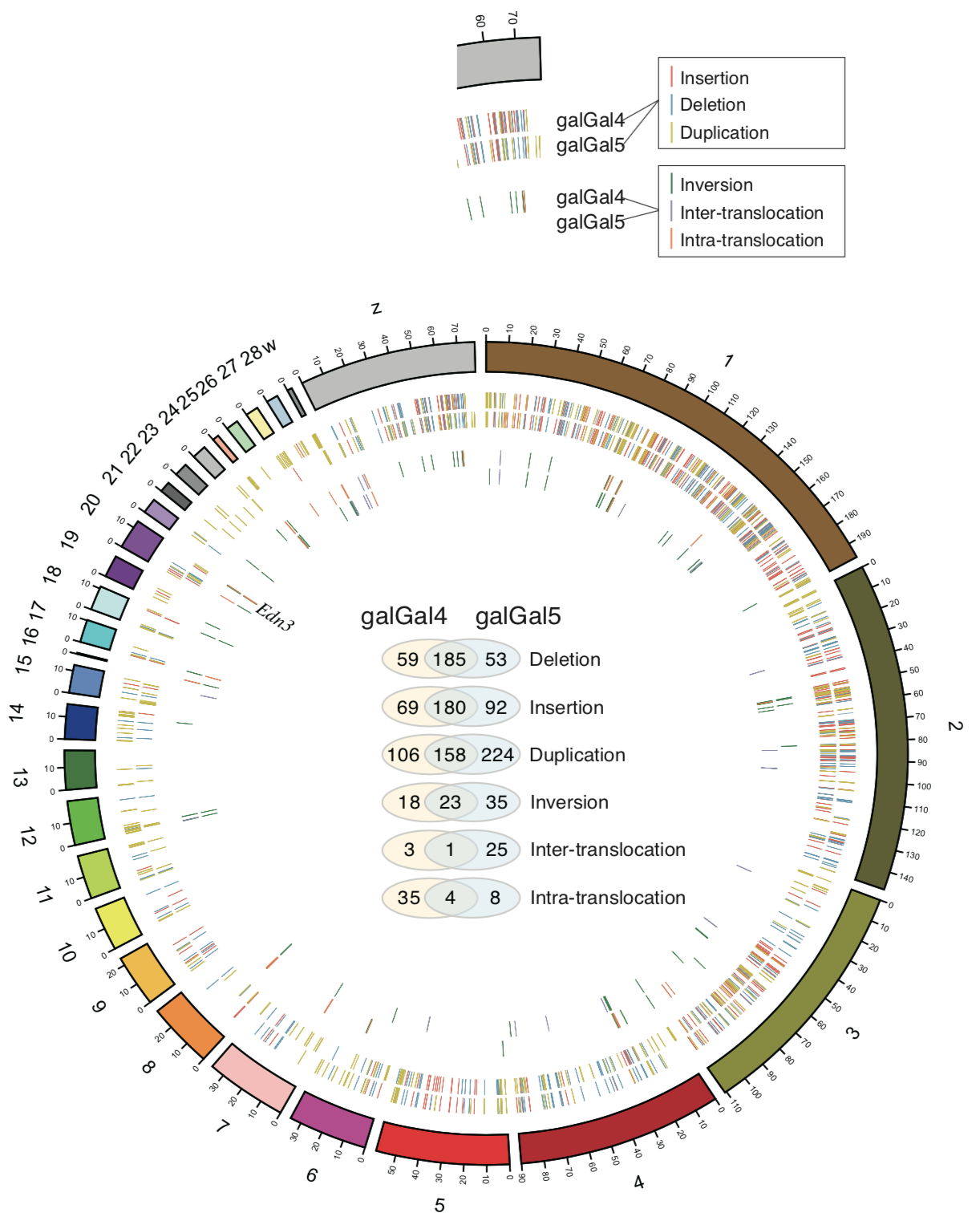


**Figure S5**. Structural variation (SV) map of the Ogye_1.1 genome compared with galGal4 and galGal5. Insertions (red), deletions (blue), duplications (yellow), inversions (green), inter-chromosomal translocations (gray; Inter-translocation), and intra-chromosomal translocations (orange; Intra-translocation) are shown. SVs between the Ogye_1.1 genome and galGal4 or 5 are shown with Venn diagrams.


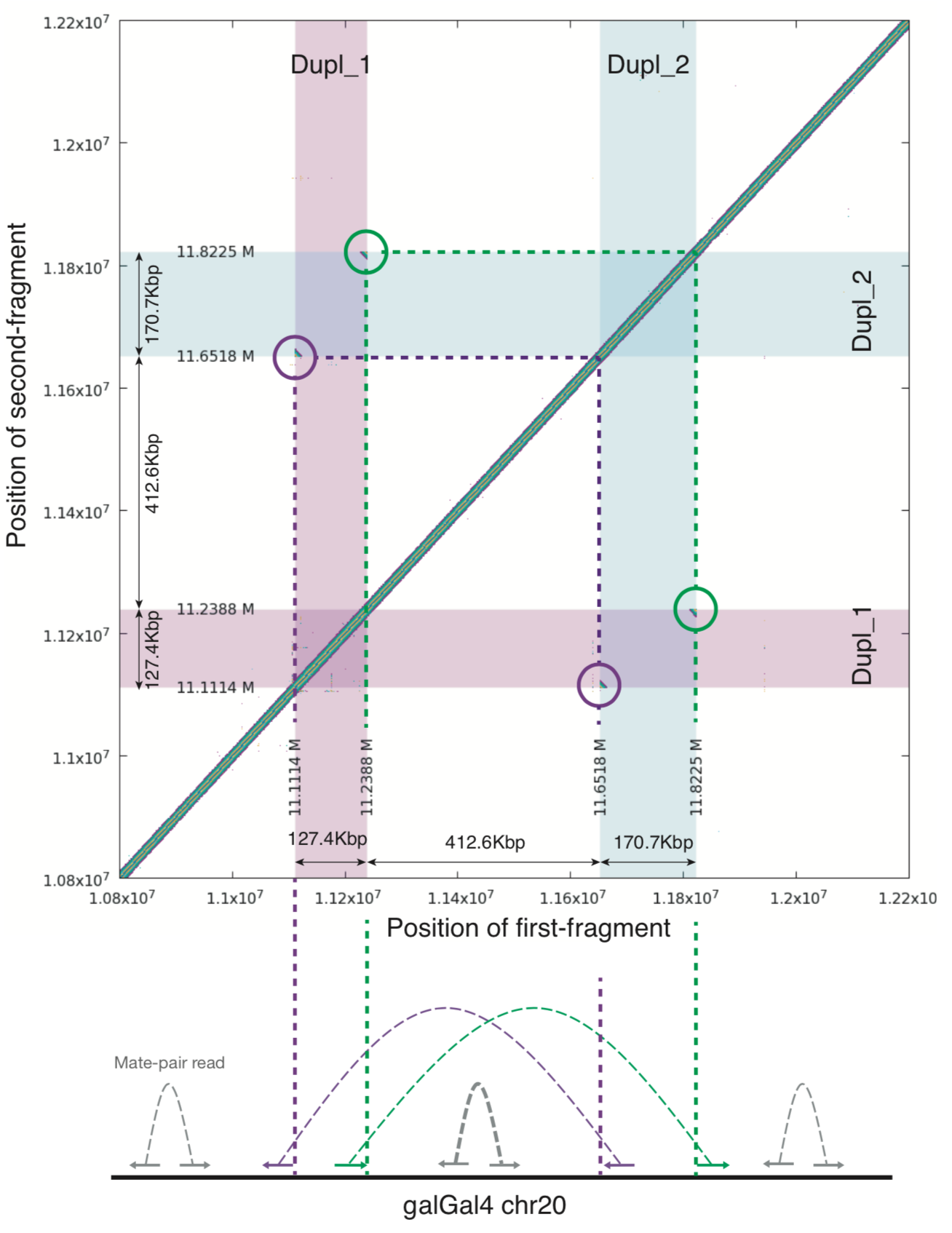


**Figure S6.** Mapping positions of mate-pair reads in the *FM* locus. The x- and y-axes indicate the positions of the first- and second-fragments, respectively, of a mate-pair read (insert size 3-10Kbp). The distance between the positions is the insert size of a mate-pair read.


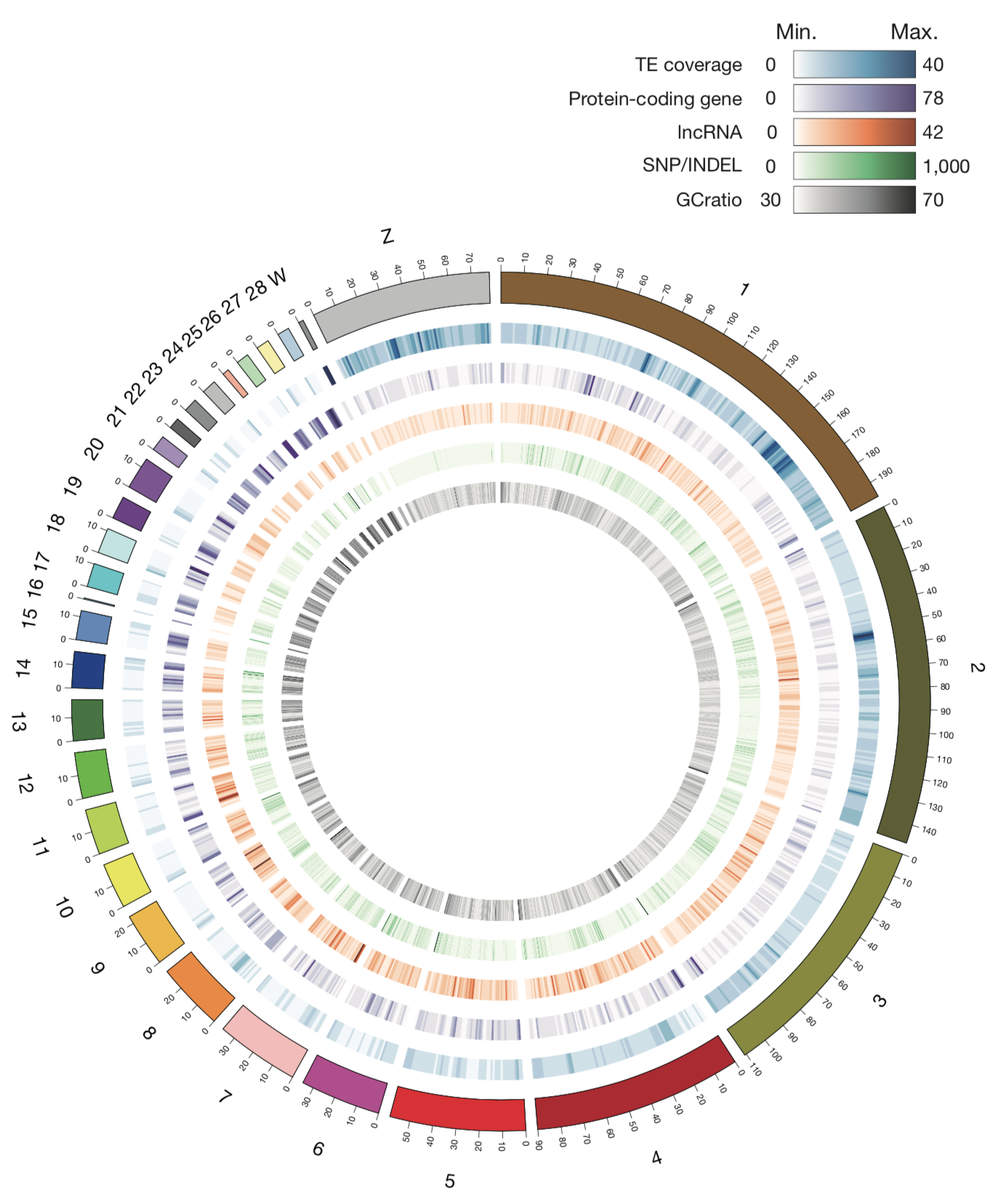


**Figure S7.** Gene (protein-coding and lncRNA) annotation maps of the Ogye_1.1 genome with TE, SNV/INDEL, and GC ratio landscapes shown in a Circos plot. Color codes indicate coverage (%) of TE in a Mbp window, the number of protein-coding genes in a Mbp window, the number of lncRNAs in a Mbp window, SNP and INDEL frequencies in a 100Kbp window, and the GC ratio in a 100Kbp window.

**
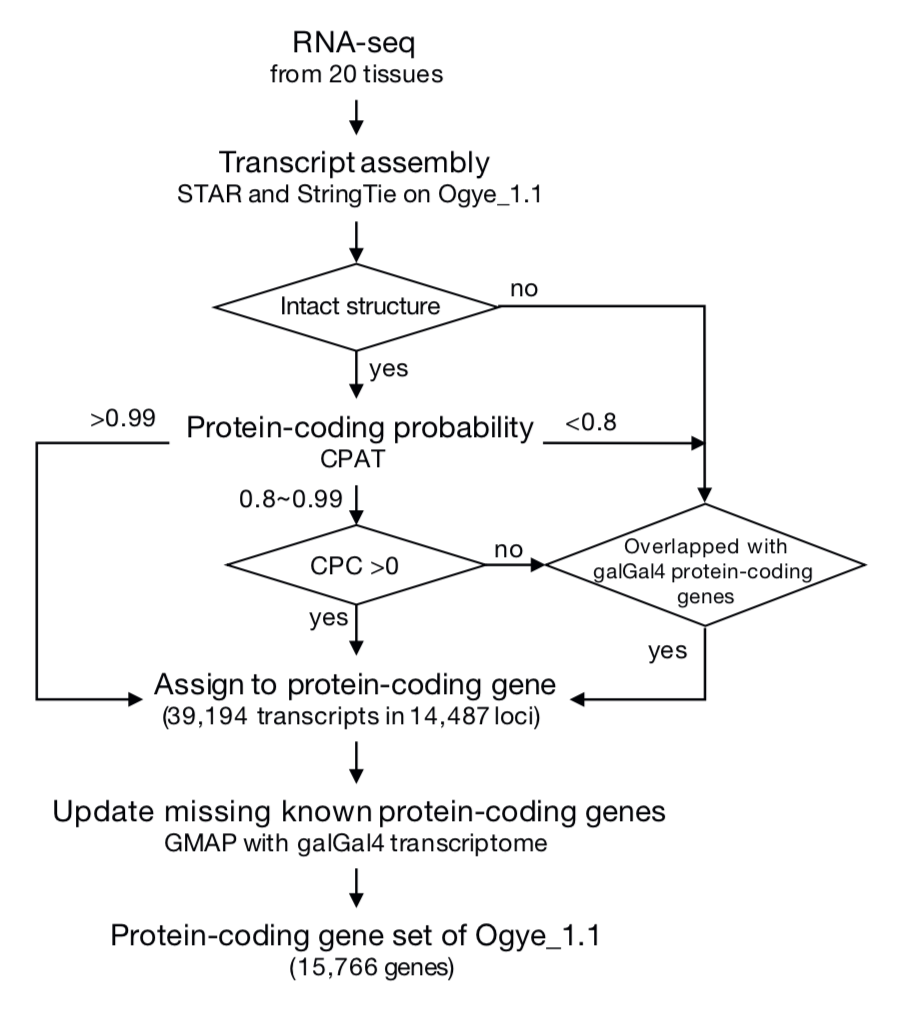
**

**Figure S8.** A schematic flow of our protein-coding gene annotation pipeline.


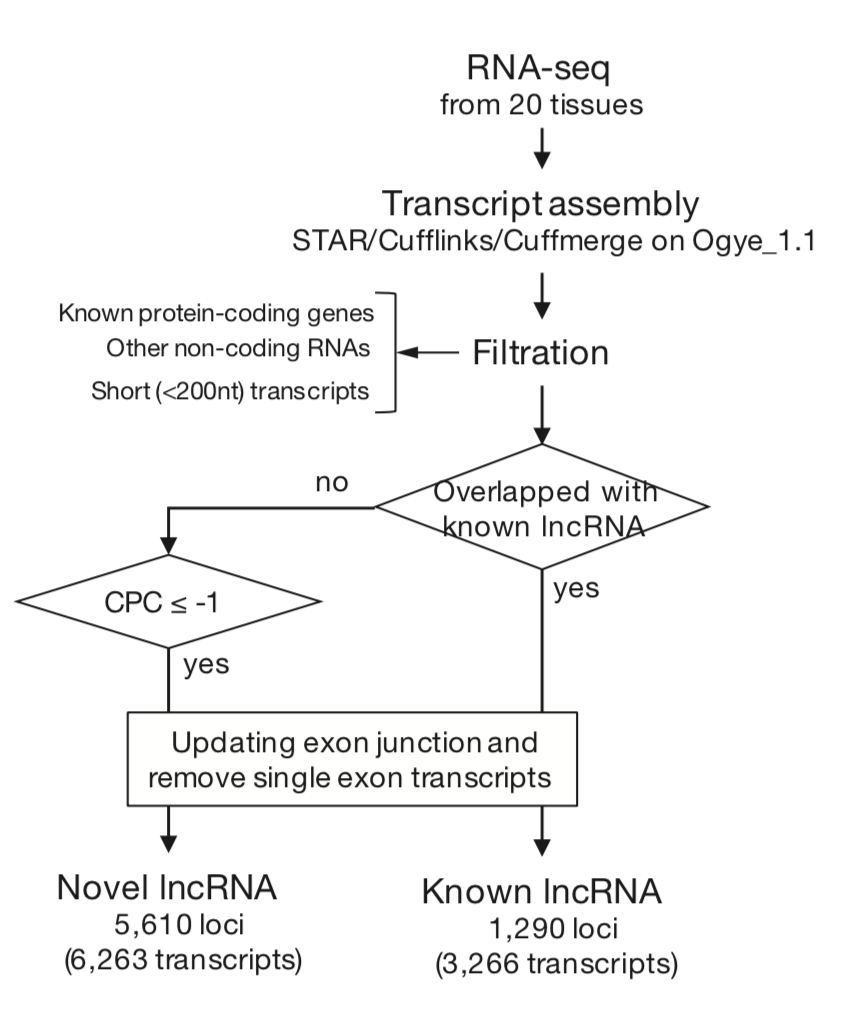


**Figure S9.** A computational pipeline for lncRNA annotations.


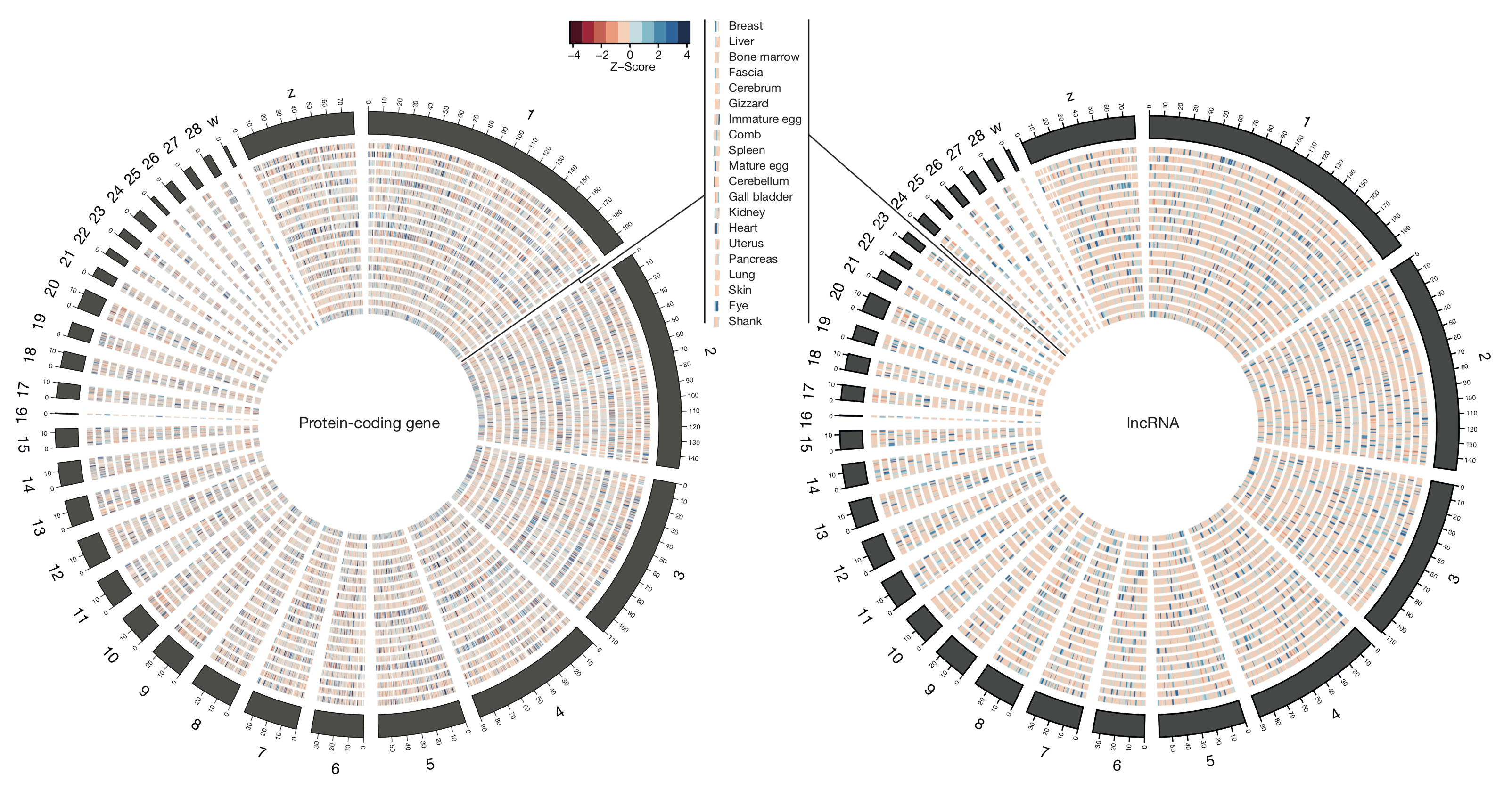


**Figure S10.** Circos plots illustrating the expression levels of protein-coding genes (bottom) and lncRNAs (top) across twenty tissues. The expression levels are indicated with a color-coded Z-score, described in the key.


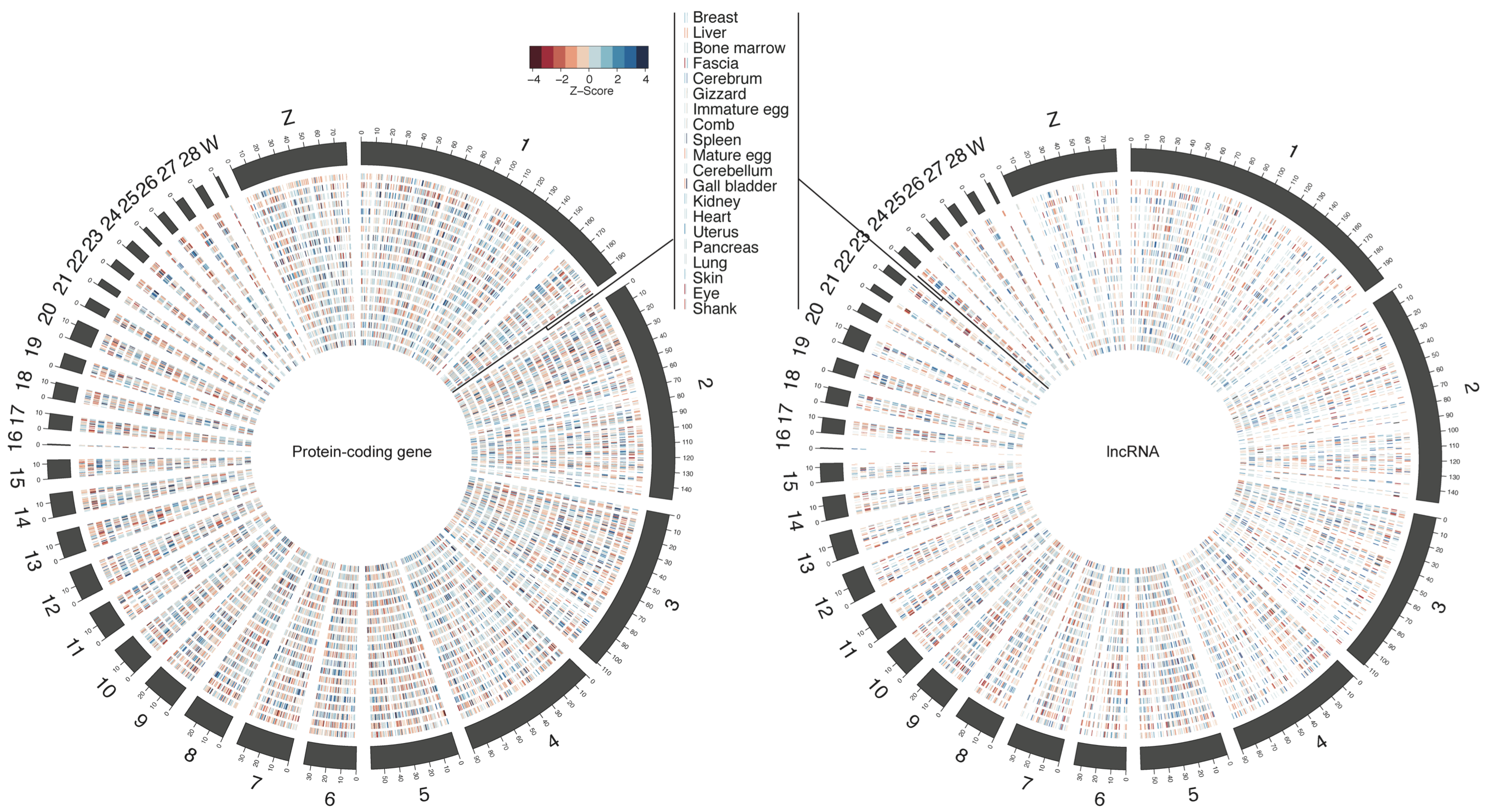


**Figure S11.** Circos plots illustrating the CpG methylation levels in the promoters of protein-coding genes (bottom) and lncRNAs (top) across twenty tissues. The methylation levels are indicated with a color-coded Z-score, described in the key.

**Supplementary tables**

**Table S1.** Statistics of whole genome sequencing data (Illumina) after quality control.

| Sample | Raw data | | | Preprocessed | | |
| --- | --- | --- | --- | --- | --- | --- |
|  | No. of reads | Total base | Coverage(X) | No. of reads | Total base | Coverage(X) |
| 280-1 | 259,169,174 | 38,974,271,145 | 31.2 | 203,503,894 | 26,727,683,533 | 21.4 |
| 280-2 | 248,921,470 | 37,432,679,955 | 29.9 | 194,997,696 | 25,598,021,364 | 20.5 |
| 500-1 | 87,147,858 | 13,105,419,526 | 10.5 | 47,257,894 | 5,981,556,888 | 4.8 |
| 500-2 | 94,391,654 | 14,192,916,731 | 11.4 | 50,923,818 | 6,438,716,723 | 5.2 |
| 500-3 | 28,050,852 | 4,219,596,503 | 3.4 | 12,713,258 | 1,592,996,695 | 1.3 |
| 500-4 | 28,262,138 | 4,251,103,856 | 3.4 | 12,353,670 | 1,545,646,893 | 1.2 |
| 500-5 | 29,154,970 | 4,386,171,517 | 3.5 | 17,618,628 | 2,267,648,617 | 1.8 |
| 500-6 | 57,414,490 | 8,638,408,123 | 6.9 | 42,727,718 | 5,596,779,517 | 4.5 |
| 3kb-1 | 293,080,240 | 43,648,633,694 | 34.9 | 226,125,092 | 29,516,368,816 | 23.6 |
| 3kb-2 | 269,991,204 | 40,178,239,029 | 32.1 | 206,972,694 | 26,972,917,150 | 21.6 |
| 5kb-1 | 229,622,336 | 34,217,282,820 | 27.4 | 163,415,130 | 21,164,214,443 | 16.9 |
| 5kb-2 | 212,826,980 | 31,710,240,640 | 25.4 | 151,615,034 | 19,666,202,244 | 15.7 |
| 8kb-1 | 273,142,796 | 40,718,100,846 | 32.6 | 194,643,442 | 25,265,789,407 | 20.2 |
| 8kb-2 | 270,540,782 | 40,395,881,742 | 32.3 | 189,846,774 | 24,650,814,519 | 19.7 |
| 10kb-1 | 338,205,472 | 50,417,612,062 | 40.3 | 255,648,224 | 33,393,690,677 | 26.7 |
| 10kb-2 | 315,883,674 | 47,093,639,377 | 37.7 | 241,836,614 | 31,564,323,145 | 25.3 |
| FOSMID | 169,900,872 | 17,159,988,274 | 13.7 | 144,432,670 | 13,385,123,915 | 10.7 |
| Total | 3,205,706,962 | 470,740,185,840 | 376.6 | 2,356,632,250 | 301,328,494,546 | 241.1 |

**Table S2.** Structural variations in the Ogye_1.1 genome

| Structural variations to galGal4 | | | | | | |
| --- | --- | --- | --- | --- | --- | --- |
|  | INS | DEL | DUP | INV | TRA | Used libraries or additional information |
| Lastz | 428 | 314 | 878 | 53 | 378 | Detected by genome-to-genome alignment |
| DELLY | 11,387 | 4,558 | 8,149 | 48,746 | 19,341 | PE-280bp/500bp and MP-3Kbp/5Kbp/8Kbp/10Kbp |
| Lumpy | - | 824 | 484 | 26 | 11,076 | PE-500bp |
| FermiKit | 0 | 144 | - | - | 168 | PE-280bp |
| novoBreak | - | 574 | 587 | 238 | 3,742 | PE-280bp (galGal4 data were simulated) |
| Predicted | 249 | 244 | 264 | 41 | 43 | (TRA) 3 Inter-chromosomal and 35 Intra-chromosomal |
|  |  |  |  |  |  |  |
| Structural variations to galGal5 | | | | | | |
|  | INS | DEL | DUP | INV | TRA | Used libraries or additional information |
| Lastz | 431 | 316 | 993 | 125 | 412 | Detected by genome-to-genome alignment |
| DELLY | 11,061 | 6,609 | 8,861 | 21,922 | 4,437 | PE-280bp/500bp and MP-3Kbp/5Kbp/8Kbp/10Kbp |
| Lumpy | - | 702 | 319 | 28 | 3,462 | PE-500bp |
| FermiKit | 0 | 144 | - | - | 276 | PE-280bp |
| novoBreak | - | 554 | 553 | 172 | 15,119 | PE-280bp (galGal4 data were simulated) |
| Predicted | 272 | 238 | 382 | 58 | 48 | (TRA) 3 Inter-chromosomal and 35 Intra-chromosomal |
|  |  |  |  |  |  |  |
| Common set of validated SVs | | | | | | |
|  | INS | DEL | DUP | INV | TRA | Used libraries or additional information |
| Final SVs | 180 | 185 | 158 | 23 | 5 | (TRA) 1 Inter-chromosomal and 4 Intra-chromosomal |

‡ PE and MP are paired-end and mate-pair reads, respectively.

**Table S3.** Repeats in the Ogye_1.1 genome

| Category | Count | Length (bp) | Percentage (%) |
| --- | --- | --- | --- |
| Retroelements | 205,684 | 76,705,032 | 7.65 |
| SINEs | 3,324 | 435,850 | 0.04 |
| Penelope | 80 | 17,950 | 0.00 |
| LINEs | 177,628 | 64,278,215 | 6.41 |
| L2/CR1/Rex | 177,502 | 64,248,756 | 6.41 |
| L1/CIN4 | 46 | 11,509 | 0.00 |
| LTR elements | 24,732 | 11,990,967 | 1.20 |
| Retroviral | 24,630 | 11,972,214 | 1.19 |
| DNA transposons | 27,348 | 9,473,716 | 0.94 |
| hobo-Activator | 13,624 | 5,259,404 | 0.52 |
| Tc1-IS630-Pogo | 5,881 | 2,998,419 | 0.30 |
| Tourist/Harbinger | 2,330 | 215,367 | 0.02 |
| Small RNA | 1,271 | 167,110 | 0.02 |
| Satellites | 767 | 123,194 | 0.01 |
| Simple repeats | 7,721 | 1,224,268 | 0.12 |
| Low complexity | 298 | 56,593 | 0.01 |
| Unclassified | 1,820 | 330,663 | 0.03 |

**Table S4.** Repeat composition in different assemblies.

|  | Mammalian | | | | Reptile | Amphibian | Avian | | | | | |
| --- | --- | --- | --- | --- | --- | --- | --- | --- | --- | --- | --- | --- |
| Repeat Type | Human  (GRCh38) | Human  (GRCh37.p13) | House mouse  (GRCm38.p5) | Pig  (Sscrofa11.1) | Western painted turtle  (Chrysemys_picta_bellii-3.0.3) | Tropical clawed frog  (Xenopus_tropicalis_v9.1) | Golden-collard manakin (ASM171598v1) | Zebra_finch  (Taeniopygia_guttata-3.2.4) | Turkey  (turkey_5.0) | Chicken  (Gallus_gallus-4.0) | Chicken  (Gallus_gallus-5.0) | Chicken  (Ogye_1.1) |
| Low complexity | 0.20% | 0.20% | 0.37% | 0.24% | 0.09% | 0.09% | 0.23% | 0.23% | 0.21% | 0.23% | 0.25% | 0.25% |
| Simple repeats | 1.18% | 1.21% | 2.62% | 1.23% | 0.47% | 0.84% | 0.91% | 1.00% | 0.88% | 1.10% | 1.22% | 1.10% |
| Satellites | 0.38% | 2.46% | 0.15% | 0.13% | 0.00% | 1.71% | 0.00% | 0.00% | 0.01% | 0.20% | 0.21% | 0.01% |
| DNA transposons | 3.17% | 3.19% | 0.00% | 2.17% | 9.14% | 21.06% | 0.16% | 0.14% | 0.15% | 0.95% | 0.94% | 0.94% |
| LTR elements | 8.29% | 8.34% | 11.39% | 4.33% | 4.23% | 2.14% | 0.70% | 2.65% | 0.21% | 1.24% | 1.45% | 1.20% |
| LINEs | 19.29% | 19.42% | 19.09% | 19.84% | 9.29% | 5.17% | 4.40% | 3.07% | 5.30% | 6.44% | 6.47% | 6.41% |
| SINEs | 12.19% | 12.26% | 6.89% | 13.94% | 1.20% | 0.27% | 0.07% | 0.06% | 0.04% | 0.04% | 0.04% | 0.04% |
| Unclassified | 0.16% | 0.16% | 0.35% | 0.03% | 0.57% | 0.83% | 0.03% | 0.03% | 0.03% | 0.03% | 0.03% | 0.03% |
| Total | 44.86% | 47.24% | 40.86% | 41.91% | 24.99% | 32.11% | 6.50% | 7.18% | 6.83% | 10.23% | 10.61% | 9.98% |

**Table S5.** 164 galGal4 protein-coding genes missed in the Ogye_1.1 protein-coding gene annotations.

| ENSEMBL gene_id | Position (chr:start-end in galGal4) | FPKM |  |
| --- | --- | --- | --- |
| ENSGALG00000028531 | 1:533348-534498 | 3.26 |  |
| ENSGALG00000027946 | 2:309959-322560 | 0.23 |  |
| ENSGALG00000028027 | 5:162544-163912 | 30.92 |  |
| ENSGALG00000017675 | 5:199552-201194 | 13.11 |  |
| ENSGALG00000024418 | 5:390379-391322 | 8.55 |  |
| ENSGALG00000029181 | 5:16306938-16308177 | 0.90 |  |
| ENSGALG00000028884 | 5:16308344-16309236 | 0.17 |  |
| ENSGALG00000028451 | 11:2110515-2112244 | 56.75 |  |
| ENSGALG00000026172 | 11:17893779-17895490 | 6.43 |  |
| ENSGALG00000028577 | 19:87366-89807 | 1.77 |  |
| ENSGALG00000027614 | 19:168794-292467 | 16.75 |  |
| ENSGALG00000027059 | 19:5748570-5749048 | 0.33 |  |
| ENSGALG00000024024 | 22:3012325-3020413 | 0.28 |  |
| ENSGALG00000001443 | 23:2715391-2762791 | 2.08 |  |
| ENSGALG00000000443 | 25:205888-214237 | 0.30 |  |
| ENSGALG00000026935 | 25:645944-647516 | 0.19 |  |
| ENSGALG00000026423 | 25:1270841-1272604 | 0.62 |  |
| ENSGALG00000027054 | 25:1672509-1673429 | 0.30 |  |
| ENSGALG00000028854 | 25:1678200-1680026 | 3.39 |  |
| ENSGALG00000028147 | 25:1705617-1707797 | 21.97 |  |
| ENSGALG00000025781 | 25:1746788-1963328 | 7.82 |  |
| ENSGALG00000011948 | 25:1788992-1961542 | 25.65 |  |
| ENSGALG00000026374 | 25:2181544-2183168 | 7.15 |  |
| ENSGALG00000028300 | 26:483316-486297 | 4.27 |  |
| ENSGALG00000027767 | 26:486602-496399 | 1.08 |  |
| ENSGALG00000000417 | 26:1356714-1365537 | 1.18 |  |
| ENSGALG00000025773 | 26:4355463-4356193 | 1.11 |  |
| ENSGALG00000027418 | Z:8951334-8952362 | 2.00 |  |
| ENSGALG00000026058 | Z:65239373-65593260 | 1.05 |  |
| ENSGALG00000025803 | AADN03011344.1:21-1575 | 1.54 |  |
| ENSGALG00000010632 | AADN03012068.1:359-1409 | 4.33 |  |
| ENSGALG00000028472 | AADN03012105.1:21-3500 | 6.27 |  |
| ENSGALG00000027892 | AADN03012277.1:21-1570 | 6.21 |  |
| ENSGALG00000027888 | AADN03012308.1:21-1239 | 8.58 |  |
| ENSGALG00000026691 | AADN03012350.1:3817-9420 | 0.38 |  |
| ENSGALG00000026226 | AADN03012371.1:21-1942 | 5.56 |  |
| ENSGALG00000027846 | AADN03012416.1:4087-9306 | 0.49 |  |
| ENSGALG00000028695 | AADN03012562.1:21-3502 | 0.33 |  |
| ENSGALG00000026539 | AADN03013001.1:223-1462 | 0.40 |  |
| ENSGALG00000026950 | AADN03013269.1:21-1027 | 0.44 |  |
| ENSGALG00000028948 | AADN03013343.1:21-2019 | 0.39 |  |
| ENSGALG00000027006 | AADN03013594.1:21-1610 | 0.52 |  |
| ENSGALG00000027271 | AADN03013954.1:21-1511 | 0.39 |  |
| ENSGALG00000025835 | AADN03013955.1:21-1028 | 0.75 |  |
| ENSGALG00000027557 | AADN03014390.1:24-4048 | 4.78 |  |
| ENSGALG00000026786 | AADN03014468.1:21-1148 | 0.24 |  |
| ENSGALG00000026183 | AADN03014486.1:21-984 | 0.66 |  |
| ENSGALG00000028112 | AADN03014578.1:21-2801 | 17.53 |  |
| ENSGALG00000022278 | AADN03014788.1:1417-3551 | 4.44 |  |
| ENSGALG00000026502 | AADN03015652.1:21-1214 | 3.02 |  |
| ENSGALG00000028109 | AADN03015797.1:332-1819 | 0.91 |  |
| ENSGALG00000029060 | AADN03015811.1:21-2708 | 2.00 |  |
| ENSGALG00000000439 | AADN03016096.1:41-1287 | 14.62 |  |
| ENSGALG00000027227 | AADN03016467.1:7-787 | 111.87 |  |
| ENSGALG00000028248 | AADN03016962.1:82-996 | 2.11 |  |
| ENSGALG00000026895 | AADN03017070.1:21-2365 | 0.46 |  |
| ENSGALG00000028510 | AADN03017184.1:1012-5624 | 0.91 |  |
| ENSGALG00000027150 | AADN03017613.1:21-2489 | 0.30 |  |
| ENSGALG00000027182 | AADN03017869.1:21-1246 | 1.21 |  |
| ENSGALG00000026872 | AADN03018455.1:78-3210 | 0.53 |  |
| ENSGALG00000028844 | AADN03018792.1:21-1947 | 3.84 |  |
| ENSGALG00000029054 | AADN03019163.1:21-1275 | 2.34 |  |
| ENSGALG00000026634 | AADN03019241.1:331-1357 | 37.55 |  |
| ENSGALG00000028909 | AADN03020043.1:21-1154 | 1.19 |  |
| ENSGALG00000026690 | AADN03020052.1:21-1006 | 0.21 |  |
| ENSGALG00000028987 | AADN03020085.1:21-1396 | 0.33 |  |
| ENSGALG00000026731 | AADN03020141.1:21-1154 | 0.94 |  |
| ENSGALG00000028943 | AADN03020792.1:1609-4417 | 2.45 |  |
| ENSGALG00000026330 | AADN03020983.1:21-2249 | 0.58 |  |
| ENSGALG00000027780 | AADN03021099.1:1165-5596 | 3.51 |  |
| ENSGALG00000028099 | AADN03021283.1:875-2576 | 0.64 |  |
| ENSGALG00000028171 | AADN03021284.1:33-1565 | 0.84 |  |
| ENSGALG00000026852 | AADN03021535.1:21-1050 | 66.35 |  |
| ENSGALG00000025837 | AADN03021576.1:2036-3262 | 0.12 |  |
| ENSGALG00000029128 | AADN03021649.1:21-1539 | 0.42 |  |
| ENSGALG00000026988 | AADN03021916.1:21-1175 | 4.42 |  |
| ENSGALG00000028166 | AADN03022079.1:21-1481 | 2.28 |  |
| ENSGALG00000028750 | AADN03022145.1:191-2670 | 1.54 |  |
| ENSGALG00000025932 | AADN03022286.1:21-1405 | 0.15 |  |
| ENSGALG00000027062 | AADN03022443.1:21-1029 | 0.13 |  |
| ENSGALG00000026441 | AADN03022451.1:1699-2853 | 0.25 |  |
| ENSGALG00000026819 | AADN03022582.1:401-1247 | 0.44 |  |
| ENSGALG00000022512 | AADN03022745.1:21-1265 | 3.05 |  |
| ENSGALG00000027042 | AADN03023288.1:470-3466 | 12.30 |  |
| ENSGALG00000026626 | AADN03023623.1:21-5060 | 1.21 |  |
| ENSGALG00000028163 | AADN03024083.1:21-1598 | 0.26 |  |
| ENSGALG00000028427 | AADN03024303.1:164-2979 | 1.06 |  |
| ENSGALG00000028408 | AADN03024512.1:21-1022 | 0.31 |  |
| ENSGALG00000018401 | AADN03024964.1:518-1148 | 5.63 |  |
| ENSGALG00000026223 | AADN03025157.1:1107-2052 | 0.47 |  |
| ENSGALG00000027493 | AADN03025475.1:5071-6900 | 0.23 |  |
| ENSGALG00000028753 | AADN03025573.1:1868-4299 | 6.41 |  |
| ENSGALG00000027618 | AADN03025781.1:37-1229 | 1.20 |  |
| ENSGALG00000027063 | AADN03025956.1:21-2450 | 0.10 |  |
| ENSGALG00000027807 | AADN03026552.1:21-1457 | 38.54 |  |
| ENSGALG00000027081 | AADN03026992.1:2404-7872 | 0.18 |  |
| ENSGALG00000026053 | JH375168.1:43794-45116 | 0.11 |  |
| ENSGALG00000028624 | JH375216.1:3231-5454 | 0.17 |  |
| ENSGALG00000029155 | JH375217.1:202-1990 | 2.18 |  |
| ENSGALG00000028028 | JH375488.1:21-4024 | 0.31 |  |
| ENSGALG00000027798 | JH375501.1:5697-7027 | 0.94 |  |
| ENSGALG00000009600 | JH375536.1:1399-8911 | 1.75 |  |
| ENSGALG00000027912 | JH375572.1:628-2139 | 3.06 |  |
| ENSGALG00000027098 | JH375572.1:3249-4460 | 5.42 |  |
| ENSGALG00000027754 | JH375576.1:3470-4650 | 0.71 |  |
| ENSGALG00000027725 | JH375599.1:21-3727 | 1.55 |  |
| ENSGALG00000018877 | JH375612.1:1140-3888 | 1.17 |  |
| ENSGALG00000028589 | JH375617.1:21-6707 | 3.98 |  |
| ENSGALG00000026992 | JH375620.1:21-4418 | 0.84 |  |
| ENSGALG00000027582 | JH375631.1:21-4759 | 1.00 |  |
| ENSGALG00000025973 | JH375637.1:21-1619 | 0.39 |  |
| ENSGALG00000027878 | JH375637.1:9182-10876 | 0.87 |  |
| ENSGALG00000028630 | JH375643.1:3865-5037 | 0.73 |  |
| ENSGALG00000022438 | JH375652.1:1519-4952 | 0.50 |  |
| ENSGALG00000028752 | JH375682.1:21-1479 | 0.35 |  |
| ENSGALG00000029058 | JH375692.1:3592-8361 | 0.24 |  |
| ENSGALG00000028633 | JH375701.1:21-1625 | 0.13 |  |
| ENSGALG00000027105 | JH375704.1:21-1913 | 0.17 |  |
| ENSGALG00000028618 | JH375751.1:4835-5398 | 6.46 |  |
| ENSGALG00000027361 | JH375875.1:42-1729 | 0.44 |  |
| ENSGALG00000027921 | JH375951.1:21-1274 | 0.20 |  |
| ENSGALG00000028235 | JH375951.1:15563-20591 | 0.82 |  |
| ENSGALG00000028509 | JH375960.1:3260-5509 | 0.61 |  |
| ENSGALG00000014063 | JH376153.1:5934-6856 | 0.25 |  |
| ENSGALG00000000043 | JH376253.1:2868-4211 | 2.81 |  |
| ENSGALG00000022191 | JH376257.1:71-1452 | 7.58 |  |
| ENSGALG00000028724 | JH376257.1:2550-4829 | 13.27 |  |
| ENSGALG00000026855 | JH376272.1:2563-4296 | 0.42 |  |
| ENSGALG00000026114 | JH376279.1:165667-167199 | 4.69 |  |
| ENSGALG00000017621 | JH376309.1:26452-29653 | 1.35 |  |
| ENSGALG00000028353 | JH376400.1:6371-50617 | 0.15 |  |
| ENSGALG00000028859 | 10:1932504-1933601 | 0.06 | (<0.1) |
| ENSGALG00000028962 | 16:223503-225021 | 0.02 | (<0.1) |
| ENSGALG00000027079 | 25:1763855-1764707 | 0.00 | (<0.1) |
| ENSGALG00000029052 | 25:1915046-1918752 | 0.08 | (<0.1) |
| ENSGALG00000025867 | 26:1272816-1273712 | 0.00 | (<0.1) |
| ENSGALG00000026122 | 28:3038847-3039035 | 0.00 | (<0.1) |
| ENSGALG00000025736 | W:4704-10272 | 0.05 | (<0.1) |
| ENSGALG00000028874 | AADN03012450.1:509-1642 | 0.02 | (<0.1) |
| ENSGALG00000026403 | AADN03013310.1:859-1959 | 0.00 | (<0.1) |
| ENSGALG00000029111 | AADN03013745.1:21-1805 | 0.01 | (<0.1) |
| ENSGALG00000028712 | AADN03013871.1:21-1002 | 0.02 | (<0.1) |
| ENSGALG00000028474 | AADN03014119.1:21-1098 | 0.09 | (<0.1) |
| ENSGALG00000028613 | AADN03014174.1:2939-5496 | 0.04 | (<0.1) |
| ENSGALG00000027942 | AADN03016208.1:21-1037 | 0.05 | (<0.1) |
| ENSGALG00000027563 | AADN03016590.1:21-1322 | 0.08 | (<0.1) |
| ENSGALG00000029010 | AADN03016854.1:21-1748 | 0.05 | (<0.1) |
| ENSGALG00000028776 | AADN03017194.1:21-1331 | 0.06 | (<0.1) |
| ENSGALG00000028160 | AADN03020473.1:21-1839 | 0.01 | (<0.1) |
| ENSGALG00000027911 | AADN03021714.1:39-1330 | 0.04 | (<0.1) |
| ENSGALG00000027755 | AADN03022644.1:21-1298 | 0.02 | (<0.1) |
| ENSGALG00000025824 | AADN03022943.1:2252-4058 | 0.01 | (<0.1) |
| ENSGALG00000029067 | AADN03025573.1:435-1787 | 0.01 | (<0.1) |
| ENSGALG00000018582 | AADN03025979.1:1328-3394 | 0.02 | (<0.1) |
| ENSGALG00000026967 | AADN03026431.1:437-2170 | 0.07 | (<0.1) |
| ENSGALG00000025942 | JH375454.1:13888-15359 | 0.02 | (<0.1) |
| ENSGALG00000028959 | JH375862.1:5286-7377 | 0.03 | (<0.1) |
| ENSGALG00000027701 | JH375862.1:9830-10903 | 0.05 | (<0.1) |
| ENSGALG00000028229 | JH375914.1:21-4200 | 0.06 | (<0.1) |
| ENSGALG00000027676 | JH376001.1:4459-6632 | 0.04 | (<0.1) |
| ENSGALG00000025914 | JH376016.1:2943-7169 | 0.00 | (<0.1) |
| ENSGALG00000022280 | JH376151.1:5010-6537 | 0.00 | (<0.1) |
| ENSGALG00000026451 | JH376158.1:7261-7804 | 0.00 | (<0.1) |
| ENSGALG00000026115 | JH376277.1:1876-2976 | 0.01 | (<0.1) |
