# Supplementary material for: Whole genome and transcriptome maps of the entirely black native Korean chicken breed Yeonsan Ogye
Source: Gigascience. 2018 Jul 11;7(7):giy086. doi: 10.1093/gigascience/giy086 (PMC6065499; doi:10.1093/gigascience/giy086)
Supplement: Response_to_Reviewer_Comments_Original_Submission.pdf [file giy086_response_to_reviewer_comments_original_submission.pdf]

Author's Response To Reviewer Comments

Close

May 18, 2018

Dear Dr. Hans Zauner, and reviewers.

Thank you for sending the referee reports and inviting us to submit a revised manuscript. Please find in the accompanying files our manuscript, "Whole genome and transcriptome maps of the entirely black native Korean chicken breed Yeonsan Ogye" by Jang-il Sohn, Kyoungwoo Nam, Hyosun Hong, Jun-Mo Kim, Dajeong Lim, Kyung-Tai Lee, Yoon Jung Do, Chang Yeon Cho, Namshin Kim, Han-Ha Chai, and Jin-Wu Nam, for consideration as a Data Note in GigaScience.

We appreciate the reviewers' many helpful comments and insights, which have led to substantial improvements to our paper. We have addressed all of the concerns of the referees, as detailed in our point-by-point response to their comments. Based on your helpful comments, our manuscript has been substantially improved.

We have attached CoverLetter and Point-by-point response files as response letters for editor and reviewers, respectively. The same response, written here, have been written in the files with tables and figures. Please find the files in attached files.

We hope that we have adequately responded to the reviewers' inquiries and that you will find our revised manuscript suitable for publication in GigaScience.

Sincerely,

Jin-Wu Nam
Department of Life Science, College of Nature Sciences, Hanyang University, Seoul 133791, Republic of Korea
Telephone: +82-2-2220-2428
Fax: +82-2-2298-0319
jwnam@hanyang.ac.kr

==

* Comments are repeated with numbering, and our detailed responses are written below each comment. Every detailed response begins with "A:" and ends with dashes, "---------".

Response to editor

1) Reviewer 2 mentions a number of points that should be discussed at least briefly in the manuscript (e.g. why Gal4 and not Gal5 was used, and a question (point 5) regarding the scaffolding procedure, among others).

A:
Both reviewers asked why galGal4 was used as a reference instead of galGal5. Because

galGal4 was the most current version of chicken reference genome when we finished our Ogye whole genome assembly, we compared our draft genome to galGal4. Later on, galGal5 was released at NCBI (November 2015) and then published (Warren et al., 2017). We then additionally compared our Ogye draft genome with galGal5 when we analyzed the completeness of the draft genome using BUSCO and structural variations.

Regarding the scaffolding procedure (point 5 of reviewer 2), the scaffold N50 of the initial ALLPATHS-LG assembly was 10.7Mb, which jumped up to 27.8Mb after the second scaffolding using PacBio and Fosmid data (Figure 1B and S1 in the revised manuscript). After breaking mis-assemblies, the N50 then increased to 18.7Mb. Further, we recognized that the word "scaffolding" might be confusing because there are several scaffolding steps in our pipeline. Hence, we have added a detailed description of the scaffolding steps to clarify this point in the revised manuscript on page 6 and 7 and have labeled the second and third scaffolding and gap-filling steps in Figure 1B.

----

2) I feel reviewer 1 is correct that some of the figures could be moved to the supplementary material - we don't have strict rules on the number of figures, but I would suggest to move at least the images demonstrating the pipelines (1B, 4B, 5A) to the supplemental material, to present the manuscript more concisely. Most of our readers are familiar with this type of analysis pipeline, so arguably don't need these figures to understand the work - but helpful to have it in the supplemental material! (But see also comment 2) of reviewer 2 regarding adding more information to Fig 1B - that seems also a good option for this figure).

A:
Based on these suggestions, we have moved Figures 4B and 5A (gene annotation pipelines) from the original manuscript to the supplemental figures in the revised manuscript. Regarding the whole genome assemble pipeline (Figure 1B), we decided to add detailed information to Figure 1B in the revised manuscript, based on the comment from reviewer 2.

----

3) Maybe you would like to also move a couple of the circus plots to the supplemental material as well, but that's up to you really - if you feel strongly about them, you can keep them in the main text.

A:
Following this suggestion, we have moved the Circos plots in Figures 2A, 4A, 6A, and 8A of the original manuscript to the supplemental materials but we kept the Circos plot of Figure 9 in Figure 6F in the revised version.

----

4) Both reviewer have good suggestions to improve reproducibility: I agree with reviewer 1 that a Supplemental file containing an inventory of commands, including runtime options, would be very useful.

A:
We agree with this suggestion. We have added a supplemental material file including all command lines with runtime options that we used in the analyses including whole genome assembly, transcriptome assembly and gene annotations, and so on.

----

5) Reviewer 2 suggests to include a README file for the dataset, including a short description of the data inventory. This is also a good suggestion; prior to publication our data curators will have a look at the GigaDB dataset and they will be in touch before publication, this would then also a good time to include a README file.

A:
According to this suggestion, we have added a README file including data description.
----

6) Finally, I also agree that the article would benefit from a native speaker reading over it once more before submission of the revised version.

A:
Following this suggestion, we have revised the manuscript, which was edited by a native speaker.

Response to reviewer #1:

Dr. Robert Kraus' special comment:
I like the article because it describes a rich and interesting data set of a very interesting chicken breed. We do not only get presented the genome and high quality assembly, but also various transcriptomes and methylomes. This includes not only annotation but also quantitative information and tissue specific expression atlases. I could not find major problems with this article. Analyses seem to be correctly performed and well described.

A:
We are very pleased that Dr. Robert Kraus thought that our study and results include valuable information with respect to not only genome assembly and annotations but also quantitative transcriptome and DNA methylome data. Very useful points were raised about specific details of our methods and descriptions of results and we feel that our manuscript has significantly benefited from making changes in response to all of the reviewer's major and minor comments.

Major comments:
1) During the review process it was hard to refer to places in the text because the lines numbers started over all over on each page; and on top of that, pages were not numbered… I counted page number on page 3 of the document that was available to me because the first two pages were info pages from the editorial office. Page three is the one where the abstract starts. E.g., P1L34 means line 34 on that first page.

A:
We are very sorry for causing the confusion about line numbers and for not including page numbers in the pdf file. In the revised version, we included the appropriate line numbers and page numbers in the main and supplementary manuscripts.
----

2) I further found that the quality of English could be improved. It is OK for now but the

article would really benefit from a native speaker reading over it once more (I am not a native speaker myself, though). Where possible I indicated potential changes to the language but could not be exhaustive.

A:
As the reviewer suggested, we improved the quality of English throughout the manuscript by a commercial English editing service.
----

3) Data availability: I understand that this journal has (luckily!) high standards regarding data availability. The pipelines are well illustrated in the paper and also in the Supp Mat. However, not all runtime parameters are specified for all programmes. Often it is mentioned in the text. I am not sure now if this is sufficient, for a regular journal that does not focus so much on the technicalities I would suggest this is sufficient. For this journal, with its special emphasis on technicalities and bioinformatics protocols, I could imagine that a Supp Mat file containing an inventory of all the command incl. their runtime options would be useful. This may be added at the discretion of the Editor of this journal, if otherwise done regularly here.

A:
We absolutely agree with Robert’s comment. All command lines with runtime options that we used for genome assembly, gene annotation, transcriptome assembly, RNA-seq and DNA methylation profiling are summarized in the file “Supplementary_Command.docx”.

Specific comments
4) P1L34: gallus gallus domesticus -> Gallus gallus domesticus

A:
Thank you for the comment. We have corrected it as the reviewer suggested.
----

5) P1L40: I don't think you should present the definition of N50 in the abstract

A:
As the reviewer suggested, we have removed this definition from the abstract.
----

6) P1L46: The statement about Galgal5 using only long-read technology is not true. It also used previous data to build on - and that includes 1st and 2nd generation sequencing data - plus Illumina (>30x) was used for error correction. Let alone BAC sequencing and the likes.

A:
We sincerely thank the reviewer for this comment. Our intended meaning was that the genome assembly version of galGal5 took advantage of long read data along with other short read data. In detail, according to the manuscript about galGal5 assembly (Warren et al., 2017), contig assembly was done with 50X PacBio long reads, and then the resulting contigs were scaffolded with Fosmid paired-end sequences (3 Kb and 40 Kb insert-sizes) and with BAC end sequences, as the reviewer commented. Hence, we have changed the

description from
“assembled with a long read-only method”
to
“contigs were assembled with high-depth PacBio long-reads (50X) and scaffolded with short reads”
----

7) P3L27: substitute "constructed" with "assembled"

A:
We have changed from “constructed” to “assembled”.
----

8) P3L47: include -> includes

A:
We have changed from “include” to “includes”.
----

9) P4L23: The sequencing library from one bird only or combined?

A:
The sequencing library was obtained from one an individual Yeonsan Ogye. We have found a typo “chickens”, which might have made you confused. Thus, we revised the phrase “8-month-old YO chickens” to “An 8-month-old YO chicken” on page 4 line 1. Additionally, we added a sentence “All sequencing data in this study (including data from whole genome sequencing, RNA-seq, and RRBS) were obtained from this sample bird.” on page 4 line 3. This revised sentence is also related to comments 17 and 18.
----

10) P4L39: The text in the bracket should be changed. It now reads as if most of reads were bad quality. Please clarify whether this was your threshold or observation.

A:
The phrase in the brackets was meant to refer to thresholds for the IlluQC_PRLL.pl module in the NGS QC Toolkit, i.e. options “-l 70 -s 20”, which are default options. Because the phrase was related to the preprocessing step in whole genome assembly, we have moved the phrase to the “Hybrid whole genome assembly” section, and modified a sentence from
“During the preprocessing step, the errors in the Illumina short reads were corrected by KmerFreq and Corrector [12].”
to
“In the preprocessing step, reads in which ≥ 30% of the nucleotides had a Phred score < 20 were excluded using NGS QC Toolkit (IlluQC_PRLL.pl) [36], the adaptor sequences of the remaining reads were removed using Trimmomatic [37], and three nucleotides at the 5’-end and five nucleotides at the 3’-end of the reads were trimmed using NGS QC Toolkit (TrimmingReads.pl). After quality control, the sequencing errors in the Illumina paired-end short-reads were corrected using KmerFreq and Corrector [38].” on page 6 line 6-11,
and from
“In total, 232.2 X Illumina short reads were obtained (59.6 X from the small insert libraries and 172.6 X from the large insert libraries) and, after filtering raw data with low quality (>

30% of the base-pairs in a read have a Phred score <20), 163.5X were used for genome assembly.”
to
“In total, 376.6X raw Illumina short-reads (100.2X from the small insert libraries and 276.4X from the large insert libraries) were generated (Tables 1 and S1)” on page 4 line 14-15.
----

11) P4L41: How do the 163.5x distribute across paired-end and mate-pair data?

A:
As we mentioned above, we updated the coverage of the total preprocessed short reads from 163.5X to 241.1X. Accordingly, we revised Tables 1 and S1 to include detailed information about the DNA sequencing data used for the whole genome assembly and downstream analyses including detailed coverages of paired-end and mate-pair data in the raw and preprocessed steps.
----

12) P4L43: I would not say "scaffold quality", because quality is a complex thing. You could be more specific, and refer to "scaffold N50", or "improve scaffolding".

A:
Thank you for this comment. We have changed to “scaffold N50”.
----

13) P4L43: Which PacBio machine was used and which chemistry/version?

A:
PacBio long reads were produced by the PacBio RS II platform with P6C4 chemistry. Thus, we changed from
“11.5X PacBio long reads were additionally sequenced”
to
“9.7X PacBio long-reads were additionally sequenced using the PacBio RS II platform with P6C4 chemistry”
The coverage was changed to 9.7X due to using the estimated genome size, 1.25Gbp.
----

14) P4L51: A reference should be given to the Trizol protocol/company where the reagent was purchased.

A:
The Trizol was purchased from Sigma-Aldrich. We changed from “TRIzol” to “TRIzol (Sigma-Aldrich®, St. Louis, USA)”.
----

15) P5L20: Unclear what "qualified" means, consider rephrasing

A:
We have rephrased “qualified using the Agilent …” to “the integrity of the cDNA libraries was examined using the Agilent …” to clarify the intended meaning on page 5 line 8-9.

Similarly, we also rephrased “qualified using the Agilent …” to “and the integrity was examined using the Agilent …” on page 5 line 24.
----

16) P5L24: tissues, which are -> tissues;

A:
As the reviewer suggested, we have changed this wording.
----

17) P5L29: All tissues from the same bird? The same bird for which genomic DNA was sequenced?

A:
Our RNA-seq data all comes from the same bird. To clarify this point, we revised the original sentence “… and shank (Table 2).” to “… and shank from the same bird (Table 2).”
----

18) P5L37: Again, was it from the same bird(s) as in the previous steps?

A:
We have RRBS data from twenty tissues from the same bird.
----

19) P5L46: How was ligation assessed?

A:
To avoid an unintended meaning, we have removed the phrase “After ligation had been assessed”.
----

20) P6L25: In hybrid -> In the hybrid

A:
We have changed this wording to “In the hybrid”.
----

21) P6L36: Why not aligned to Galgal5?

A:
As the reviewer pointed, galGal5 is the latest version of the chicken genome assembly. However, because the latest version of the chicken reference genome was galGal4 when we finished our Ogye whole genome assembly, we compared our draft genome to galGal4. Later on, galGal5 was released (November 2015). We then additionally compared our Ogye draft genome with galGal5 when we analyzed structural variations and the completeness of the draft genome using BUSCO.
----

22) P7L26: The mentioning of these stats are the best or second best values is superfluous. Actually, I am sure by now there are better stats for assemblies of zebra finch or

hummingsbird, that were treated with the currently best possible protocols. This doesn't mean your stats are bad, not at all. But the statement does very likely not apply - and surely will not apply anymore within a really short amount of time. Delete it.

A:
We agree with the reviewer's comment and removed the statement.
----

23) P7L49: "by at least a different"; should that be …by at least four different SV prediction programs?

A:
We have changed from "by at least a different" to "by four different", and revised the following sentence, "SVs, validated by at least one program, included" to clarify.
----

24) P8L13: "three possible mechanistic scenarios" -> explain what scenarios

A:
We recognized that the original description and Figure 2B could be misleading; thus, we changed the sentence,
"A previous study suggested that the inverted duplication of the FM locus could be explained by three possible mechanistic scenarios (Figure 2B) [3]."
to
"A previous study, using conventional PCR assays, suggested three possible rearrangements at the FM locus [3]" on page 8 line 19-20.

And we also updated the Figure 2 legend to include a description of the three possible scenarios.

"Figure 2. Three possible scenarios for DNA rearrangements that could lead to the inverted duplication of the Fibromelanosis (FM) locus in the genomes of hyperpigmented chicken breeds; A. Three possible scenarios were developed based on the overlap patterns. The green and red lines indicated two duplicated genomic loci (Dupl_1 and Dupl_2, respectively) including the FM locus. Scenario 1 consists of a one-step rearrangement—an inverted duplication—whereas Scenarios 2 and 3 consist of a simultaneous rearrangement of an inverted duplication and an inversion; B. The read depth of a locus on chromosome 20 is shown in the top panel and the continuous/discontinuous patterns between mapped scaffolds are shown in the bottom panel. The star indicates the pattern that is discontinuous on both sides, validated in C; C. A comparison of the FM locus in galGal4 and the Ogye draft genome with aligned contigs (black lines) in each scaffold. The gray bands indicate the estimated gaps between contigs. The estimated sizes of Gap_1 and Gap_2 are 164.5Kbp and 63.3Kbp, respectively. The purple lines in the box indicate the END3 gene locus and the green and yellow shades indicate the duplicated regions (Dupl_1 and Dupl_2, respectively). The dark green and yellow shades indicate the discontinuous regions between scaffolds."

In addition, we have updated the description in the main text as follow.
"To discern which rearrangement best fits our results, the FM loci from galGal4 and the Ogye draft were compared with the resulting scaffolds, showing an inverted duplication with discontinued scaffolds at both duplicated regions (Figures 2A; C). The results, with a

discontinued scaffold on both sides, support rearrangement 1 rather than rearrangement 2 or 3, which have a discontinued scaffold on only one side. Although rearrangement 1 needs to be further validated, the FM locus in the Ogye_1.1 genome was updated according to the first rearrangement (Figure 2C).” on page 9 line 3-8.
----

25) P8L46: "and other genomes": what other genomes?

A:
We have changed from “Repeat elements in the Ogye_1.1 and other genomes were predicted” to “Repeat elements in the Ogye_1.1 and other genomes (human, mouse, pig, western painted turtle, tropical clawed frog, zebra finch, turkey, and chicken) were predicted” on page 9 line 12-13.
----

26) P9L4: the first word in the first line on this page, "to", should be removed, right?

A:
Thank you for this comment. We have removed the word.
----

27) P9L11: was depicted -> is depicted

A:
We have changed the phrase to “is depicted”.
----

28) P9L19: The number of SNPs seems little. How does this compare to the Galgal 4/5 or other, actually more outbred bird genomes?

A:
To investigate the small numbers of SNPs and INDELs, we checked and found a technical problem in the GATK pipeline that was installed in our system. To fix this problem, SNPs and INDELs were re-identified using VarScan 2 (Koboldt et al., 2012, Genome research), which found 3,206,794 SNPs and 302,463 INDELs. Hence, we have changed the description from
“As a result, 599,326 SNPs and 23,274 insertions/deletions (INDELs) were identified across the genome using Genome Analysis Toolkit (GATK) modules: HaplotypeCaller, combineGVCF, GenotypeGVCFs and VariantFiltration (with options “QD < 2.0 || FS > 200.0 || ReadPosRankSum < -20.0”) [20].”
to
“We identified 3,206,794 SNPs and 302,463 INDELs across the genome using VarScan 2 with options --min-coverage 8 --min-reads2 2 --min-avg-qual 15 --min-var-freq 0.2 --p-value 1e-2 [57].” on page 10 line 2-4.
----

29) P10L11: Where are those 33 genes situated, as evidenced by comparative mapping. Exclusively on microchromosomes?

A:

We have checked where the 33 genes were assigned and found that the majority (26) of them were located on unknown chromosomes. We thus added a sentence as follows:
"Of the 33 missing genes, 26 appeared to be located on unknown chromosomes and the remainder are on autosomes (six genes) or the W sex chromosome (one gene) in galGal4." on page 10 line 23-24.

Additionally, we updated Table S5 (previously S4) to add the genomic positions of the 164 protein-coding genes missed in the Ogye_1.1 gene annotations.
----

30) P10L19: annotated -> annotate

A:
We corrected the word to "annotate".
----

31) P11L29: I am not sure what you mean by "which were dynamically changed". Please explain/rephrase.

A:
To avoid an unintended meaning and confusion, we removed the phrase. Thank you for the comment.
----

32) P12L12: It should be "in the genome" and not "in genomes", right?

A:
We corrected the phrase to "in the genome".
----

33) P12L31: Remove the two commas around "then".

A:
We removed two commas from "We, then, examined" to "We then examined".
----

34) P12L40: "specifically" should be changed to "specific", I think?

A:
We changed from "tissue specifically expressed genes" to "genes with tissue-specific expression".
----

35) P12L42: Should "only" be "one"? This sentence need to be clarified.

A:
We changed the original description to "280 protein-coding and 392 lncRNA genes with expression ≥ 10 FPKM in at least one tissue and with a maximum expression value four-fold higher than the mean expression level in twenty tissues" on page 13 line 11-13.
----

36) P12L53: noises -> noise

A:
We changed the word to “noise”.
----

37) P12L56: taken account -> taken into account

A:
We changed the phrase to “taken into account”.
----

38) P13L5: That should be "Discussion" in singular, right?

A:
We changed the word to “Discussion”.
----

39) P13L25-30: Cite the Lemur study and/or the MaSuRCA assembler

A:
We have cited the Lemur study and MaSuRCA assembler as follows.
“A similar tendency can be seen in the Golden-collared manakin genome (ASM171598v1) (Figure 3), which was also assembled in a hybrid manner using MaSuRCA assembler with high-depth Illumina short reads and low-depth PacBio long reads.”
to
“A similar tendency can be seen in the Golden-collared manakin genome (ASM171598v1) [32] (Figure 3) and the gray mouse lemur genome (Mmur3.0) [77], which were also assembled in a hybrid manner with high-depth Illumina short-reads and low-depth PacBio long-reads.”
including their references on page 14 line 7-9.
----

40) P13L59: Remove "The" at beginning of sentence.

A:
We removed “The” at the beginning of the sentence.
----

41) P14L17: gnome -> genome

A:
Thank you for this comment. We corrected the spelling to “genome”.
----

42) Table 1: You should also include a row that summarises the total of coverage (x) across all PE and mate-pair, and the total all. The numbers are in the text, but should re-appear in the table.

A:
At the reviewer’s suggestion, we added rows to summarize the total coverage (x) of paired-end reads, mate-pair reads, Illumina short-read totals, and the final totals, all in Table 1.
----

43) Table 4: Add a column that gives the species name.

A:
We have added a column giving species names in Table 4.
----

44) Table 5: all "site" should become plural, i.e., "sites".

A:
We changed all “site” entries to the plural “sites” in Table 5.
----

45) Figure 1C: Do I see correctly that is says "Chichen" and not "Chicken" in line 2? Also: Space missing between "Hooded" and "crow". Assemblies mentioned in this table should have footnotes to them and below the table cite the actual scientific reference articles.

A:
We have fixed to “Chicken” and “Hooded crow” in Figure 1C, and added scientific references in the table. We could not, however, find an appropriate reference for the Blue-crowned manakin genome.

Response to reviewer #2:

1) Why wasn't Galgal5 used in the overall analyses? (only Galgal4 used) It was presented in the beginning and end of the manuscript.

A:
galGal5 is the latest version of chicken genome assembly. However, because the latest version of the chicken reference genome was galGal4 when we finished the Ogye whole genome assembly, we compared our draft genome to galGal4. Later on, galGal5 was released at NCBI (November 2015) and published (Warren et al., 2017). We then additionally compared our Ogye draft genome with galGal5 when we analyzed structural variations and the completeness of the draft genome using BUSCO.
----

2) Figure 1B is referenced quite often in the manuscript, however the problem with 1B is that the figure is very simple and not detailed enough. You may want to break up 1B into its own figure, and substitute it with a variation of S1B.

A:
As the reviewer’s suggestion, we have replaced Figure 1B with S1A after making some updates, including the addition of detailed lists of programs used at each step.
----

3) When comparing N50 across different genomes/assemblies of varying sizes, you may want to use the NG50 metric as see in the assemblathon package.

A:
We appreciate this comment. In this study, although we used genome assembly results uploaded at NCBI, there is no guarantee that the sum of the resulting scaffolds or chromosomes is the same as the actual genome size due to unknown gaps between scaffolds. Hence, we changed the metric from N50 to NG50 using the estimated genome sizes acquired from the references in Figure 1C, as the reviewer suggested. However, for three genome assemblies (Little egret, Hoatzin, and Golden-collared manakin), for which there is no information about the estimated genome size in either the related paper or at NCBI Genome, the genome sizes were estimated with a k-mer counting method using KMC 2 (Deorowicz et al., 2015).
----

4) Which BUSCO dataset version did you use, odb9?

A:
We used OrthoDB v9 as the BUSCO dataset. We thus changed the text from "using BUSCO [22]" to "using BUSCO [49] with OrthoDB v9 (http://www.orthodb.org/)" on page 8 line 5-6.
----

5) As the scaffolding was reference guided, breaks were made, were joins also done using Galgal4 alignments? From the primary manuscript the overall N50 jumped from 10.7Mb to 18.7 Mb (initial ALLPATHS scaffold to pseudo contig/scaffold), but it sounds like it was breaks that did this. You may want to add a sentence to clear this up by saying the intermediate scaffolding and gapfilling pushed the N50 to 27Mb before breaking after aligning to Galgal4 down to 18.7Mb

A:
The scaffold N50 of the initial ALLPATHS-LG assembly was 10.7Mb, which jumped up to 27.8Mb after scaffolding and gap-filling using PacBio long-read and short-read data. In this step, no reference genome was used. As we found some mis-assemblies by genome-to-genome alignment (Figure S2A), breaks were made in the mis-assemblies. After breaking these mis-assemblies, the scaffold N50 was down to 18.7Mb. Given these results, we updated the description from

"Next, the scaffolds were additionally connected with corrected PacBio long reads and FOSMID reads using SSPACE-LongRead [15] and OPERA [16]. The gaps within and between scaffolds were re-examined with GapCloser [12] with error-corrected short reads. All resulting scaffolds were aligned to the galGal4 genome (GenBank assembly accession: GCA_000002315.2) by LASTZ [17]."
to
"In the second round of scaffolding and gap-filling (after the first scaffolding and gap-filling done during ASM1), the ASM1 scaffolds were connected with corrected PacBio long-reads using SSPACE-LongRead [41], and gaps within and between scaffolds were examined with error-corrected short-reads using GapCloser [38]. Then, the gap-filled scaffolds were connected again with FOSMID reads using OPERA [42] and the remaining gaps were re-examined with error-corrected short-reads using GapCloser, resulting in scaffolds with an

N50 length of 27.8Mbp. However, some mis-assemblies (as illustrated in Figure S2A) were found by alignment of the resulting scaffolds with the galGal4 genome (GenBank assembly accession: GCA_000002315.2) using LASTZ [43].” on from page 6 line 20 to page 7 line 3.

----

6) Figure 3 shows Chicken(galGal5) twice, I expect one should be Galgal4. Also regarding figure 3, the end discussion section refers to the golden-collared manakin genome in figure 3, but this does not exist.

A:
Thank you for the comment. We fixed the typo and added golden-collared manakin in Figure3. Additionally, we found other typos and fixed them.

----

7) Figure 7, PCA plots, instead of having text as the components (which gets mixed up and illegible), consider using coloured data points which correspond to a legend.

A:
As the reviewer’s suggestion, we have moved the text to the legend and used colored data points in Figure 5 (originally Figure 7).

----

8) Data repository - would like to have a README file describing the datasets in repository.

A:
As the reviewer’s suggestion, we have added a README file that describes the datasets in the data repository. The contents of the README file are shown below.

[README]
Whole genome and transcriptome maps of the entirely black native Korean chicken breed Yeonsan Ogye
==

Directory lists:
1 - lncRNA
2 - Protein_coding_gene
3 - RRBS
4 - SNPs_INDELs
5 - Repeat
6 - Genome

Files:
1. lncRNA/
ogye1.1_lncRNA_annotation.gtf
lncRNA annotations comprising 6,900 novel and known lncRNAs identified from 20 tissues of Ogye

ogye1.1_lncRNA_expression_table.txt
Tab-delimited expression table of lncRNAs. Fragments Per Kilobase of exons per million mapped reads (FPKM) was calculated using the RSEM program. The values across tissues were normalized using the quantile normalization method.

2. Protein_coding_gene/
ogye1.1_PCG_annotation.gtf
Protein-coding gene annotations comprising 15,766 genes identified from 20 tissues.

ogye1.1_PCG_CDS.fasta
CDS sequences of ogye1.1 protein-coding genes.

ogye1.1_PCG_GENE.fasta
Gene sequences of ogye1.1 protein-coding genes.

ogye1.1_PCG_SE_expression_table.xlsx
The tab-delimited expression table of protein-coding genes using single-end RNA seq libraries. The expression (FPKM) was calculated using RSEM programThe values across tissues were normalized using the quantile normalization method.

ogye1.1_PCG_PE_expression_table.xlsx
The tab-delimited expression table of protein-coding genes using paired-end RNA seq libraries. The expression (FPKM) was calculated using RSEM programThe values across tissues were normalized using the quantile normalization method.

3. RRBS/
ogye1.1_RRBS_[xxxxxx].bedgraph, where [xxxxxx] is a tissue name.
Tab-delimited CpG methylation signal across 20 tissues. The signals were calculated using the Bismark CpG coverage report, which Include 1-based genomic coordinates for every covered cytosine position in each sample in the following format: .

4. SNPs_INDEL/
ogye1.1.total.snps.filtered.vcf
SNP and insertion and deletion (INDEL) were called using VarScan 2 with options -mfileup2snp --min-coverage 8 --min-reads2 2 --min-avg-qual 15 --min-var-freq 0.2 --p-value 1e-2.

ogye1.1.total.indels.filtered.vcf
SNP and insertion and deletion (INDEL) were called using VarScan 2 with options -mfileup2indel --min-coverage 8 --min-reads2 2 --min-avg-qual 15 --min-var-freq 0.2 --p-value 1e-2.

5. Repeat/
ogye1.1.fasta.out
Repeat lists identified from an input genome sequence using the RepeatMasker ver.4-0-6.

ogye1.1.fasta.tbl
The summary table of repeats identified from an input genome sequence using the RepeatMasker ver.4-0-6.

6. Genome/
ogye1.1.fasta
A genome sequence of Yeonsan Ogye genome assembly version 1.1., which include chromosome sequences and unplaced sequences.

===
==========

Other changes in the revised manuscript

In the initial assembly, the short-read datasets were originally split into two groups, each of which was used for de novo assembly (ASM1 and AMS2) using ALLPATHS-LG. The results of ASM1 were subjected to the second scaffolding step, whereas the results of ASM2 were used in the pseudo-reference-assisted assembly step. To clarify the methods, we have added a detailed description of the whole genome assembly pipeline, which was missing from the original manuscript, as below.
“Additionally, another assembly (ASM2) was built with 109.2X paired-end and mate-pair reads that were unused in the initial assembly (see Table 1) using ALLPATHS-LG, resulting in 34,539 contigs with an N50 length of 59.2Kbp. The resulting ASM2 contigs were then subjected to the pseudo-reference-assisted assembly step”
on page 6 line 17-20

and changed from
“Next, the scaffolds were additionally connected”
to
“In the second round of scaffolding and gap-filling (after the first scaffolding and gap-filling done during ASM1), the ASM1 scaffolds were connected” on page 6 line 20-21.

Also, we changed from
“Based on the results, super-scaffolding and additional gap-filling was performed by SSPACE-LongRead and PBJelly, respectively, resulting in scaffold N50 of 21.2Mbp (Figure 1C)”
to
“Based on these results, a third round of scaffolding and gap-filling was performed with the long reads and the ASM2 contigs in each chromosome group using SSPACE-LongRead and PBJelly [46], respectively, resulting in a scaffold N50 of 21.2Mbp with 0.85% gaps (Figure S1)”
on page 7 line 15-18.

To reflect these changes, we have modified Figure 1B, with some changes in the polishing and finishing step.

Additionally, we found some errors in the read numbers and coverages of the whole genome

sequencing data in Tables 1 and S1. The original numbers came from partially preprocessed rather than raw data. We thus have updated the numbers throughout the manuscript, Table1 and Table S1 using the estimated genome size of 1.25Gbp. Now, the total coverage of raw short reads is 376.6X and the coverage of preprocessed short reads is 241.1X.

In addition, to clarify our intended meaning of the term "pseudo-contig", we updated our description from
"Breaking scaffolds at the break points resulted in pseudo-contig N50 of 108.6 Kbp and scaffold N50 of 18.7 Mb (Figure S1B). A pseudo-contig is defined by a sequence broken by gaps of >1bp, which are assumed to be gaps or errors."
to
"Breaking scaffolds at the break points resulted in a scaffold N50 length of 18.7Mbp (Figure S1). For contigs, we considered a pseudo-contig, broken at positions where 2 or more contiguous Ns appeared in scaffolds, resulting in a pseudo contig N50 of 108.6Kbp." on page 7 line 5-7.

Minorly, we changed the section title "Result" to "Data description", and the sub-section title "Sample collection and data description" to just "Sample collection".

References

Deorowicz, S., Kokot, M., Grabowski, S., & Debudaj-Grabysz, A. (2015). KMC 2: fast and resource-frugal k-mer counting. Bioinformatics, 31(10), 1569-1576.

Warren, W. C., Hillier, L. W., Tomlinson, C., Minx, P., Kremitzki, M., Graves, T., . . . Cheng, H. H. (2017). A New Chicken Genome Assembly Provides Insight into Avian Genome Structure. G3 (Bethesda), 7(1), 109-117.

Close
