# Supplementary material for: Whole genome and transcriptome maps of the entirely black native Korean chicken breed Yeonsan Ogye
Source: Gigascience. 2018 Jul 11;7(7):giy086. doi: 10.1093/gigascience/giy086 (PMC6065499; doi:10.1093/gigascience/giy086)
Supplement: Reviewer_1_Report_(Original_Submission) -- Robert Kraus — 02-14-2018 Reviewed [file giy086_reviewer_1_report_(original_submission).pdf]

Reviewer Report

Title: Whole genome and transcriptome maps of the entirely black native Korean chicken breed Yeonsan Ogye

Version: Original Submission **Date:** 2/14/2018

Reviewer name: Robert Kraus

Reviewer Comments to Author:

ear editor and authors,Thank you for giving me the opportunity to comment on GIGA-D-17-00321 (Sohn et al. "Whole genome and transcriptome maps of the entirely black native Korean chicken breed Yeonsan Ogye").During the review process it was hard to refer to places in the text because the lines numbers started over all over on each page; and on top of that, pages were not numbered… I counted page number on page 3 of the document that was available to me because the first two pages were info pages from the editorial office. Page three is the one where the abstract starts. E.g., P1L34 means line 34 on that first page. I like the article because it describes a rich and interesting data set of a very interesting chicken breed. We do not only get presented the genome and high quality assembly, but also various transcriptomes and methylomes. This includes not only annotation but also quantitative information and tissue specific expression atlases.I could not find major problems with this article. Analyses seem to be correctly performed and well described. I am indicating below in my specific comments where this is not the case and the authors should re-work their explanations. I further found that the quality of English could be improved. It is OK for now but the article would really benefit from a native speaker reading over it once more (I am not a native speaker myself, though). Where possible I indicated potential changes to the language but could not be exhaustive.Regarding the structure of the article, and here readability in particular, I imagine it will be hard to present this appropriately in a PDF because of the many figures and tables. I would always say: figures and tables can say more than words, but in this case the number of display item is quite excessive. Perhaps the editors would need to revisit this potential issue.Data availability: I understand that this journal has (luckily!) high standards regarding data availability. The pipelines are well illustrated in the paper and also in the Supp Mat. However, not all runtime parameters are specified for all programmes. Often it is mentioned in the text. I am not sure now if this is sufficient, for a regular journal that does not focus so much on the technicalities I would suggest this is sufficient. For this journal, with its special emphasis on technicalities and bioinformatics protocols, I could imagine that a Supp Mat file containing an inventory of all the command incl. their runtime options would be useful. This may be added at the discretion of the Editor of this journal, if otherwise done regularly here.When my comments (above and below) are implemented I suggest publication of this note into GigaScience after the mentioned revisions. Below are smaller, specific comments.Best regards,Robert KrausSpecific comments:P1L34: gallus gallus domesticus -> Gallus gallus domesticusP1L40: I don't think you should present the definition of N50 in the abstractP1L46: The statement about Galgal5 using only long-read technology is not true. It also used previous data to build on - and that includes 1st and 2nd generation sequencing data - plus Illumina (>30x) was used for error correction. Let alone BAC sequencing and the likes.P3L27: substitute "constructed" with "assembled"P3L47: include -> includesP4L23: The sequencing library from one bird only or combined?P4L39: The text in the bracket should be changed. It now reads as if most of reads were bad quality. Please clarify whether this was your threshold or observation.P4L41: How do the 163.5x distribute across paired-end and mate-pair data?P4L43: I would not say "scaffold quality", because quality is a complex thing. You could be more specific, and refer to "scaffold N50", or "improve scaffolding".P4L43: Which PacBio machine was used and which chemistry/version?P4L51: A reference should be given to the Trizol protocol/company where the reagent was purchased.P5L20: Unclear what "qualified" means, consider rephrasingP5L24: tissues, which are -> tissues;P5L29: All tissues from the same bird? The same bird for which genomic DNA was sequenced?P5L37: Again, was it from the same bird(s) as in the previous steps?P5L46: How was ligation assessed?P6L25: In hybrid -> In the hybridP6L36: Why not aligned to Galgal5?P7L26: The mentioning of these stats are the best or second best values is superfluous. Actually, I am sure by now there are better

stats for assemblies of zebra finch or hummingsbird, that were treated with the currently best possible protocols. This doesn't mean your stats are bad, not at all. But the statement does very likely not apply - and surely will not apply anymore within a really short amount of time. Delete it.P7L49: "by at least a different"; should that be …by at least four different SV prediction programs?P8L13: "three possible mechanistic scenarios" -> exlpain what scenariosP8L46: "and other genomes": what other genomes?P9L4: the first word in the first line on this page, "to", should be removed, right?P9L11: was depicted -> is depictedP9L19: The number of SNPs seems little. How does this compare to the Galgal 4/5 or other, actually more outbred bird genomes?P10L11: Where are those 33 genes situated, as evidenced by comparative mapping. Exclusively on microchromosomes?P10L19: annotated -> annotateP11L29: I am not sure what you mean by "which were dynamically changed". Please explain/rephrase.P12L12: It should be "in the genome" and not "in genomes", right?P12L31: Remove the two commas around "then".P12L40: "specifically" should be changed to "specific", I think?P12L42: Should "only" be "one"? This sentence need to be clarified.P12L53: noises -> noiseP12L56: taken account -> taken into accountP13L5: That should be "Discussion" in singular, right?P13L25-30: Cite the Lemur study and/or the MaSuRCA assemblerP13L59: Remove "The" at beginning of sentence.P14L17: gnome -> genomeTable 1: You should also include a row that summarises the total of coverage (x) across all PE and mate-pair, and the total all. The numbers are in the text, but should re-appear in the table.Table 4: Add a column that gives the species name.Table 5: all "site" should become plural, i.e., "sites".Figure 1C: Do I see correctly that is says "Chichen" and not "Chicken" in line 2? Also: Space missing between "Hooded" and "crow". Assemblies mentioned in this table should have footnotes to them and below the table cite the actual scientific reference articles.

Level of Interest

Please indicate how interesting you found the manuscript: An article of importance in its field

Quality of Written English

Please indicate the quality of language in the manuscript: Needs some language corrections before being published

Declaration of Competing Interests

Please complete a declaration of competing interests, considering the following questions:

- Have you in the past five years received reimbursements, fees, funding, or salary from an organisation that may in any way gain or lose financially from the publication of this manuscript, either now or in the future?
- Do you hold any stocks or shares in an organisation that may in any way gain or lose financially from the publication of this manuscript, either now or in the future?
- Do you hold or are you currently applying for any patents relating to the content of the manuscript?
- Have you received reimbursements, fees, funding, or salary from an organization that holds or has applied for patents relating to the content of the manuscript?
- Do you have any other financial competing interests?
- Do you have any non-financial competing interests in relation to this paper?

If you can answer no to all of the above, write 'I declare that I have no competing interests' below. If your reply is yes to any, please give details below.

none

I agree to the open peer review policy of the journal. I understand that my name will be included on my report to the authors and, if the manuscript is accepted for publication, my named report including any attachments I upload will be posted on the website along with the authors' responses. I agree for my report to be made available under an Open Access Creative Commons CC-BY license (http://creativecommons.org/licenses/by/4.0/). I understand that any comments which I do not wish to be included in my named report can be included as confidential comments to the editors, which will not be published.

I agree to the open peer review policy of the journal

To further support our reviewers, we have joined with Publons, where you can gain additional credit to further highlight your hard work (see: https://publons.com/journal/530/gigascience). On publication of this paper, your review will be automatically added to Publons, you can then choose whether or not to claim your Publons credit. I understand this statement. Yes
