# Supplementary material for: Whole genome and transcriptome maps of the entirely black native Korean chicken breed Yeonsan Ogye
Source: Gigascience. 2018 Jul 11;7(7):giy086. doi: 10.1093/gigascience/giy086 (PMC6065499; doi:10.1093/gigascience/giy086)
Supplement: Reviewer_2_Report_(Original_Submission) -- William Chow — 2/20/2018 Reviewed [file giy086_reviewer_2_report_(original_submission).pdf]

Reviewer Report

Title: Whole genome and transcriptome maps of the entirely black native Korean chicken breed Yeonsan Ogye

Version: Original Submission **Date:** 2/20/2018

Reviewer name: William Chow

Reviewer Comments to Author:

1) Why wasn't Galgal5 used in the overall analyses? (only Galgal4 used) It was presented in the beginning and end of the manuscript.2) Figure 1B is referenced quite often in the manuscript, however the problem with 1B is that the figure is very simple and not detailed enough. You may want to break up 1B into its own figure, and substitute it with a variation of S1B.3) When comparing N50 across different genomes/assemblies of varying sizes, you may want to use the NG50 metric as see in the assemblathon package.4) Which BUSCO dataset version did you use, odb9?5) As the scaffolding was reference guided, breaks were made, were joins also done using Galgal4 alignments? From the primary manuscript the overall N50 jumped from 10.7Mb to 18.7 Mb (initial ALLPATHS scaffold to pseudo contig/scaffold), but it sounds like it was breaks that did this. You may want to add a sentence to clear this up by saying the intermediate scaffolding and gapfilling pushed the N50 to 27Mb before breaking after aligning to Galgal4 down to 18.7Mb6) Figure 3 shows Chicken(galGal5) twice, I expect one should be Galgal4. Also regarding figure 3, the end discussion section refers to the golden-collared manakin genome in figure 3, but this does not exist.7) Figure 7, PCA plots, instead of having text as the components (which gets mixed up and illegible), consider using coloured data points which correspond to a legend. 8) Data repository - would like to have a README file describing the datasets in repository.

Level of Interest

Please indicate how interesting you found the manuscript: An article whose findings are important to those with closely related research interests

Quality of Written English

Please indicate the quality of language in the manuscript: Acceptable

Declaration of Competing Interests

Please complete a declaration of competing interests, considering the following questions:

- Have you in the past five years received reimbursements, fees, funding, or salary from an organisation that may in any way gain or lose financially from the publication of this manuscript, either now or in the future?
- Do you hold any stocks or shares in an organisation that may in any way gain or lose financially from the publication of this manuscript, either now or in the future?
- Do you hold or are you currently applying for any patents relating to the content of the manuscript?

- Have you received reimbursements, fees, funding, or salary from an organization that holds or has applied for patents relating to the content of the manuscript?
- Do you have any other financial competing interests?
- Do you have any non-financial competing interests in relation to this paper?

If you can answer no to all of the above, write 'I declare that I have no competing interests' below. If your reply is yes to any, please give details below.

I am involved with the Genome Reference Consortium, whose goal includes improving/maintaining the International Chicken Genome Consortium's reference genome (Galgal5).

I agree to the open peer review policy of the journal. I understand that my name will be included on my report to the authors and, if the manuscript is accepted for publication, my named report including any attachments I upload will be posted on the website along with the authors' responses. I agree for my report to be made available under an Open Access Creative Commons CC-BY license (http://creativecommons.org/licenses/by/4.0/). I understand that any comments which I do not wish to be included in my named report can be included as confidential comments to the editors, which will not be published.

I agree to the open peer review policy of the journal

To further support our reviewers, we have joined with Publons, where you can gain additional credit to further highlight your hard work (see: https://publons.com/journal/530/gigascience). On publication of this paper, your review will be automatically added to Publons, you can then choose whether or not to claim your Publons credit. I understand this statement. Yes
